# Diterpenoids target SARS-CoV-2 RdRp from the roots of *Euphorbia fischeriana* Steud

**DOI:** 10.3389/fpls.2024.1425759

**Published:** 2024-07-25

**Authors:** Ting Ruan, Zheng-Rui Xiang, Yun-Wu Zhang, Shi-Rui Fan, Juan Ren, Qian Zhao, Xiao-Long Sun, Shi-Li Wu, Li-Li Xu, Miao Qiao, Chen-Xu Jing, Xiao-Jiang Hao, Duo-Zhi Chen

**Affiliations:** ^1^ State Key Laboratory of Phytochemistry and Plant Resources in West China, Kunming Institute of Botany, Chinese Academy of Sciences, Kunming, China; ^2^ Yunnan Characteristic Plant Extraction Laboratory, Kunming, China; ^3^ Research Unit of Chemical Biology of Natural Anti-Virus Products, Chinese Academy of Medical Sciences, Beijing, China; ^4^ Kunming College of Life Science, University of Chinese Academy of Sciences, Kunming, China; ^5^ Department of Chemical Science and Engineering, Yunnan University, Kunming, China; ^6^ Institute of International Rivers and Eco-Security, Yunnan University, Kunming, China; ^7^ Research Center of Traditional Chinese Medicine, The Affiliated Hospital to Changchun University of Chinese Medicine, Changchun, China

**Keywords:** *Euphorbia fischeriana* Steud, diterpenoids, antiviral, SARS-CoV-2 RdRp, microscale thermophoresis

## Abstract

**Introduction:**

Currently, the development of new antiviral drugs against COVID-19 remains of significant importance. In traditional Chinese medicine, the herb *Euphorbia fischeriana* Steud is often used for antiviral treatment, yet its therapeutic effect against the COVID-19 has been scarcely studied. Therefore, this study focuses on the roots of *E. fischeriana* Steud, exploring its chemical composition, antiviral activity against COVID-19, and the underlying basis of its antiviral activity.

**Methods:**

Isolation and purification of phytochemicals from *E. fischeriana* Steud. The elucidation of their configurations was achieved through a comprehensive suite of 1D and 2D NMR spectroscopic analyses as well as X-ray diffraction. Performed cytopathic effect assays of SARS-CoV-2 using Vero E6 cells. Used molecular docking to screen for small molecule ligands with binding to SARS-CoV-2 RdRp. Microscale thermophoresis (MST) was used to determine the dissociation constant Kd.

**Results:**

Ultimately, nine new ent-atisane-type diterpenoid compounds were isolated from *E. fischeriana* Steud, named Eupfisenoids A-I (compounds 1-9). The compound of 1 was established as a C-19-degraded ent-atisane-type diterpenoid. During the evaluation of these compounds for their antiviral activity against COVID-19, compound 1 exhibited significant antiviral activity. Furthermore, with the aid of computer virtual screening and microscale thermophoresis (MST) technology, it was found that this compound could directly bind to the RNA-dependent RNA polymerase (RdRp, NSP12) of the COVID-19, a key enzyme in virus replication. This suggests that the compound inhibits virus replication by targeting RdRp.

**Discussion:**

Through this research, not only has our understanding of the antiviral components and material basis of *E. fischeriana* Steud been enriched, but also the potential of atisane-type diterpenoid compounds as antiviral agents against COVID-19 has been discovered. The findings mentioned above will provide valuable insights for the development of drugs against COVID-19.

## 1 Introduction

**Figure 1:**
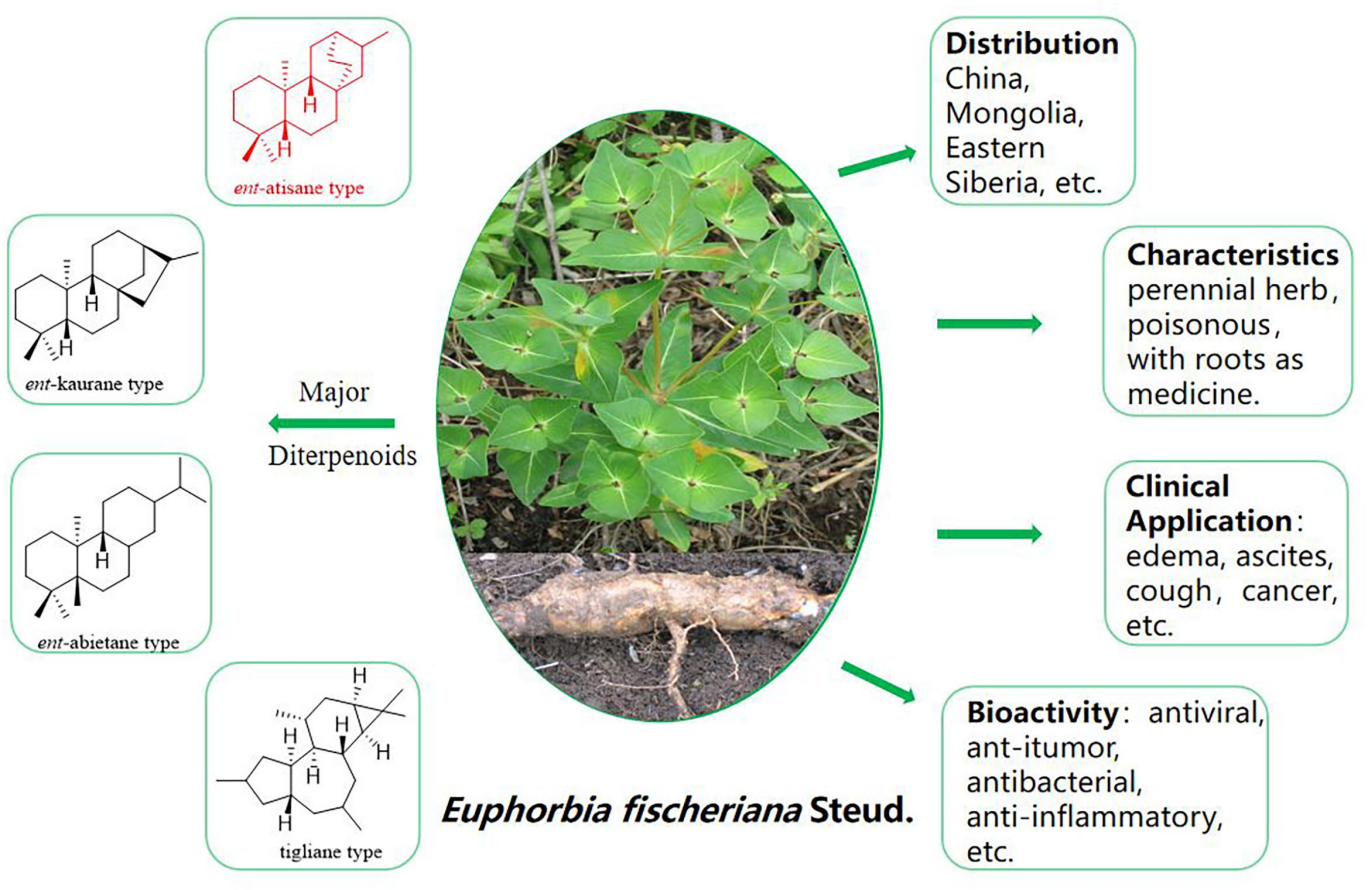
*Euphorbia fischeriana* Steud plant pictures and other information.

**Figure 2:**
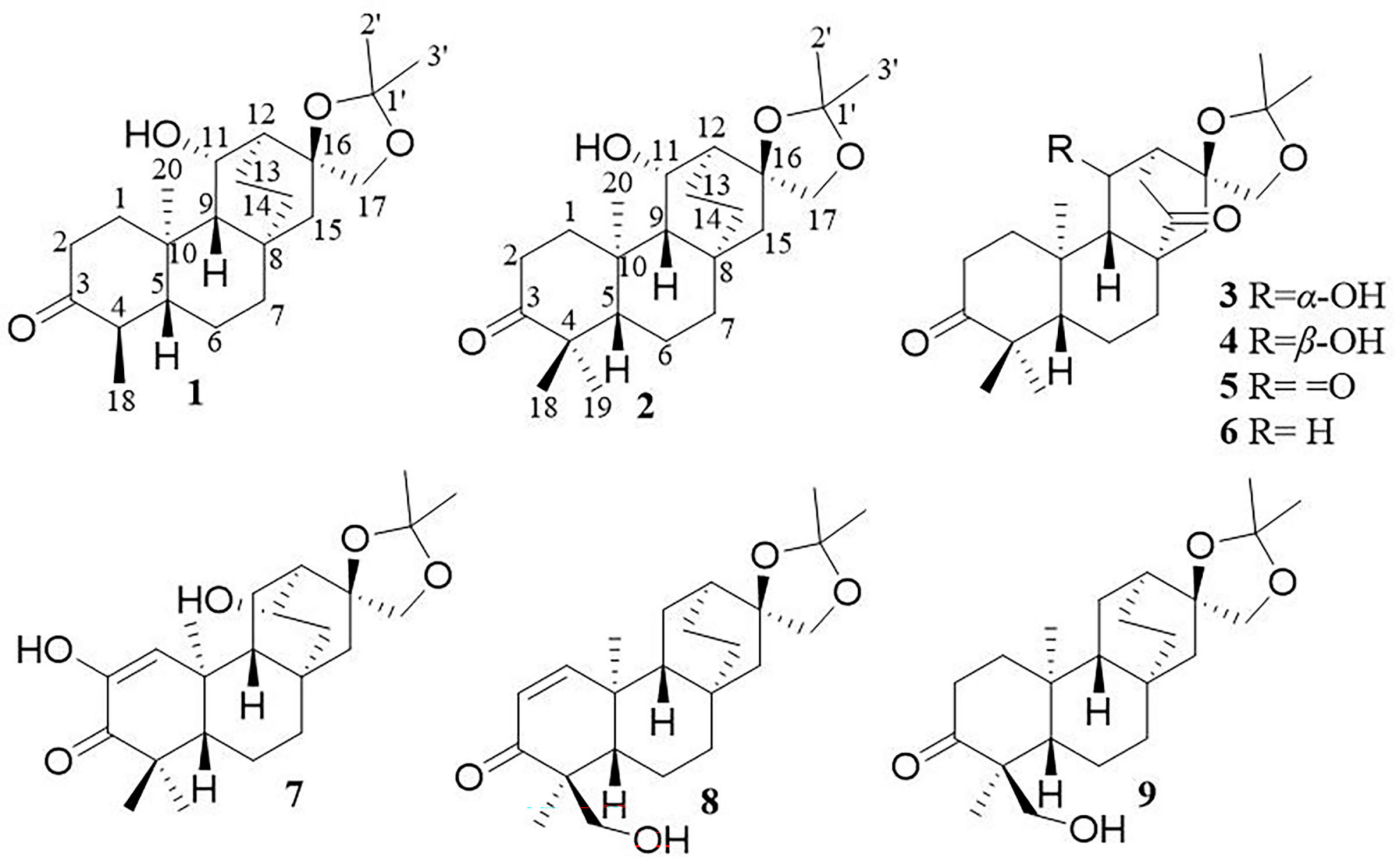
Structures of compounds 1–9.

In late 2019, viral infections spurred by the severe acute respiratory syndrome coronavirus 2 (SARS-CoV-2) profoundly impacted global human existence and productivity (Hu et al., 2021). As of early March 2024 (World Health Organization, 2024), the World Health Organization (WHO) documented 775 million confirmed infections and 7.04 million deaths worldwide. Despite accessible medications and ongoing vaccination drives, viral mutations and waning immune responses have hampered vaccine efficacy, leading to re-infections (Sievers et al., 2022). SARS-CoV-2, an optimistic single-stranded RNA virus (Gorbalenya et al., 2020), employs RNA-dependent RNA polymerase (RdRp) for genome replication and gene transcription (Snijder et al., 2016). RdRp, as a pivotal enzyme in the viral life cycle, stands as a recognized target for antiviral medications (te Velthuis, 2014). Human life and productivity remain imperiled, and the development of targeted COVID-19 medications is urgently needed (Feikin et al., 2022). Natural products have long been esteemed as pivotal sources of medications for diverse ailments. *Euphorbia* is the largest genus of the Euphorbiaceae, one of the largest families of higher plants (Li et al., 2009). *Euphorbia fischeriana* Steud, a member of the genus *Euphorbia* (Euphorbiaceae), is a perennial herb, and the entire plant is poisonous and is grown primarily in Mongolia, Eastern Siberia, and China (Flora of China Editorial Committee, 1997). *E. fischeriana* Steud serves prominently in traditional Chinese medicine, the roots of which has a long history of traditional use for treating conditions such as edema, ascites, cough, and cancer, and also exhibits prowess in combating viral infections (Pharmacopoeia Commission of PRC, 2020). Its diterpenoids possess a spectrum of pharmacological activities, including antiviral, anti-tumor, antibacterial, anti-inflammatory, and other therapeutic properties (Li et al., 2021) (**Figure 1**). In traditional Chinese medicine, *E. fischeriana* Steud is an important antiviral herb. During the outbreak of the COVID-19 pandemic, efforts have been made in some regions of China to utilize traditional Chinese herbal remedies, including E. fischeriana Steud, to treat COVID-19 infections, with some degree of success (Dai et al., 2020). Through literature review, it was found that the material basis of *E. fischeriana* Steud’s anti-COVID-19 activity, specifically the antiviral activity of its natural compounds and related mechanisms, has not been studied. Therefore, we selected *E. fischeriana* Steud as the research subject to investigate its chemical composition. We employed the latest techniques in pharmaceutical chemistry research, such as molecular docking and microscale thermophoresis (MST), to evaluate its potential anti-SARS-CoV-2 effects and related targets. Through *in vitro* cellular experiments, we identified *ent*-atisane-type diterpenoid compounds and potential targets among the anti-COVID-19 active herbal constituents, providing new insights for the development of COVID-19 therapeutic drugs. We have successfully isolated and identified nine diterpenoids with a novel structure from the roots of *E. fischeriana* Steud (**Figure 2**); in addition, we evaluated the anti-SARS-CoV-2 activity of the compounds and explored the targets and mechanisms of action.

## 2 Results

### 2.1 Isolated phytochemicals from *Euphorbia fischeriana* Steud

**Figure 3:**
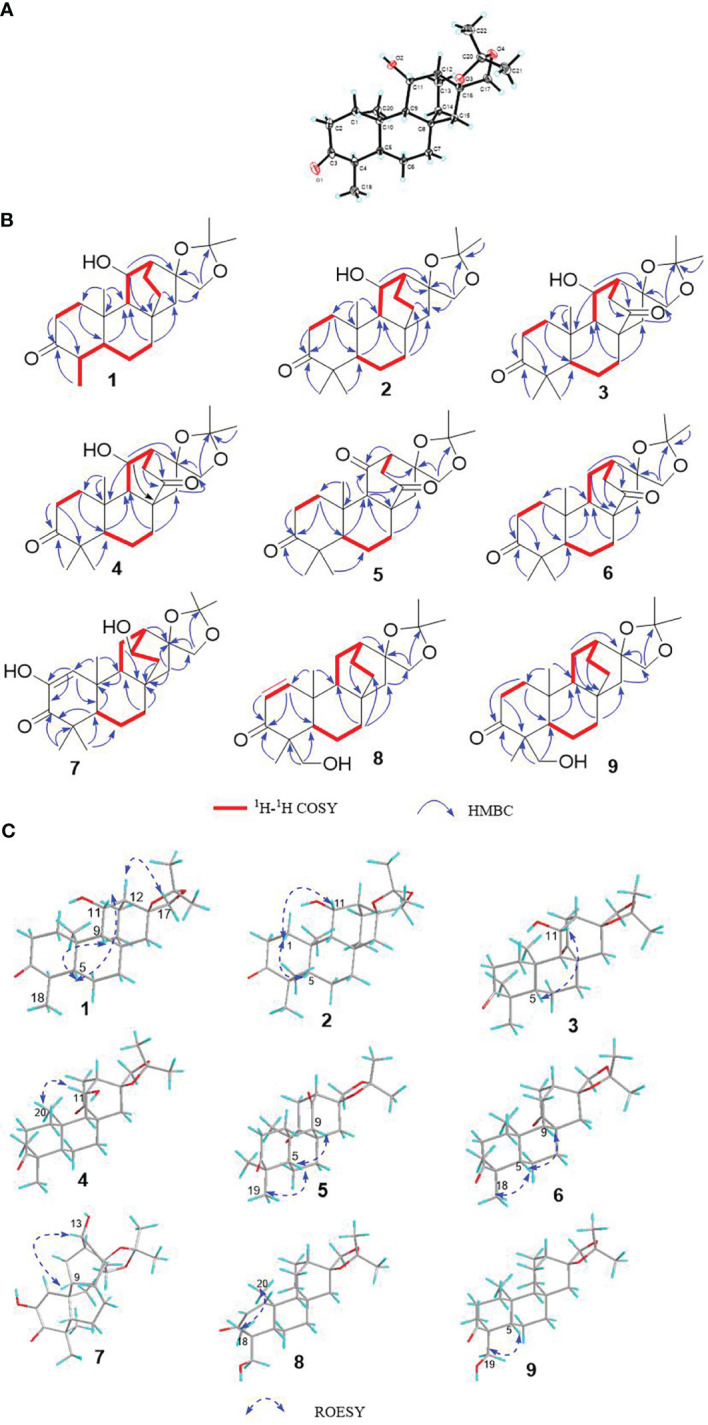
**(A)** Single-crystal x-ray figure structure of compound **1**; **(B, C)** key 2D NMR correlations of compounds 1–9.

Compound 1, white crystals, has the molecular formula C_22_H_34_O_4_ as determined from a high-resolution electrospray ionization mass spectrometry (HR-ESI-MS) peak at *m/z* 363.2529 [M+H]^+^ (calcd. C_23_H_35_O_4_, 363.2530). The molecular formula indicates six indices of hydrogen deficiency. The ^13^C NMR and DEPT (**Tables 1**, **2**) spectra of 1 displayed 22 carbon signals and resonances attributable to four methyls, eight methylenes, five methines (one oxygenated methine), and five quaternary carbons (one carbonyl and two oxygenated carbons), corresponding to the units in its ^1^H NMR data (**Table 1**). The ^1^H-^1^H COSY spectrum of this substance shows the existence of correlations of H-1/H-2 and H-4/H-5. Furthermore, analysis of the HMBC spectrum uncovered correlations of H-1/C-2, C-3 (δ_C_ 213.8), C-5 (δ_C_ 55.5), C-10, and H-4/C-10, H-5/C-3, showing the presence of an A ring with the carbonyl group at C-3. The ^1^H-^1^H COSY spectrum showed a correlation between CH_3_-18/H-4/H-5, and the HMBC spectrum showed a correlation between H-18/C-3, C-4, and C-5, H-1, H-5/C-20, indicating that C-18 (δ_C_ 11.7) and C-20 (δ_C_ 14.8) have a methyl group; C-20 is a horn methyl group. Compound 1 was one methyl less than *ent*-3-oxoatisan-16*α*, 17-acetonide (Yan et al., 2018), suggesting the absence of CH_3_-19 in 1. The ^1^H-^1^H COSY (**Figure 3B**) spectrum showed the correlation of H-9/H-11/H-12 and H-12/H-13/H-14, and the HMBC spectrum showed the correlation of H-11/C-16, C-10, which, combined with the HSQC (**Tables 1**, **2**) (δ_C_ 67.4, C-11, δ_H_ 4.66, H-11), indicated an additional hydroxyl substitution at C-11. The correlation of H-17/C-12, C-15, C-16, C-1′ in the HMBC spectrum (δ_C_ 109.0) demonstrated that C-16 (δ_C_ 81.7) and C-17 (δ_C_ 74.7) form an acetone dimethyl acetal. The ROESY (**Figure 3C**) spectrum of 1 shows the existence of correlations of H-5/H9, H-5/H-11, H-12/H_2_-17, and H-12/H-18, indicating that H-5, H-9, and H-11 are on the same faces of rings B and C. Fortunately, compound 1 crystallizes from methanol and acetone, yielding crystals that are suitable for x-ray diffraction [Cu Kα radiation, Flack parameter 0.01(11)] (**Figure 3A**). Analysis of the structure shows that the absolute configurations at the stereogenic centers in compound 1 is 4*R*,5*S*,8*S*,9*S*,10*R*,11*S*,12*S*,16*S*. Compound 1 was named Eupfisenoid A, and the structure was assigned to be (4*R*,5*S*,8*S*,9*S*,10*R*,11*S*,12*S*,16*S*)*-ent*-11*α*-hydroxy-16*α*,17-acetonide-19-noratisan-3-one.

**Table 1:** ^1^H NMR spectroscopic data (δ) for compounds 1–4.

Position	1*^a^*	2*^b^*	3*^a^*	4*^a^*
1a	2.72, ddd (13.0,6.4, 2.3)	1.93, ddd (13.3, 6.8, 3.1)	2.67, m	2.66, m
1b	1.49, dd(13.9, 10.5)	1.41, m	1.56, m	1.65, m
2a	2.52, td(14.4, 6.4)	2.67, ddd (16.0, 12.5, 6.9)	2.32, ddt (6.6, 3.4)	2.62, m
2b	2.29, m	2.30, ddd (16.0, 5.9, 3.1)	2.19, m	2.34, m
3				
4	2.29, m			
5	1.06, m	1.33, m	1.23, dd(12.4, 2.3)	1.29, dd(12.4, 2.2)
6a	1.49, s	1.76, m	1.62, m	1.62, m
6b	1.31, m	1.30, m	1.43, ddd (11.2, 4.6, 2.4)	1.43, m
7a	1.36, s	1.46, m	2.30, m	2.27, dddd (32.7, 8.6, 5.6, 2.0)
7b	1.10, m	1.31, m	0.75, m	0.84, td(13.5, 4.9)
8				
9	1.39, d	1.76, m	1.60, m	1.92, d(9.8)
10				
11a	4.66, m	3.91, m	4.50, d(5.6, 4.4)	4.95, dd(5.8, 3.9)
11b				
12	1.68, d(2.8)	1.77, m	2.19, m	2.18, m
13a	2.07, m	1.50, m	2.72, m	2.88, dd(19.5, 2.9)
13b	1.36, m	1.24, m	2.59, m	2.01, d(19.5)
14a	2.05, m	1.80, m		
14b	2.00, m	1.32, m		
15a	1.47, s	1.45, m	1.83, d(15.0)	1.74, d(15.1)
15b		1.32, m	1.62, m	
16				
17a	3.98, d(8.4)	4.02, d(8.6)	4.08, d(8.6)	3.93, d(8.7)
17b	3.66, d(8.4)	3.61, d(8.6)	3.69, d(8.6)	3.61, d(8.7)
18	0.98, d(6.5)	1.05, s	0.99, s	1.01, s
19		1.07, s	1.04, s	1.05, s
20	1.41, s	1.12, s	1.13, s	1.14, s
1’				
2’	0.85, td(12.3, 7.2)	1.35, s	1.33, s	1.35, s
3’	1.34, s	1.35, s	1.34, s	1.38, s

^a^ Chemical shifts (ppm) referenced to solvent peak (δ_H_7.26 in CDCl_3_) at 500 MHz.

^b^ Chemical shifts (ppm) referenced to solvent peak (δ_H_ 3.31 in methanol-d_4_) at 500 MHz.

**Table 2:** ^13^CNMR spectroscopic data (δ) for compounds 1–9.

Position	1*^a^*	2*^b^*	3*^a^*	4*^a^*	5*^a^*	6*^a^*	7*^a^*	8*^a^*	9*^a^*
1	40.4	39.5	38.6	38.5	38.2	37.2	128.0	125.4	37.8
2	37.6	35.1	34.4	34.4	34.0	34.1	143.4	160.1	27.6
3	213.8	219.9	217.1	217.0	215.7	216.3	201.0	205.9	219.0
4	44.6	38.5	47.8	47.8	47.8	47.6	43.9	49.0	52.5
5	55.5	56.8	56.7	56.5	55.0	55.4	53.7	47.2	49.3
6	22.3	16.8	20.1	20.2	19.6	19.9	18.9	18.7	19.2
7	38.2	20.8	31.0	31.0	30.9	31.3	39.3	38.7	23.4
8	34.2	48.8	48.7	48.9	49.4	47.8	34.4	33.8	37.0
9	53.3	51.1	56.7	55.7	64.9	51.1	46.0	45.4	50.2
10	37.8	35.0	38.9	38.9	39.3	37.6	39.0	40.1	33.2
11	67.4	67.6	68.2	66.6	210.8	23.8	15.6	23.1	23.2
12	43.8	43.9	47.2	46.7	55.9	36.9	42.9	34.1	34.1
13	16.0	40.5	34.3	35.2	35.1	40.3	67.2	23.3	35.0
14	27.0	39.1	215.9	215.6	212.1	216.2	39.0	28.0	38.7
15	54.5	53.2	50.1	49.2	48.2	50.1	51.8	53.6	53.8
16	81.7	82.6	79.6	80.2	78.3	81.0	81.0	82.5	82.6
17	74.7	74.6	73.3	73.8	73.9	74.1	73.2	74.2	74.4
18	11.7	22.2	21.7	21.8	21.5	21.8	26.9	16.8	16.8
19		26.5	26.6	26.5	26.3	26.0	21.9	67.9	66.8
20	14.8	15.5	15.6	15.6	14.9	12.7	19.7	18.5	13.9
1’	109.0	109.8	109.4	109.8	110.3	109.5	109.0	109.0	108.8
2’	27.1	27.2	27.2	26.6	26.3	26.9	26.8	27.0	27.1
3’	27.3	27.8	26.8	27.3	27.3	27.2	27.6	27.6	27.5

^a^ Chemical shifts (ppm) referenced to solvent peak (δ_C_77.16 in CDCl_3_) at 126 MHz.

^b^ Chemical shifts (ppm) referenced to solvent peak (δ_C_49.00 in methanol-d_4_) at 121 MHz.

Compound 2, white amorphous powder, has the molecular formula C_23_H_36_O_4_ as determined from an HR-ESI-MS peak at *m/z* 399.2512 [M+Na]^+^ (calcd. C_23_H_36_NaO_4_, 399.2506). The molecular formula indicates six indices of hydrogen deficiency. The ^13^C NMR and DEPT (**Tables 1**, **2**) spectra of 2 displayed 23 carbon signals and resonances attributable to five methyls, eight methylenes, four methines, and five quaternary carbons (one carbonyl and two oxygenated carbons). Comparison of literature data analysis revealed that compound 2 is structurally similar to the known compound *ent*-3-oxoatisan-16*α*,17-acetonide (Yan et al., 2018). The OH moiety was attached to C-11 according to the ^1^H-^1^H COSY (**Figure 3B**) correlations of H-9/H/-11/H-12/H-13/H-14 and HMBC spectrum (**Figure 3B**) H-11/C-8 and C-16, combined with HSQC (**Tables 1**, **2**) (δ_C_ 67.6, C-11, δ_H_ 3.91, H-11). The relative configuration of compound 2 was determined by the ROESY spectrum, and the ROESY spectrum (**Figure 3C**) showed a correlation between H-12/H_2_-17, H-5/H-1a, and H-1a/H-11, indicating the *β*-configuration of H-11, suggesting the *α*-orientation of 11-OH. Compound 2 differs from compound 1 in that methyl is added at C-19. Compound 2 showed similar ROESY correlation signals C-12 and C-17, indicating that they share the same relative configuration at C-17. Compound 2 was named Eupfisenoid B, and the structure was assigned to be *ent*-11*α*-hydroxyatisan-16*α*,17-acetonide-3-one (Yan et al., 2018).

Compound 3, colorless crystals, has the molecular formula C_23_H_34_O_5_ as determined from an HR-ESI-MS peak at *m/z* 391.2486 [M+H]^+^ (calcd. C_23_H_35_O_5_,391.2479). The molecular formula indicates seven indices of hydrogen deficiency. The ^13^C NMR and DEPT (**Tables 1**, **2**) spectra of 3 displayed 23 carbon signals and resonances attributable to five methyls, seven methylenes, four methines, and seven quaternary carbons (two carbonyls and two oxygenated carbons). Analysis of the ^1^H and ^13^C NMR data (**Tables 1**, **2**) indicated that the structure of 3 was similar to that of 2. The difference is that compound **3** has another carbonyl. The ^1^H-^1^H COSY spectrum of compound **3** (**Figure 3B**) H-9/H-11/H-12/H-13 and HMBC correlation between H-7, H-12, H-14, and H-15/C-14, combined with HSQC (**Tables 1**, **2**) (δ_C_ 68.2, C-11, δ_H_ 4.50, H-11; δ_C_ 215.9, C-14), revealed a carbonyl group at C-14. The relative configuration of compound **3** was determined by the ROESY spectrum, showing the correlation of H-12/H_2_-17 and H-5/H-11, indicating the *β*-configuration of H-11, and the *α*-orientation of 11-OH can be concluded. Compound 3 showed similar ROESY correlation signals, δ values (δ_C_ 73.3), and *J* values (δ_H_ 4.08, d, *J* = 8.6 Hz, H-17a, δ_H_ 3.69, d, *J* = 8.6 Hz, H-17b) for key protons to compound 1 at C-17, indicating that they share the same relative configuration at C-16 and C-17. Compound 3 was named Eupfisenoid C, and the structure was assigned to be *ent*-11*α*-hydroxyatisan-16*α*,17-acetonide-3,14-dione.

Compound 4, white crystals, has the molecular formula C_23_H_34_O_5_ as determined from an HR-ESI-MS peak at *m/z* 391.2477 [M+H]^+^ (calcd. C_23_H_35_O_5_, 391.2477). Analysis of the ^1^H and ^13^C NMR data (**Tables 1**, **2**) indicated that the structure of 4 was similar to that of 3. The ^1^H-^1^H COSY spectra (**Figure 3B**) show correlations for H-9/H-11/H-12/H-13, and the HMBC spectra show (**Figure 3B**) correlations for H-11/C-8, C-16, which, in combination with the HSQC (**Figure 3B**) (δ_C_ 66.6, C-11, δ_H_ 4.95, *J* = 5.8, 3.9 Hz, H-11; δ_C_ 52.3, C-9, δ_H_ 1.92, d, *J* = 9.8 Hz, H-9), like compound **3**, shows that compound 4 has a hydroxyl group at C-11; the only difference is the configuration of the hydroxyl group. The ROESY spectrum H-12/H_2_-17, H-11/H_3_-20, assigned the *α*-configuration of H-11, suggests the *β*-orientation of 11-OH. Compound 4 showed similar ROESY correlation signals, δ values (δ_C_ 73.8), and *J* values (δ_H_ 3.93, d, *J* = 8.7 Hz, H-17a, δ_H_ 3.61, d, *J* = 8.7 Hz, H-17b) for key protons to compound 1 at C-17, indicating that they share the same relative configuration at C-16 and C-17. The compound was named Eupfisenoid D, and the structure was assigned to be *ent*-11*β*-hydroxyatisan-16*α*,17-acetonide-3,14-dione.

Compound 5, white powder, has a molecular formula of C_23_H_32_O_5_ according to an HR-ESI-MS peak at *m/z* 433.2229 [M+COOH]^−^ (calcd. C_24_H_33_O_7_, 433.2232). The ^13^C NMR and DEPT (**Tables 2**, **3**) spectra of 5 displayed 23 carbon signals, and analysis of the ^1^H and ^13^C NMR data (**Tables 1** **–** **3**) shows that compound 5 and compound 4 are structurally similar. They differ by a decrease in the hydroxyl group and an increase in the carbonyl group of compound 5. The location of the introduced carbonyl group was attached to C-2 by the correlation of H-12/H-13 in the ^1^H-^1^H COSY spectrum (**Figure 3B**) and H-9, H-12/C-11 (δ_C_ 210.8) in the HMBC spectrum (**Figure 3B**), in combination with the HSQC (**Tables 2**, **3**) (δ_C_ 64.9, C-9, δ_H_ 1.88, H-9; δ_C_ 55.9, C-12, δ_H_ 2.80, H-12). Compound **5** showed similar ROESY (**Figure 3C**) correlation signals, δ values (δ_C_ 73.9), and *J* values (δ_H_ 3.93, d, *J* = 8.8 Hz, H-17a, δ_H_ 3.64, d, *J* = 8.8 Hz, H-17b) for key protons to compound 1 at C-17, indicating that they share the same relative configuration at C-16 and C-17. The compound was named Eupfisenoid E, and the structure was assigned to be *ent*-atisan-16*α*,17-acetonide-3,14-dione.

**Table 3:** ^1^H NMR spectroscopic data (δ) for compounds 5–9.

Position	5*^a^*	6*^a^*	7*^a^*	8*^a^*	9*^a^*
1a	2.81, m	1.81, m	6.31, s	5.83, d(10.1)	1.86, m
1b	1.68, d(4.4)	1.34, s			
2a	2.52, m	2.65, ddd (15.8, 13.3, 6.4)		7.02, d(10.1)	1.87, m
2b	2.40, m	2.31, m			0.81, m
3					
4					
5	1.34, m	1.24, dd (12.4, 2.5)	1.63, dt, (18.8, 8.4)	1.91, m	1.64, dd (11.9, 2.0)
6a	1.65, d(3.5)	1.63, m	1.49, m	1.53, m	1.49, m
6b	1.53, dd (13.5, 2.8)	1.48, ddd (17.9, 7.9, 4.5)		1.45, m	1.35, m
7a	2.39, m	2.31, m	1.52, m	1.46, m	1.50, m
7b	0.97, m	0.82, m	1.27, d, (13.9)	1.27, m	
8					
9	1.87, t(7.6)	1.43, m	1.6, m	1.64, dd(10.6, 7.5)	1.36, m
10					
11a		1.79, m	1.97, dd, (17.6, 6.7)	2.20, t(12.0)	2.01, dd (13.1, 11.6)
11b		1.62, m	1.87, d	1.41, m	1.23, m
12	2.80, m	2.20, m	1.90, t, (9.9)	1.81, m	1.74, m
13a	3.00, dd (19.5, 2.6)	2.76, dt (19.1, 2.8)	3.97, d, (8.9)	1.53, m	2.64, ddd (16.0, 13.9, 6.5)
13b	2.43, m	2.16, td (6.2, 2.9)			2.27, ddd (16.0,5.0,2.5)
14a			1.77, d, (14.1)	1.87, m	1.47, m
14b			1.36, s	0.87, m	1.23, m
15a	2.13, d(15.2)	1.90, d(14.8)	1.40, s	1.51, s	1.46, m
15b	1.86, t(7.6)	1.69, d(14.8)			
16					
17a	3.93, d(8.8)	4.06, d(8.5)	4.02, d, (8.5)	3.99, d(8.4)	3.97, d (8.3)
17b	3.64, d(8.8)	3.75, d(8.5)	3.57, d, (8.5)	3.62, d(8.4)	3.60, s
18	0.97, s	1.00, s	1.21, s	1.07, s	1.01, s
19a	1.07, s	1.07, s	1.11, s	3.71d(10.7)	3.63, m
19b				3.44, d(10.7)	3.37, dd (11.3, 6.7)
20	0.87, s	0.86, s	1.36, s	1.28, s	1.18, s
1’					
2’	1.33, s	1.33, s	1.39, s	1.37, s	1.36, s
3’	1.36, s	1.34, s	1.38, s	1.37, s	1.37, s

^a^ Chemical shifts (ppm) referenced to solvent peak (δ_H_7.26 in CDCl_3_) at 500 MHz.

Compound 6, colorless crystals, has the molecular formula C_23_H_34_O_4_ as determined from an HR-ESI-MS peak at *m/z* 373.2382 [M-H]^−^ (calcd. C_23_H_33_O_4_, 373.2384). The ^13^C NMR and DEPT (**Tables 2**, **3**) spectra of 6 displayed 23 carbon signals, and the analysis of the ^1^H and ^13^C NMR data (**Tables 2**, **3**) indicated that the structure of 6 was similar to that of 5. In the ^1^H-^1^H COSY spectrum (**Figure 3B**), there is a correlation between H-9/H-11/H-12/H-13, and in combination with the HSQC (**Tables 2**, **3**) (δ_C_ 23.8, C-11, δ_H_ 1.79, 1.62, H-11; δ_C_ 36.9, C-12, δ_H_ 2.20, H-12), compound 6 has one less carbonyl group at C-11 compared to compound 5. Compound 6 showed similar ROESY (**Figure 3C**) correlation signals, δ values (δ_C_ 74.1), and *J* values (δ_H_ 4.06, d, *J* = 8.5 Hz, H-17a, δ_H_ 3.75, d, *J* = 8.5 Hz, H-17b) for key protons to compound 1 at C-17, indicating that they share the same relative configuration at C-16 and C-17. Compound 6 was named Eupfisenoid F, and the structure was assigned to be *ent*-atisan-16*α*,17-acetonide-3-one.

Compound 7, colorless needle crystals, has the molecular formula C_22_H_32_O_5_ as determined from an HR-ESI-MS peak at *m/z* 435.2387 [M+COOH]^−^ (calcd. C_23_H_35_O_7_ 435.2388). The ^13^C NMR and DEPT (**Tables 2**, **3**) spectra of **7** displayed 23 carbon signals. By comparing literature data, it was determined that compound **7** has two more hydroxyl groups compared to the *ent*-3-oxoatis-1-en-16*α*,17-acetonide (Huang, 2021). ^1^H-^1^H COSY spectra (**Figure 3B**) showed a correlation of H-9/H-11/H-12/13, which, in combination with HMBC spectra (**Figure 3B**), shows a correlation between H-1/C-2/C-3, C-5, and C-20, indicating a hydroxyl group at the C-2 position of the double bond. From the HSQC (**Tables 2**, **3**) (δ_C_ 67.2, C-13, δ_H_ 3.96, H-13; δ_C_ 46.0, C-9, δ_H_ 1.60, H-9), combined with the ^1^H-^1^H COSY spectra, we can determine another hydroxyl group at C-13. The relative configuration of compound 7 can be inferred by analyzing the ROESY correlation signals. According to the ROESY spectrum (**Figure 3C**), the correlation signals of H-12/H_2_-17 and H-13/H-9 indicate the *β*-configuration of H-13, suggesting the *α*-orientation of 13-OH. Compound **7** showed similar ROESY correlation signals, δ values (δ_C_ 73.2), and *J* values (δ_H_ 4.02, d, *J* = 8.5 Hz, H-17a, δ_H_ 3.57, d, *J* = 8.5 Hz, H-17b) for key protons to compound 1 at C-17, indicating that they share the same relative configuration at C-16 and C-17. Compound 7 was named Eupfisenoid G, and the structure was assigned to be *ent*-2,13*α*-dihydroxyatisan-1-en-16*α*,17-acetonide-3-one.

Compound 8, white powder, has the molecular formula C_23_H_34_O_4_ as determined from an HR-ESI-MS peak at *m/z* 375.2536 [M+H]^+^ (calcd. C_23_H_35_O_4_, 375.2530). The ^13^C NMR and DEPT (**Tables 2**, **3**) spectra of 8 displayed 23 carbon signals and resonances attributable to four methyls, eight methylenes (one oxygenated methylene), five methines, and six quaternary carbons (one carbonyl and two oxygenated carbons). Comparison of literature data analysis revealed that compound 8 is structurally similar to the known compound *ent*-atisane-16*β*,17-isopropylidenedioxy-19-ol-3-one (Kuang et al., 2017), the ^1^H-^1^H COSY (**Figure 3B**) spectra of H-1/H-2 correlation exists, and H-1/C-3, C-5, H-2/C-3, H2-19/C-4, C-5 correlation exists in the HMBC spectra (**Figure 3B**); combined with the HSQC (**Tables 2**, **3**) (δ_C_ 47.2, C-5, δ_H_ 1.91, H-5; δ_C_ 67.9, C-19, δ_H_ 3.71, d, *J* = 10.7 Hz, 3.44, d, *J* = 10.9 Hz, H-19) and ^13^C NMR spectra and DEPT spectra (**Tables 2**, **3**) (δ_C_ 125.4, C-1; δ_C_ 160.1, C-2), an extra double bond at C-1 and C-2 can be seen. The relative configuration of compound 8 was determined by the ROESY spectrum, and the ROESY spectrum (**Figure 3C**) showed a correlation of H-12/H_2_-17 and H**_3_**-18/H_3_-20, which shows the *α*-configuration of H**_3_**-18, and the *β*-orientation of H_2_-19 can be concluded. Compound 8 showed similar ROESY correlation signals, δ values (δ_C_ 74.2), and *J* values (δ_H_ 3.99, d, *J* = 8.4 Hz, H-17a, δ_H_ 3.62, d, *J* = 8.4 Hz, H-17b) for key protons to compound 1 at C-17, indicating that they share the same relative configuration at C-16 and C-17. Compound 8 was named Eupfisenoid H, and the structure was assigned to be *ent*-atisan-1-en-16*α*,17-acetonide-19-hydroxy-3-one.

Compound 9, white powder, has the molecular formula C_23_H_34_O_4_ based on its HR-ESI-MS peak at *m/z* 399.2510 [M+Na]^+^ (calcd. C_23_H_36_NaO_4_, 399.2506). The ^13^C NMR and DEPT (**Tables 2**, **3**) spectra of 9 displayed 23 carbon signals. Analysis of the ^1^H and ^13^C NMR data shows differences between compounds 8 and 9 and the no double-bond carbon substitution at C-1 and C-2 of 9. The relative configuration was determined by the ROESY spectrum, and the ROESY spectrum (**Figure 3C**) showed a correlation between H_3_-18/H_3_-20, establishing the *α*-configuration of H_3_-18 and suggesting the *β*-orientation of H_2_-19. Compound 9 showed similar ROESY correlation signals, δ values (δ_C_ 74.4), and *J* values (δ_H_ 3.97, d, *J* = 8.3 Hz, H-17a, δ_H_ 3.60, s, H-17b) for key protons to compound 1 at C-17, indicating that they share the same relative configuration at C-16 and C-17. Compound 9 was named Eupfisenoid I, and the structure was assigned to be *ent*-atisan-16*α*,17-acetonide-19-hydroxy-3-one.

**Figure 4:**
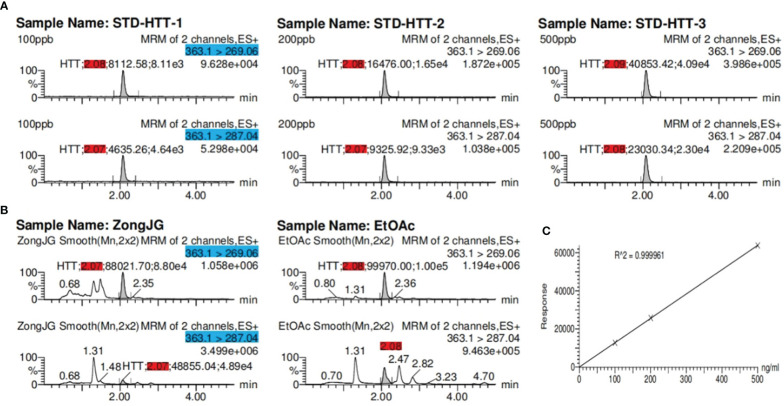
The UPLC-MS/MS results for sample. **(A)** Retention time (red) of compound at three concentrations; **(B)** retention time (red) of compound 1 at methanol extract and ethyl acetate extraction layer. The blue part represents the precursor and product ions of compound 1. **(C)** Coefficient of determination: *R* ^2^ = 0.999961 of compound 1.

Given the presence of a ketal carbon in the compound, we initially suspected that it might be an artifact. To investigate further, using a Waters Acquity ultraperformance liquid chromatograph (UPLC) tandem Xevo TQ-S micro triple quadrupole mass spectrometer, we conducted ultraperformance liquid chromatography-tandem mass spectrometry (UPLC-MS/MS) (Sun et al., 2023) quantitative analysis assays for compound 1, methanol extract, and the ethyl acetate extraction layer (**Figure 4**).

The experimental findings revealed the presence of compound 1 in both the methanol and ethyl acetate extracts, with the ethyl acetate extraction layer containing a higher quantity of compounds than the methanol extract (**Table 4**). This substantiates that compound 1 is not an artificial product but rather a natural plant-derived compound.

**Table 4:** The UPLC-MS/MS results for sample.

Sample name	Type	Std. Conc	RT	Area	Response	ng/mL	%Dev
STD-HTT-1	Standard	100.00	2.08	8,112.583	12,747.847	99.7	−0.3
STD-HTT-2	Standard	200.00	2.08	16,476.002	25,801.927	201.7	0.8
STD-HTT-3	Standard	500.00	2.09	40,853.418	63,883.760	499.4	−0.1
ZongJG	Analyte		2.07	88,021.695	136,876.738	1070.0	
EtOAc	Analyte		2.08	99,970.000	156,843.273	1226.1	

### 2.2 Bioassay results for compound 1

**Figure 5:**
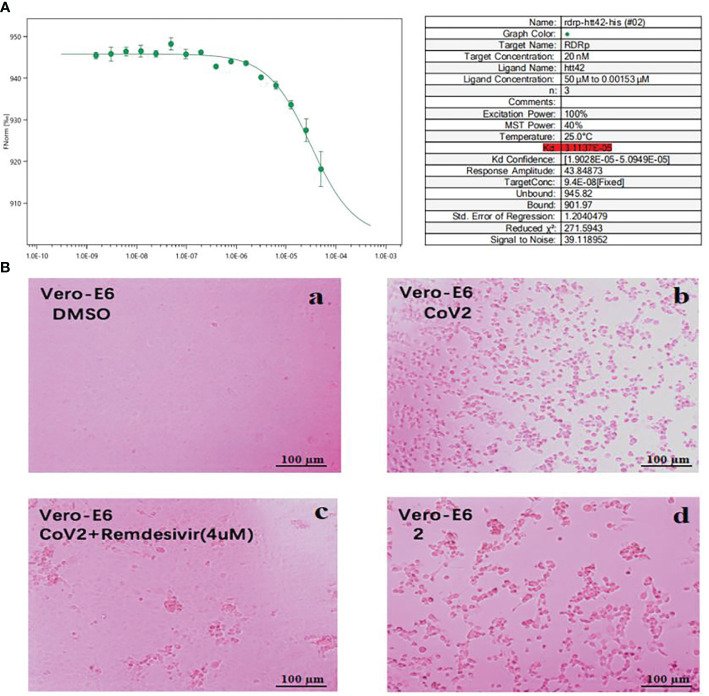
**(A)** MST analysis results of compound 1. The linear fit is close to S, and the *K*_d_ = 31.13 μM. **(B)** Cytopathic effects of SARS-CoV-2-infected Vero E6 cells. (a) Vero E6 cells were treated with a DMSO solution. (b) Vero E6 cells were then infected with SARS-CoV-2; (c) SARS-CoV-2 + Remdesivir; (d) SARS-CoV-2 + compound 1 (because the compounds were screened in different batches, 2 in the figure actually represents compound 1; concentration was 20 μM).

**Figure 6:**
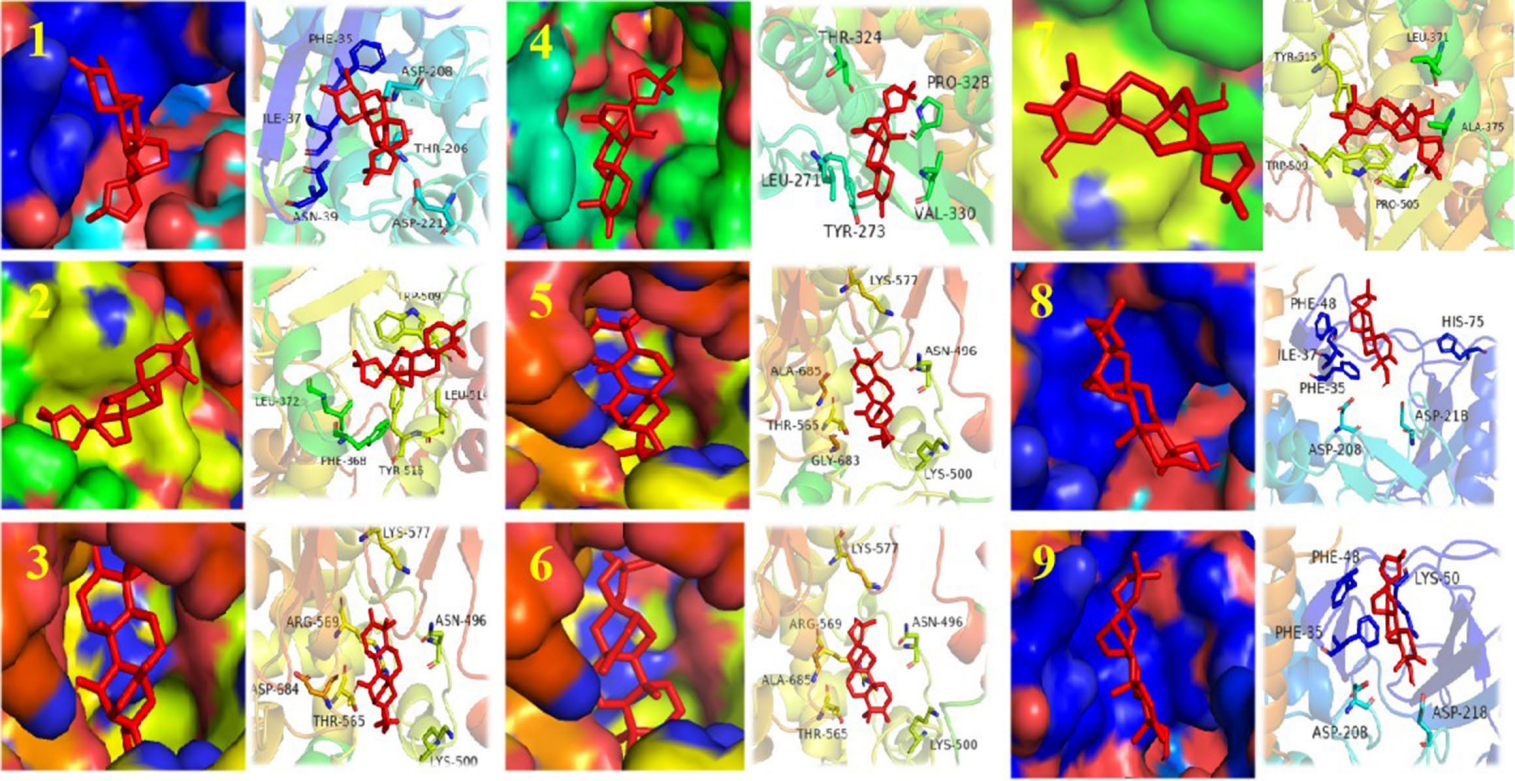
Structure of compounds 1–9 bound to SARS-CoV-2 Rdrp. The compound is denoted in red. The left shows the state of the compound in the substrate-binding pocket of the SARS-CoV-2 Rdrp. The right shows the co-crystal structures of the compound with SARS-CoV-2 Rdrp.

We performed cytopathic effect assays of SARS-CoV-2 using Vero E6 cells with remdesivir as a positive control to further understand the biological activity of compound 1. In DMSO (**Figure 5B**a) as naive control, the Vero E6 cells were morphologically intact. SARS-CoV-2-infected Vero E6 cells showed a morphological deformation with chromatin condensation and karyopycnosis (**Figure 5B**b), demonstrating a significant cytopathic effect. The addition of compound 1 reduced the number of cells that developed lesions in comparison to before (**Figure 5B**c), but not as much as remdesivir (**Figure 5B**d). Thus, assays of cytopathic effect clearly show the anti-SARS-CoV-2 activity of compound 1 (Zhao et al., 2023) (**Figure 5B**). We used molecular docking to screen for small-molecule ligands with better binding to SARS-CoV-2 RdRp. The molecular docking (Morris et al., 2008) results are as follows (**Figure 6**): compound 1 (−8.0 kcal/mol), compound 2 (−7.7 kcal/mol), compound 3 (−8.3 kcal/mol), compound 4 (−7.7 kcal/mol), compound 5 (−7.7 kcal/mol), compound 6 (−7.3 kcal/mol), compound 7 (−7.9 kcal/mol), compound 8 (−7.1 kcal/mol), and compound 9 (−7.4 kcal/mol). Compound 1 demonstrated tight binding to SARS-CoV-2 RdRp, with binding energies of −8.0 kcal/mol. This suggests their potential as foundational compounds for specific agents targeting the active site of SARS-CoV-2 RdRp. Considering RdRp as a primary target for anti-SARS-CoV-2 measures, it holds significant promise in discovering potential COVID-19 drugs. Thus, we further assessed the binding of compound 1 and SARS-CoV-2 Rdrp protein. Employing MST (Wienken et al., 2010), a method analyzing molecular movement in a microscopic temperature gradient field, we determined the dissociation constant *K*_d_. The lower *K*_d_ (31.13 μM, **Figure 5A**) values for 1 indicate stronger affinity between compound 1 and SARS-CoV-2 RdRp, further confirming its binding to the SARS-CoV-2 Rdrp protein.

## 3 Materials and methods

### 3.1 General

One- and two-dimensional NMR spectra were determined by Bruker 500-MHz and 600-MHz NMR instruments with the internal standard: TMS. The chemical shifts δ were expressed in parts per million (ppm), and the coupling constant *J* was expressed in Hz. The CD spectra were measured on a photophysical circular dichroism spectrometer (Applied Photophysics, Leatherhead, Surrey, UK). HR-ESI-MS data acquisition was performed in positive mode on an Agilent 1290 UPLC/6540 Q-TOF mass spectrometer. The UV spectra were detected on a Shimadzu UV-2401A UV spectrometer, the specific spin data were detected on a JASCO DIP-370 digital spinometer, and the IR spectra were detected on a Tenor 27 infrared spectrometer with KBr pressurization assay. Single-crystal x-ray diffraction experiments were detected on a Bruker APEX DUO diffractometer with a copper target. Semipreparative HPLC was performed on an Agilent 1260 apparatus equipped with a UV detector and a Zorbax SB-C-18 (Agilent, 9.4 mm × 25 cm) column. Column chromatography (CC) was performed using silica gel (200–300 mesh and H, Qingdao Marine Chemical Co. Ltd., Qingdao, China) and RP-C18 gel (40–63 μm, Merck, Darmstadt, Germany). Fractions were monitored by TLC (GF254, Qingdao Marine Chemical Co. Ltd., Qingdao, China), and spots were visualized by heating silica gel plates sprayed with 10% H_2_SO_4_ in EtOH. All solvents were distilled prior to use.

### 3.2 Plant material

The roots of *E. fischeriana* Steud were collected in September 2015 from Xianggelila City, Yunnan Province, People’s Republic of China. The plant samples were identified by Prof. Xun Gong of the State Key Laboratory of Phytochemistry and Plant Resource in West China. Voucher specimens (HXJ20150915) were deposited at Kunming Institute of Botany (KIB), Chinese Academy of Sciences (CAS).

### 3.3 Isolation and purification phytochemicals of *Euphorbia fischeriana* Steud

The air-dried and powdered twigs and roots of *E. fischeriana* Steud were extracted three times with 75% EtOH at room temperature to give a crude extract (1.93 kg). The EtOAc fraction (540 g) was subjected to silica gel CC with a gradient elution of petroleum ether-acetone (50:1 to 20:1) to afford five fractions, A–E. Fraction D (140 g) was subjected to a silica gel column, eluted with petroleum ether-acetone (10:1, 8:2, 7:3, 6:4, 1:1, V/V), to yield 12 fractions (D1–D12), continuation of the fine purification of the compounds.

### 3.4 Purity of the isolated compounds

Fraction D3 (1.1 g) was chromatographed over a column of C18 reversed-phase silica gel (MeOH/H_2_O, 20% to 100%, increase in 5% gradient) to give eight subfractions (D3a–D3h). Fraction D3e (300 mg) was purified by semipreparative HPLC with the mobile phase of 60% MeOH in H_2_O to give compound **3** (40.5 mg, retention time: 35.0 min); using semipreparative HPLC with the mobile phase of 50% CH_3_CN in H_2_O led to the isolation of 6 (4.5 mg, retention time: 45.5 min) and 9 (9.7 mg, retention time: 55.5 min), and similarly, fraction D3h (30 mg) was purified by semipreparative HPLC with the mobile phase of 48% CH_3_CN in H_2_O to give compound 1 (7.3 mg, retention time: 50.0 min). Fraction D4 (10 g) was subjected to a silica gel column, eluted with acetic ether-acetone (60:1 to 1:1), to yield 11 factions (D4a–D4k). Fraction D4c (1.2 g) was chromatographed over a column of C18 reversed-phase silica gel (MeOH/H_2_O, 20% to 100%, increase in 5% gradient) to give four subfractions (D4c1–D4c4). Fraction D4c1 (80 mg) was purified by semipreparative HPLC with the mobile phase of 42% CH_3_CN in H_2_O to give compounds 2 (1.7 mg, retention time: 37.0 min), **7** (10 mg, retention time: 48.6 min), and 8 (7.5 mg, retention time: 65.5 min). Fraction D4i (3 g) was chromatographed over a column of C18 reversed-phase silica gel (MeOH/H_2_O, 30% to 100%, increase in 5% gradient) to give two subfractions (D4i1 and D4i2). Fraction D4i2 (40 mg) was purified by semipreparative HPLC with the mobile phase of 56% CH_3_CN in H_2_O to give compounds 4 (9.6 mg, retention time: 28 min) and 5 (6 mg, retention time: 42 min).

### 3.5 Compound naming rule

The compound name consists of a partial letter of the plant’s Latin name (*E. fischeriana* Steud) and the suffixes of the terpenoids.

### 3.6 Spectral measurements

(4*R*,5*S*,8*S*,9*S*,10*R*,11*S*,12*S*,16*S*)*-ent*-11*α*-hydroxy-16*α*,17-acetonide-19-noratisan-3-one (Eupfisenoid A, compound 1), white crystals (MeOH); [*α*]24.6 D-17.96 (c 0.05, MeOH); UV (MeOH) *λ*_max_ (log ϵ) 203 (3.78) nm; IR (KBr) *ν*_max_ 3,438, 3,397, 2,922, 2,851, 1,734, 1,701, 1,646, 1,455, 1,371, 1,255, 1,171, 1,058, 721 cm^−1^; ^1^H and ^13^C NMR, see **Tables 1**, **2**; HR-ESI-MS peak at *m*/*z* 363.2529 [M+H]^+^ (calcd. C_23_H_35_O_4_, 363.2530).

*Ent*-11*α*-hydroxyatisan-16*α*,17-acetonide-3-one (Eupfisenoid B, compound 2), white amorphous powder; [*α*]23.9 D-48.96 (c 0.25, MeOH); UV (MeOH) *λ*_max_ (log ϵ) 203 (4.03) nm; IR (KBr) *ν*_max_ 3,457, 2,934, 2,855, 1,702, 1,631, 1,448, 1,370, 1,213, 1,121, 1,058, 722 cm^−1^; ^1^H and ^13^C NMR, see **Tables 1**, **2**; HR-ESI-MS peak at *m*/*z* 399.2512 [M+Na]^+^ (calcd. C_23_H_36_NaO_4_, 399.2506).

*Ent*-11*α*-hydroxyatisan-16*α*,17-acetonide-3,14-dione (Eupfisenoid C, compound 3), colorless crystals; [*α*]23.1 D-18.31 (c 0.13, MeOH); UV (MeOH) *λ*_max_ (log ϵ) 203 (4.03) nm; IR (KBr) *ν*_max_ 3,439, 2,945, 2,860, 1,711, 1,692, 1,440, 1,371, 1,216, 1,154, 1,060, 721 cm^−1^; ^1^H and ^13^C NMR, see **Tables 1**, **2**; HR-ESI-MS peak at *m*/*z* 391.2486 [M+ H]^+^ (calcd. C_23_H_35_O_5_,391.2479).

*Ent*-11*β*-hydroxyatisan-16*α*,17-acetonide-3,14-dione (Eupfisenoid D, compound 4), white crystals; [*α*]20 D-1 (c 0.1, MeOH); UV (MeOH) *λ*_max_ (log ϵ) 203.5 (4.07) nm; IR (KBr) *ν*_max_ 3,493, 2,928, 2,869, 1,697, 1,452, 1,374, 1,256, 1,125, 1,074, 720 cm^−1^; ^1^H and ^13^C NMR, see **Tables 1**, **2**; HR-ESI-MS peak at *m*/*z* 391.2477 [M+ H]^+^ (calcd. C_23_H_35_O_5_,391.2477).

*Ent*-atisan-16*α*,17-acetonide-3,14-dione (Eupfisenoid E, compound 5), white powder; [*α*]19.9 D19.06 (c 0.11, MeOH); UV(MeOH) *λ*_max_ (log ϵ) 204 (4.36) nm; IR (KBr) *ν*_max_ 3,425, 2,922, 2,863, 1,716, 1,630, 1,458, 1,373, 1,261, 1,149, 1,060, 722 cm^−1^; ^1^H and ^13^C NMR, see **Tables 2**, **3**; HR-ESI-MS peak at *m*/*z* 433.2229 [M+COOH]^−^ (calcd. C_24_H_33_O_7_, 433.2232).

*Ent*-atisan-16*α*,17-acetonide-3-one (Eupfisenoid F, compound 6), colorless crystals; [*α*]25 D1 (c 0.1, MeOH); UV (MeOH) *λ*_max_ (log ϵ) 203 (4.00) nm; IR (KBr) *ν*_max_ 3,432, 2,937, 2,860, 1,711, 1,638, 1,459, 1,369, 1,249, 1,152, 1,060, 723 cm^−1^; ^1^H and ^13^C NMR, see **Tables 2**, **3**; HR-ESI-MS peak at *m*/*z* 373.2382 [M-H]^−^ (calcd. C_23_H_33_O_4_, 373.2384).

*Ent*-2,13*α*-dihydroxyatisan-1-en-16*α*,17-acetonide-3-one (Eupfisenoid G, compound 7), colorless needle crystals; [*α*]24.6 D-44.62 (c 0.13, MeOH); UV (MeOH) *λ*_max_ (log ϵ) 270 (3.50) nm; IR (KBr) *ν*_max_ 3,439, 2,982, 1,717, 1,648, 1,572, 1,250, 714 cm^−1^; ^1^H and ^13^C NMR, see **Tables 2**, **3**; HR-ESI-MS peak at *m*/*z* 435.2387 [M+COOH]^−^ (calcd. C_24_H_35_O_7_,435.2388).

*Ent*-atisan-1-en-16*α*,17-acetonide-19-hydroxy-3-one (Eupfisenoid H, compound 8), white powder; [*α*]23.3 D-11.86 (c 0.07, MeOH); UV (MeOH) *λ*_max_ (log ϵ) 228 (3.91) nm; IR (KBr) *ν*_max_ 3,437, 2,934, 2,870, 1,667, 1,369, 1,252, 1,156, 1,057, 715 cm^−1^; ^1^H and ^13^C NMR, see **Tables 2**, **3**; HR-ESI-MS peak at *m*/*z* 375.2536 [M+H]^+^ (calcd. C_23_H_35_O_4_, 375.2530).

*Ent*-atisan-16*α*,17-acetonide-19-hydroxy-3-one (Eupfisenoid I, compound 9), white powder; [*α*]24.2 D-20.71 (c 0.14, MeOH); UV (MeOH) *λ*_max_ (log ϵ) 203.5 (3.98) nm; IR (KBr) *ν*_max_ 3,426, 2,929, 2,868, 1,734, 1,451, 1,370, 1,252, 1,057, 724 cm^−1^; ^1^H and ^13^C NMR, see **Tables 2**, **3**; HR-ESI-MS peak at *m*/*z* 399.2510 [M+Na]^+^ (calcd. C_23_H_36_NaO_4_, 399.2506).

### 3.7 X-ray crystallographic data for compound 1

Colorless crystals of 1 were obtained by recrystallization in MeOH at room temperature. X-ray crystal data were acquired on a Bruker APEX-II CCD detector with graphite monochromated Cu Kα radiation (λ = 1.541, 78 Å). The structure of 1 was directly elucidated using SHELXL-97 (Sheldrick 2008) and refined by the full-matrix least-squares difference Fourier method. The x-ray data of 1 have been deposited at the Cambridge Crystallographic Data Center.

### 3.8 Assays of cytopathic effect of compound 1

Vero E6 cells were seeded in 96-well plates and grown overnight. Cells were incubated with SARS-CoV-2 at 37°C for 2 h and infected at a multiplicity of infection of 0.1. Then, the cells were incubated with the maintenance medium in compound **1** (1 mg, 20 μM). Remdesivir (4 μM) was used as positive controls and DMSO solution was used as naive control. After 72 h, cell viability was then assessed using colorimetric MTS assays (Promega Corp.) as described by the manufacturer. Thereafter, the cells were photographed using a microscope.

### 3.9 Molecular docking of compound 1

Autodock tool 1.5.6 software was used to perform operations such as acceptor polarization of hydrogen, Gasteiger charge distribution, and removal of water molecules for 7BV1 and small-molecule ligands. Set the docking central coordinates of the Autodock Vina software to center_x = 131.622, center_y = 135.777, center_z = 121.114. The docking box size is 126 Å, the exhaustiveness value of the search parameter is 10, the top nine conformations are output according to the docking score, and the default value is selected for the rest of the parameters. Finally, the docking results were visualized by PyMOL software.

### 3.10 Microscale thermophoresis of compound 1

Purified SARS-CoV-2 RdRp protein was subjected to NHS (lysine labeling method) labeling. One hundred microliters of 10 µM protein and lysine labeling reagent was incubated in a dark environment for 30 min. The initial concentration of compound 1 (1 mg) was set at 20 mM, and it was subsequently diluted to a concentration of 100 µM using PBS-T buffer, with 16 gradient dilutions of 100 µM. Twenty microliters of compound 1 solution was added to PCR tube 1 and 10 µL of PBS-T buffer was added to the remaining 15 PCR tubes. Ten microliters of solution was pipetted from tube 1 and added to tube 2 and mixed; this process was repeated for tubes 3 to 16, the serial dilution was completed sequentially, and 10 μL was discarded in the last tube. Ten microliters of SARS-CoV-2 RdRp protein solution was added to each PCR tube and mixed well, a capillary was used to aspirate the sample, and the MST experiment was performed. The instrument was set to a medium MST power, and the *K*_d_ values and binding curves of compound 1 and the SARS-CoV-2 RdRp protein were ultimately obtained. The dilution and assay steps were repeated on two separate occasions.

## 4 Discussion

Plants, with their complex secondary metabolism, produce a wide range of compounds and offer significant advantages in the treatment of infectious diseases. Throughout history, phytotherapy has been utilized during epidemics such as the Black Death, smallpox, tuberculosis, malaria, and Spanish flu, providing valuable references for mankind on the safety and effectiveness of plant-based treatments (Garcia, 2020). Developing anti-SARS-CoV-2 drugs based on existing antiviral plants that have a proven track record could streamline the clinical trial process and expedite the identification of potential plant inhibitors (Pandey et al., 2020). In this study, we focused on extracting and isolating compounds from the roots of *E. fischeriana* Steud with potential activity against SARS-CoV-2. Nine undescribed *ent*-atisane type diterpenoids were successfully isolated from this plant. The elucidation of their configurations was achieved through a comprehensive suite of 1D and 2D NMR spectroscopic analyses as well as x-ray diffraction. Atisane-type diterpenoids belong to the tetracyclic diterpenoid family. They possess a bicyclo[2.2.2]octane ring system, decorated with methyl groups at C-4, C-10, and C-16. The most frequently oxidized positions of the *ent*-atisane skeleton are C-3, C-16, and C-17; C-16 and C-17 are typically in the form of an olefin (Drummond et al., 2021). The double bond at C-16 and C-17 is oxidized to a terthydroxyl group at C-16, which undergoes further oxidation to form an acetone dimethyl acetal to finally form the compound we obtained in this study. Many of the ent-atisane diterpenoids have antiviral activity, including anti-influenza A virus (Zhang et al., 2024), anti-HIV-1 (Yan et al., 2018), and anti-human rhinovirus 3 (Wang et al., 2018). Notably, in our study, one of these compounds exhibited promising anti-SARS-CoV-2 activity, and cytopathic effect assays confirmed the anti-SARS-CoV-2 activity of compound 1.

Molecular docking plays a crucial role in the search for antiviral compounds within various plant extracts (Pandey et al., 2020). Using this way of thinking, based on existing antiviral plants, scientists have already employed an integrated approach combining network pharmacology analysis, molecular docking, LC-MS analysis, and bioassays to uncover the potential ingredients of Scutellariae radix for SARS-CoV-2 (Liu et al., 2022). In our study, molecular docking predicted that compound 1 has an affinity for RdRp, with a binding energy of −8.0 kcal/mol. Based on the molecular docking results, our study further investigated the affinity between compound 1 and RdRp using MST, and these findings suggest that compound 1 could serve as a potential therapeutic target against SARS-CoV-2 RdRp. The search for potential antiviral agents targeting SARS-CoV-2 RdRp remains a subject of ongoing research. For instance, the polyphenolic compound gossypol could directly block SARS-CoV-2 RdRp, thereby inhibiting SARS-CoV-2 replication in cellular and mice models of infection (Wang et al., 2022). Other compounds like quercetin and procyanidins have also demonstrated promising inhibitory effects on SARS-CoV-2 RdRp through *in vitro* enzyme assays (Jin et al., 2022; Munafò et al., 2022). The diversity of plant-derived compounds is illustrated by the fact that different types of compounds can act on the same targets. Based on our work, compound 1 also appears promising as a SARS-CoV-2 RdRp inhibitor. In addition, structural modifications may further enhance the anti-SARS-CoV-2 activity of compound 1.

Medicinal plants have long been used to treat infectious diseases and have been vital to human society. As our understanding of plant science grows, we will continue to discover new plant-derived drugs. In order to fully utilize the role of medicinal plants, we need to apply modern technology and a great deal of multidisciplinary research. The current study, which is a tiny portion of plant science research, aims to offer some new scientific foundation.

## 5 Conclusions

In conclusion, this study conducted a preliminary exploration of the material basis, active compounds, and related targets of *E. fischeriana* Steud against SARS-CoV-2. Nine previously unreported *ent*-atisane-type diterpenoid compounds were isolated from the roots of *E. fischeriana* Steud, and their activities, targets, and mechanisms against SARS-CoV-2 were investigated. Cell pathology experiments confirmed that *ent*-atisane-type diterpenoid compound 1 exhibited certain anti-SARS-CoV-2 activity. Compound 1 was predicted to bind to RdRp through high-throughput virtual screening. Subsequently, using MST technology, the affinity between compound 1 and RdRp was tested, revealing that compound 1 could form a stable complex with RdRp protein, with a *K*_d_ value of 31.13 μM. RdRp protein was preliminarily identified as the target of compound 1 against SARS-CoV-2. This work expands the research achievements of *E. fischeriana* Steud and its diterpenoid components in the field of antiviral research, providing valuable references for the discovery of potential anti-SARS-CoV-2 targets and mechanisms. Additionally, it enriches the library of antiviral active compounds against SARS-CoV-2 and clarifies the potential of *ent*-atisane-type diterpenoid compounds in antiviral research, offering new insights for COVID-19 drug development.

## Data availability statement

The original contributions presented in the study are included in the article/**Supplementary Material**. Further inquiries can be directed to the corresponding authors.

## Ethics statement

Ethical approval was not required for the studies on animals in accordance with the local legislation and institutional requirements because only commercially available established cell lines were used.

## Author contributions

TR: Conceptualization, Writing – original draft, Methodology. Z-RX: Conceptualization, Methodology, Writing – original draft. Y-WZ: Formal analysis, Writing – review & editing. S-RF: Formal analysis, Writing – review & editing. JR: Formal analysis, Writing – review & editing. QZ: Writing – review & editing, Methodology. X-LS: Writing – review & editing, Formal analysis. S-LW: Writing – review & editing. L-LX: Writing – review & editing. MQ: Writing – review & editing, Methodology. C-XJ: Methodology, Writing – review & editing. X-JH: Writing – review & editing, Funding acquisition, Supervision. D-ZC: Conceptualization, Funding acquisition, Methodology, Software, Writing – original draft.
