# Supplementary material for: Diterpenoids target SARS-CoV-2 RdRp from the roots of Euphorbia fischeriana Steud
Source: Front Plant Sci. 2024 Jul 25;15:1425759. doi: 10.3389/fpls.2024.1425759 (PMC11306077; doi:10.3389/fpls.2024.1425759)
Supplement: Supplementary file 1 [file DataSheet_1.pdf]

Supplementary Material

Diterpenoids Target SARS-CoV-2 RdRp from the Roots of *Euphorbia fischeriana* Steud

Ting Ruan [1, 2, 3, 4,†], Zheng-Rui Xiang [1, 3, 4, †], Yun-Wu Zhang [1, 3, 5], Shi-Rui Fan [1, 3, 4], Juan Ren [1, 5], Qian Zhao[1, 4], Xiao -Long Sun[1, 6], Shi-Li Wu [1, 4], Li-Li Xu [1, 4], Miao Qiao [1, 4], Chen-Xu Jing[7], Xiao-Jiang Hao [1, 2*], and Duo-Zhi Chen[1, 2*]

[1]State Key Laboratory of Phytochemistry and Plant Resources in West China, Kunming Institute of Botany, Chinese Academy of Sciences, Kunming 650201, People's Republic of China.

[2]Yunnan Characteristic Plant Extraction Laboratory, Kunming 650106, China.

[3]Research Unit of Chemical Biology of Natural Anti-Virus Products, Chinese Academy of Medical Sciences， Beijing, China, 100730.

[4]Kunming College of Life Science, University of Chinese Academy of Sciences, Kunming, Yunnan 650204, People's Republic of China.

[5]Department of Chemical Science and Engineering, Yunnan University, Kunming, Yunnan 650091, People's Republic of China.

[6]Institute of International Rivers and Eco-Security, Yunnan University, Kunming, Yunnan 650091, People's Republic of China.

[7]Research Center of Traditional Chinese Medicine, The Affiliated Hospital to Changchun University of Chinese Medicine, Changchun 130021, China.

[†]These authors contributed equally to this work.

[*]**Correspondence:** Tel: 1388801441; Email: chenduozhi@mail.kib.ac.cn; Tel: 13708713386; Email: haoxj@mail.kib.ac.cn.

1. Crystal Data and Structure Refinement for Compound 1

Table SI1. Crystal data and structure refinement for compound **1**

Figure SI1. View of the pack drawing of compound **1**

2. HRESIMS,UV, IR and 1D and 2D NMR Data of Compounds 1−9

Figure SI2. HRESIMS spectrum of compound **1** (CD_3OD)

Figure SI3. UV spectrum of compound **1** (CD_3OD)

Figure SI4. IR spectrum of compound **1** (CD_3OD)

Figure SI5. ^{1}H NMR spectrum of compound **1** ($CDCl_3$)

Figure SI6. ^{13}C and DEPT spectrum of compound **1** ($CDCl_3$)

Figure SI7. HSQC spectrum of compound **1** ($CDCl_3$)

Figure SI8. HMBC spectrum of compound **1** ($CDCl_3$)

Figure SI9. ^{1}H-^{1}H COSY spectrum of compound **1** ($CDCl_3$)

Figure SI10. ROESY spectrum of compound **1** ($CDCl_3$)

Figure SI11. HRESIMS spectrum of compound **2** (CD_3OD)

Figure SI12. UV spectrum of compound **2** (CD_3OD)

Figure SI13. IR spectrum of compound **2** (CD_3OD)

Figure SI14. ^{1}H NMR spectrum of compound **2** (CD_3OD)

Figure SI15. ^{13}C and DEPT spectrum of compound **2** (CD_3OD)

Figure SI16. HSQC spectrum of compound **2** (CD_3OD)

Figure SI17. HMBC spectrum of compound **2** (CD_3OD)

Figure SI18. ^{1}H-^{1}H COSY spectrum of compound **2** (CD_3OD)

Figure SI19. ROESY spectrum of compound **2** (CD_3OD)

Figure SI20. HRESIMS spectrum of compound **3** (CD_3OD)

Figure SI21. UV spectrum of compound **3** (CD_3OD)

Figure SI22. IR spectrum of compound **3** (CD_3OD)

Figure SI23. ^{1}H NMR spectrum of compound **3** ($CDCl_3$)

Figure SI24. ^{13}C and DEPT spectrum of compound **3** ($CDCl_3$)

Figure SI25. HSQC spectrum of compound **3** ($CDCl_3$)

Figure SI26. HMBC spectrum of compound **3** ($CDCl_3$)

Figure SI27. ^{1}H-^{1}H COSY spectrum of compound **3** ($CDCl_3$)

Figure SI28. ROESY spectrum of compound **3** ($CDCl_3$)

Figure SI29. HRESIMS spectrum of compound **4** (CD_3OD)

Figure SI30. UV spectrum of compound **4** (CD_3OD)

Figure SI31. IR spectrum of compound **4** (CD_3OD)

Figure SI32. ^{1} H NMR spectrum of compound **4** ($CDCl_3$)

Figure SI33. ^{13}C and DEPT spectrum of compound **4** ($CDCl_3$)

Figure SI34. HSQC spectrum of compound **4** ($CDCl_3$)

Figure SI35. HMBC spectrum of compound **4** ($CDCl_3$)

Figure SI36. ^{1}H-^{1}H COSY spectrum of compound **4** ($CDCl_3$)

Figure SI37. ROESY spectrum of compound **4** ($CDCl_3$)

Figure SI38. HRESIMS spectrum of compound **5** (CD_3OD)

Figure SI39. UV spectrum of compound **5** (CD_3OD)

Figure SI40. IR spectrum of compound **5** (CD_3OD)

Figure SI41. ^{1} H NMR spectrum of compound **5** ($CDCl_3$)

Figure SI42. ^{13}C and DEPT spectrum of compound **5** ($CDCl_3$)

Figure SI43. HSQC spectrum of compound **5** ($CDCl_3$)

Figure SI44. HMBC spectrum of compound **5** ($CDCl_3$)

Figure SI45. ^{1}H-^{1}H COSY spectrum of compound **5** ($CDCl_3$)

Figure SI46. ROESY spectrum of compound **5** ($CDCl_3$)

Figure SI47. HRESIMS spectrum of compound **6** (CD_3OD)

Figure SI48. UV spectrum of compound **6** (CD_3OD)

Figure SI49. IR spectrum of compound **6** (CD_3OD)

Figure SI50. ^{1} H NMR spectrum of compound **6** ($CDCl_3$)

Figure SI51. ^{13}C and DEPT spectrum of compound **6** ($CDCl_3$)

Figure SI52. HSQC spectrum of compound **6** ($CDCl_3$)

Figure SI53. HMBC spectrum of compound **6** ($CDCl_3$)

Figure SI54. ^{1}H-^{1}H COSY spectrum of compound **6** ($CDCl_3$)

Figure SI55. ROESY spectrum of compound **6** ($CDCl_3$)

Figure SI56. HRESIMS spectrum of compound **7** (CD_3OD)

Figure SI57. UV spectrum of compound **7** (CD_3OD)

Figure SI58. IR spectrum of compound **7** (CD_3OD)

Figure SI59.^{1} H NMR spectrum of compound **7** ($CDCl_3$)

Figure SI60.^{13}C and DEPT spectrum of compound **7** ($CDCl_3$)

Figure SI61. HSQC spectrum of compound **7** ($CDCl_3$)

Figure SI62. HMBC spectrum of compound **7** ($CDCl_3$)

Figure SI63. ^{1}H-^{1}H COSY spectrum of compound **7** ($CDCl_3$)

Figure SI64. ROESY spectrum of compound **7** ($CDCl_3$)

Figure SI65. HRESIMS spectrum of compound **8** (CD_3OD)

Figure SI66. UV spectrum of compound **8** (CD_3OD)

Figure SI67. IR spectrum of compound **8** (CD_3OD)

Figure SI68.^{1} H NMR spectrum of compound **8** ($CDCl_3$)

Figure SI69.^{13}C and DEPT spectrum of compound **8** ($CDCl_3$)

Figure SI70. HSQC spectrum of compound **8** ($CDCl_3$)

Figure SI71. HMBC spectrum of compound **8** ($CDCl_3$)

Figure SI72. ^{1}H-^{1}H COSY spectrum of compound **8** ($CDCl_3$)

Figure SI73. ROESY spectrum of compound **8** ($CDCl_3$)

Figure SI74. HRESIMS spectrum of compound **9** (CD_3OD)

Figure SI75. UV spectrum of compound **9** (CD_3OD)

Figure SI76. IR spectrum of compound **9** (CD_3OD)

Figure SI77.^{1} H NMR spectrum of compound **9** ($CDCl_3$)

Figure SI78.^{13}C and DEPT spectrum of compound **9** ($CDCl_3$)

Figure SI79. HSQC spectrum of compound **9** ($CDCl_3$)

Figure SI80. HMBC spectrum of compound **9** ($CDCl_3$)

Figure SI81. ^{1}H-^{1}H COSY spectrum of compound **9** ($CDCl_3$)

Figure SI82. ROESY spectrum of compound **9** ($CDCl_3$)

3. Experimental Section

Figure SI83. MST analysis report of compound **1**

1. Crystal Data and Structure Refinement for Compounds 1

Table SI1 Crystal data and structure refinement for compound **1**

Identification code	compound **1**
Empirical formula	$C_{22} H_{34} O_4$
Formula weight	362.49
Temperature	150(2) K
Wavelength	1.54178 Å
Crystal system	Orthorhombic
Space group	$P2_12_12_1$
Unit cell dimensions	a = 6.19640(10) Å
	b = 12.9706(2) Å
	c = 25.2702(5) Å
Volume	2030.99(6) $Å^3$
Z	4
Density (calculated)	1.185 Mg/m^3
Absorption coefficient	0.633 mm^{-1}
F(000)	792
Crystal size	0.410 x 0.090 x 0.030 mm^3
Theta range for data collection	3.50 to 68.16°.
Index ranges	-5<=h<=7, -15<=k<=15, -30<=l<=30
Reflections collected	15315
Independent reflections	3698 [R(int) = 0.1037]
Completeness to theta = 68.16°	99.7 %
Absorption correction	Semi-empirical from equivalents
Max. and min. transmission	0.98 and 0.80
Refinement method	Full-matrix least-squares on F^2
Data / restraints / parameters	3698 / 0 / 240
Goodness-of-fit on F^2	1.064
Final R indices [I>2sigma(I)]	R1 = 0.0372, wR2 = 0.0834
R indices (all data)	R1 = 0.0588, wR2 = 0.0876
Absolute structure parameter	0.01(11)
Largest diff. peak and hole	0.168 and -0.149 e.$Å^{-3}$

Figure SI1. View of the pack drawing of compound **1**

Displacement ellipsoids are drawn at the 30% probability level.

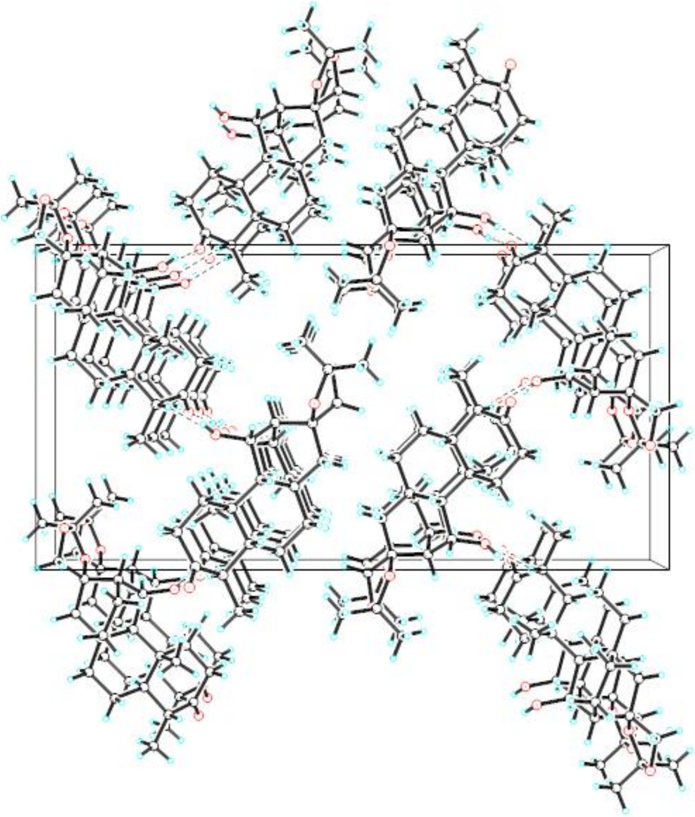

2. HRESIMS,UV, IR and 1D and 2D NMR Data of Compounds 1−9

Figure SI2. HRESIMS spectrum of compound **1**(CD_3OD)

Qualitative Analysis Report

**Data Filename**	HTT-42.d	**Sample Name**	HTT-42
**Sample Type**	Sample	**Position**	P1-F6
**Instrument Name**	Instrument 1	**User Name**	
**Acq Method**	s.m	**Acquired Time**	5/18/2023 1:21:29 PM
**IRM Calibration Status**	Success	**DA Method**	PCDL.m
**Comment**			

Sample Group **Info.**
Acquisition SW Version 6200 series TOF/6500 series Q-TOF B.05.01 (B5125.2)

User Spectra

Fragmentor Voltage	Collision Energy	Ionization Mode
135	0	ESI

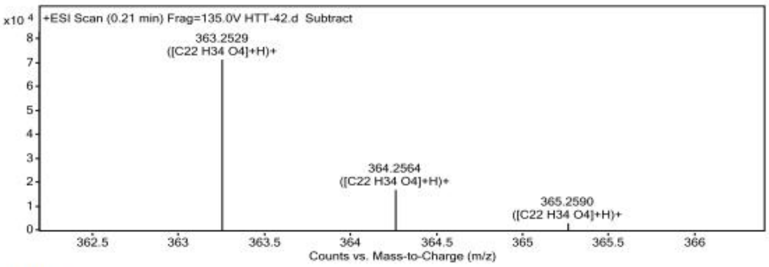

Peak List

m/z	z	Abund	Formula	Ion
287.201	1	9030.54		
363.2529	1	71439.71	C22 H34 O4	(M+H)+
364.2564	1	17095.05	C22 H34 O4	(M+H)+
415.2117	1	113331.93		
416.2151	1	26684.97		
532.2915	1	10427.19		
537.2461	1	74718.01		
538.2492	1	25224.82		
553.2196	1	9995.17		
747.4797	1	10715.79		

Formula Calculator Element Limits

Element	Min	Max
C	3	100
H	0	200
O	0	30

Formula Calculator Results

Formula	CalculatedMass	CalculatedMz	Mz	Diff. (mDa)	Diff. (ppm)	DBE
C22 H34 O4	362.2457	363.2530	363.2529	0.10	0.28	6.0000

--- End Of Report ---

Agilent Technologies Page 1 of 1 Printed at: 1:45 PM on: 5/18/2023

Figure SI3. UV spectrum of compound **1**(CD_3OD)

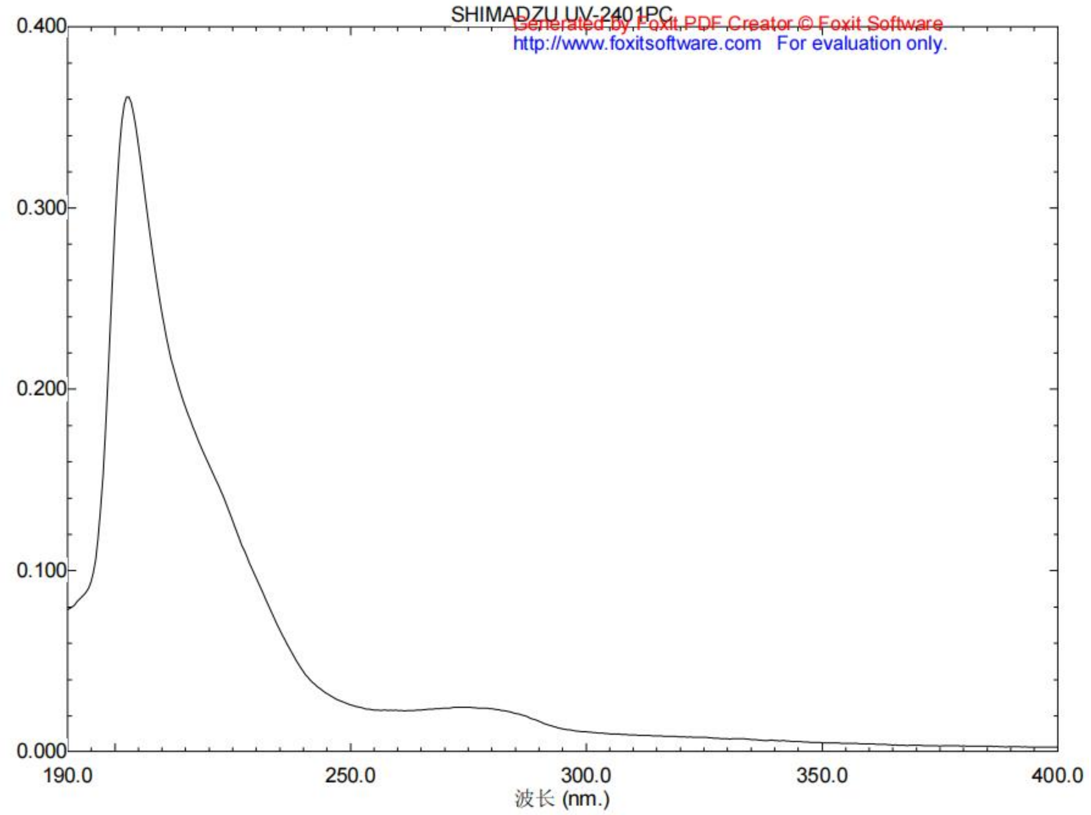

Figure SI4. IR spectrum of compound **1**(CD_3OD)

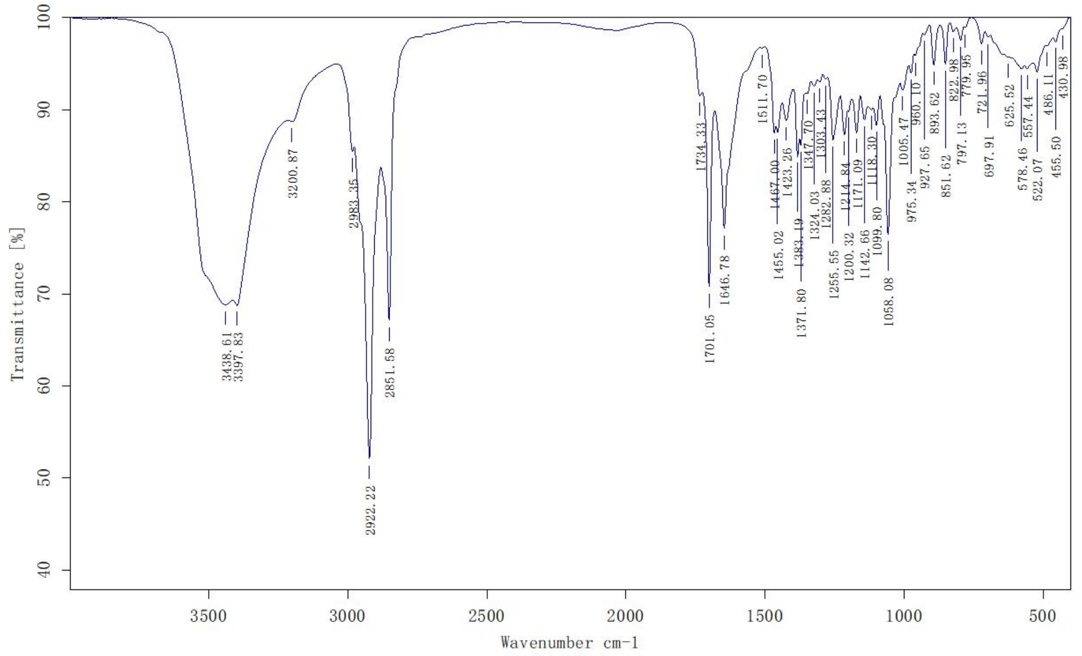

Figure SI5.[1] H NMR spectrum of compound **1** ($CDCl_3$)

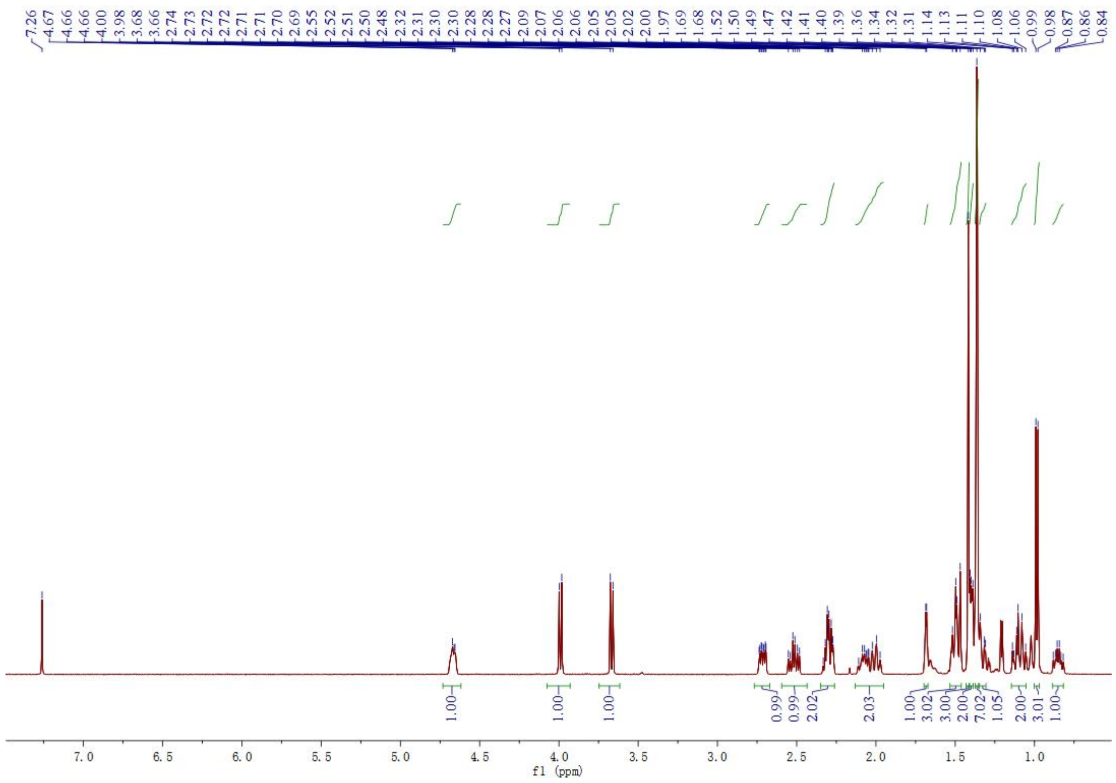

Figure SI6.^{13}C and DEPT spectrum of compound **1** ($CDCl_3$)

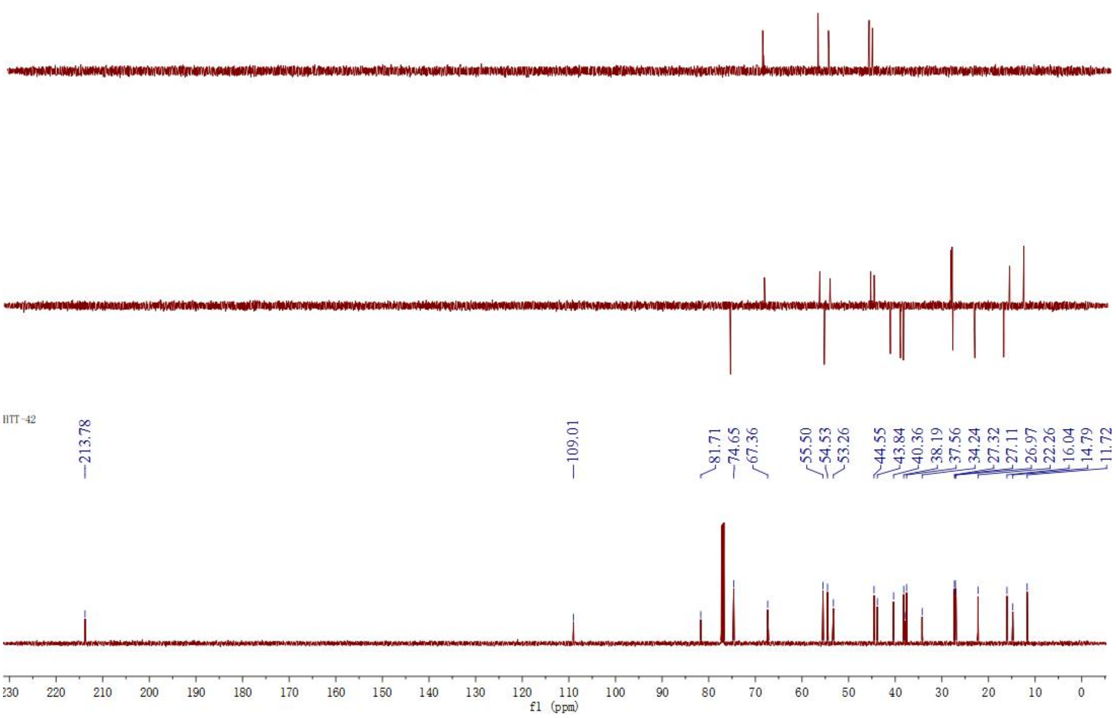

Figure SI7. HSQC spectrum of compound 1 (CDCl3)

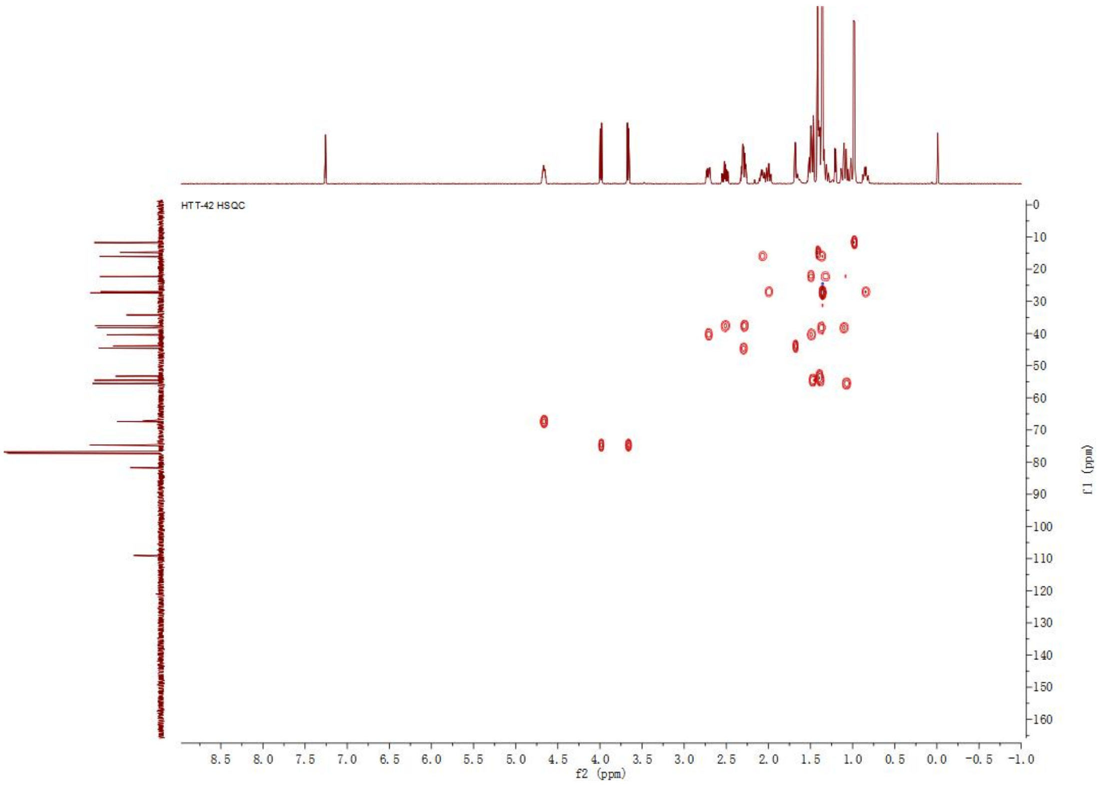

Figure SI8. HMBC spectrum of compound **1** (CDCl3)

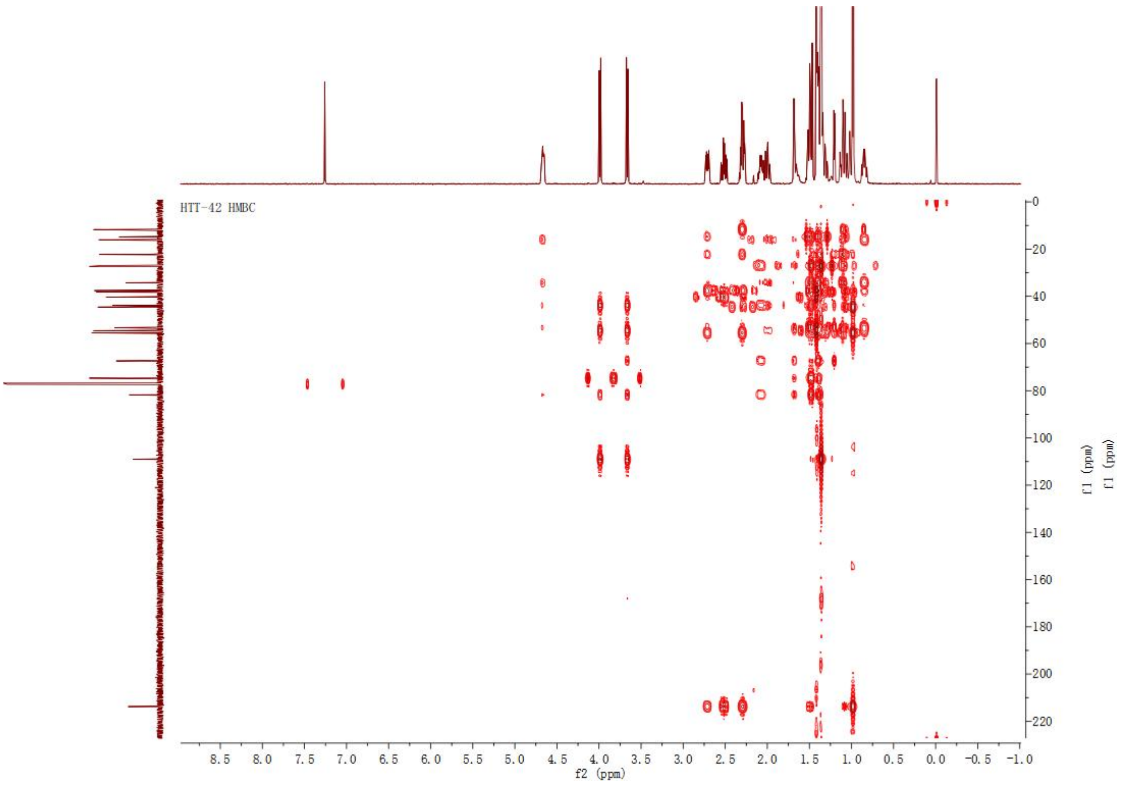

Figure SI9. ^{1}H-^{1}H COSY spectrum of compound **1** ($CDCl_3$)

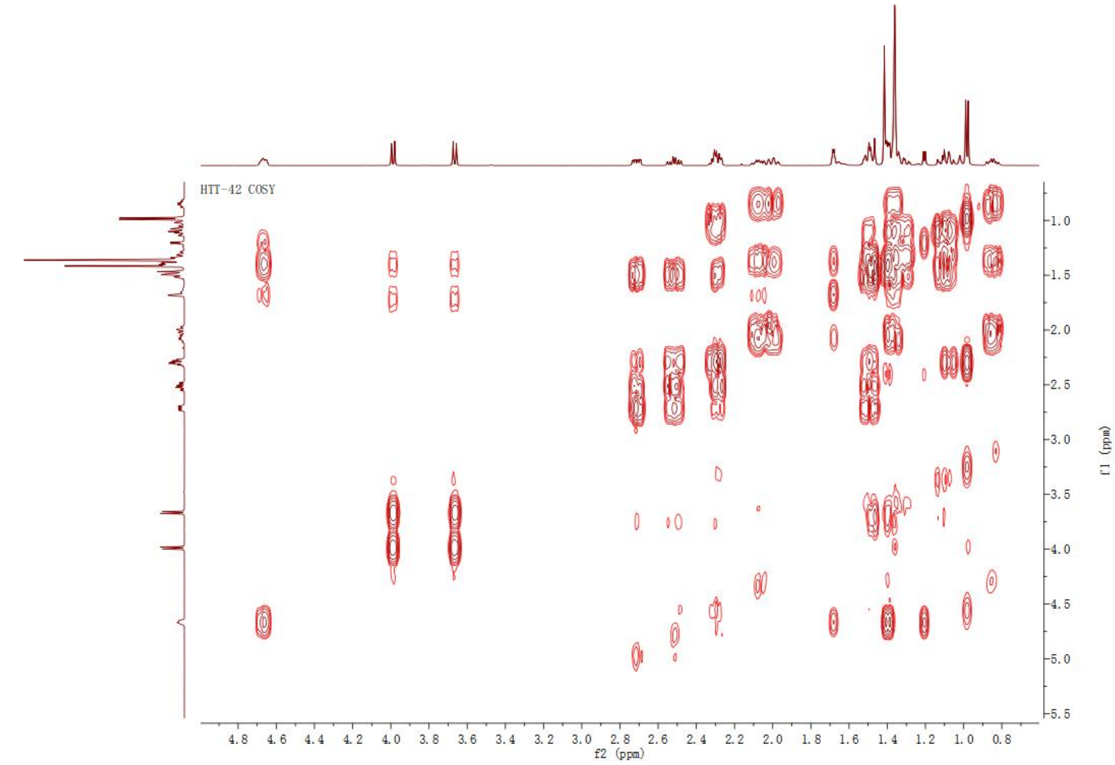

Figure SI10. ROESY spectrum of compound **1** ($CDCl_3$)

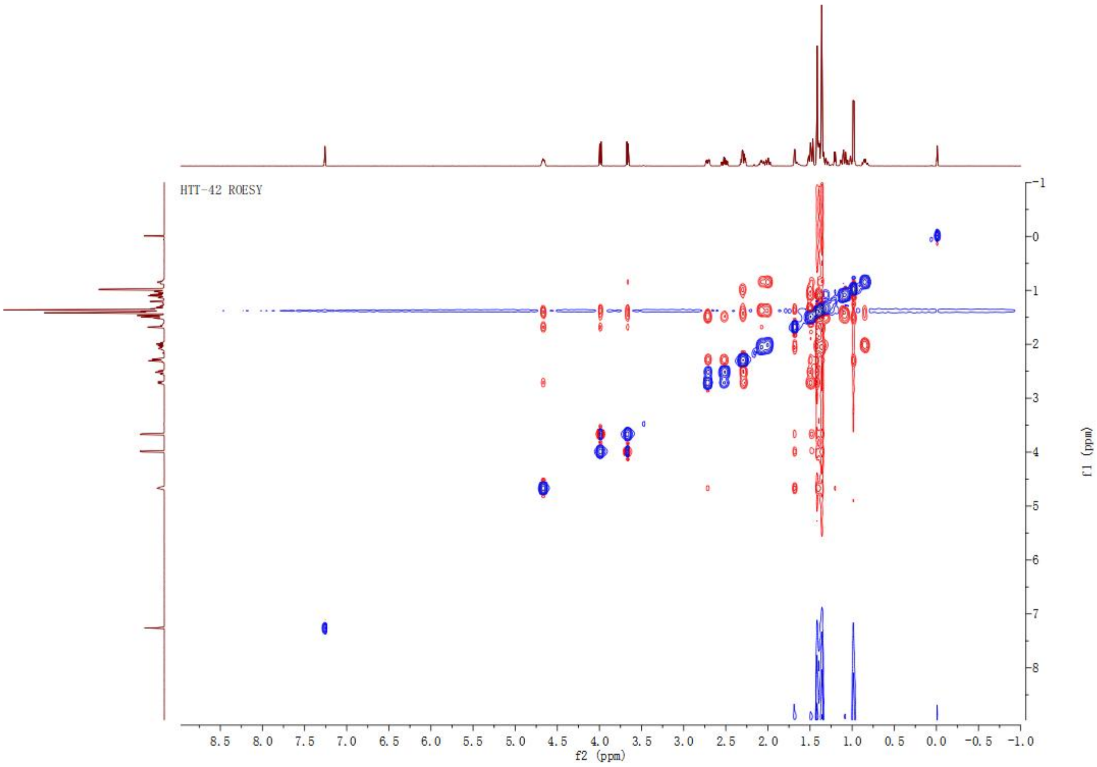

Figure SI11. HRESIMS spectrum of compound 2(CD_3OD)

Qualitative Analysis Report

**Data Filename**	HTT-33.d	**Sample Name**	HTT-33
**Sample Type**	Sample	**Position**	P1-A6
**Instrument Name**	Instrument 1	**User Name**	
**Acq Method**	s.m	**Acquired Time**	4/21/2023 3:50:29 PM
**IRM Calibration Status**	Success	**DA Method**	PCDL.m
**Comment**			

Sample Group **Info.**

Acquisition SW 6200 series TOF/6500 series
Version Q-TOF B.05.01 (B5125.2)

User Spectra

Fragmentor Voltage	Collision Energy	Ionization Mode
135	0	ESI

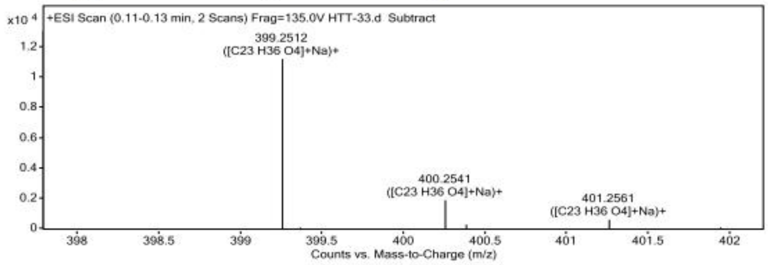

Peak List

m/z	z	Abund	Formula	Ion
269.6521	1	7500.22		
301.2166	1	5432.86		
377.2682	1	6097.77		
399.2512	1	11234.39	C23 H36 O4	(M+Na)+
437.2694	1	6388.81		
437.7709	1	4558.76		
440.2777	1	4693.57		
604.8874	1	4427.13		
775.512	1	27759.19		
776.5145	1	13532.83		

Formula Calculator Element Limits

Element	Min	Max
C	3	60
H	0	120
O	0	30

Formula Calculator Results

Formula	CalculatedMass	CalculatedMz	Mz	Diff. (mDa)	Diff. (ppm)	DBE
C23 H36 O4	376.2614	399.2506	399.2512	-0.60	-1.50	6.0000

--- End Of Report ---

Figure SI12. UV spectrum of compound **2** (CD_3OD)

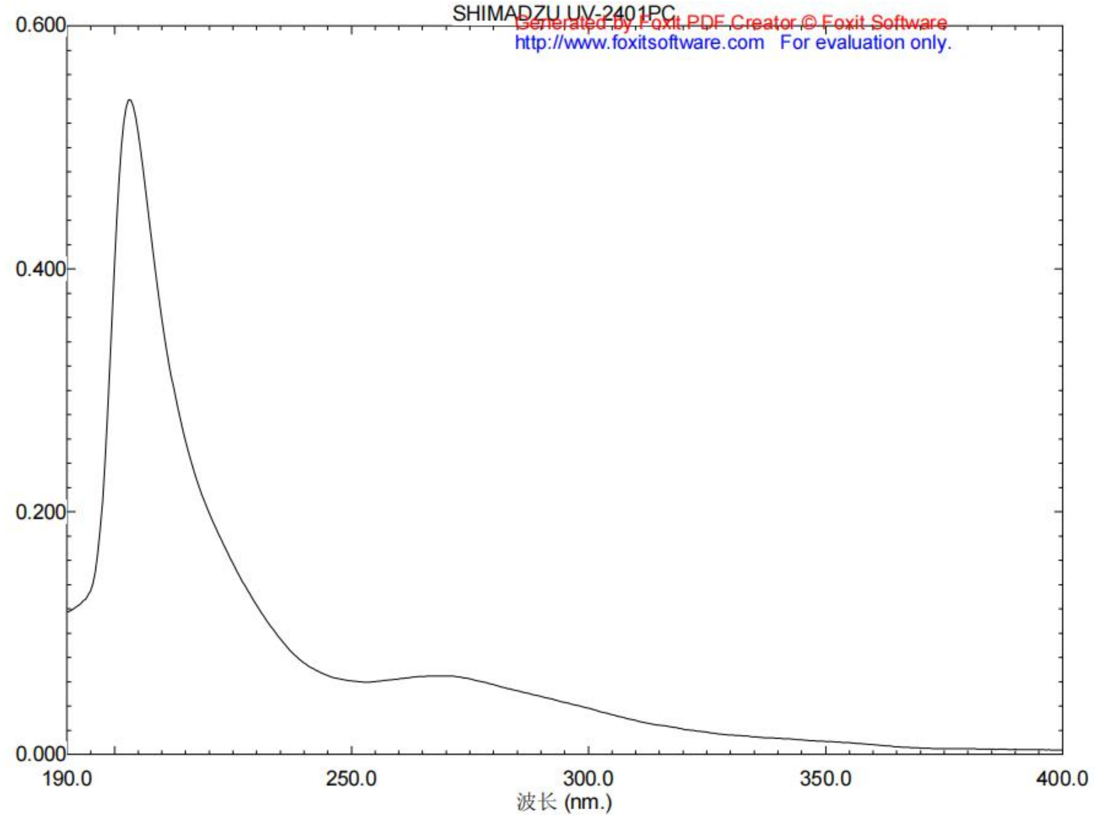

Figure SI13. IR spectrum of compound **2** (CD_3OD)

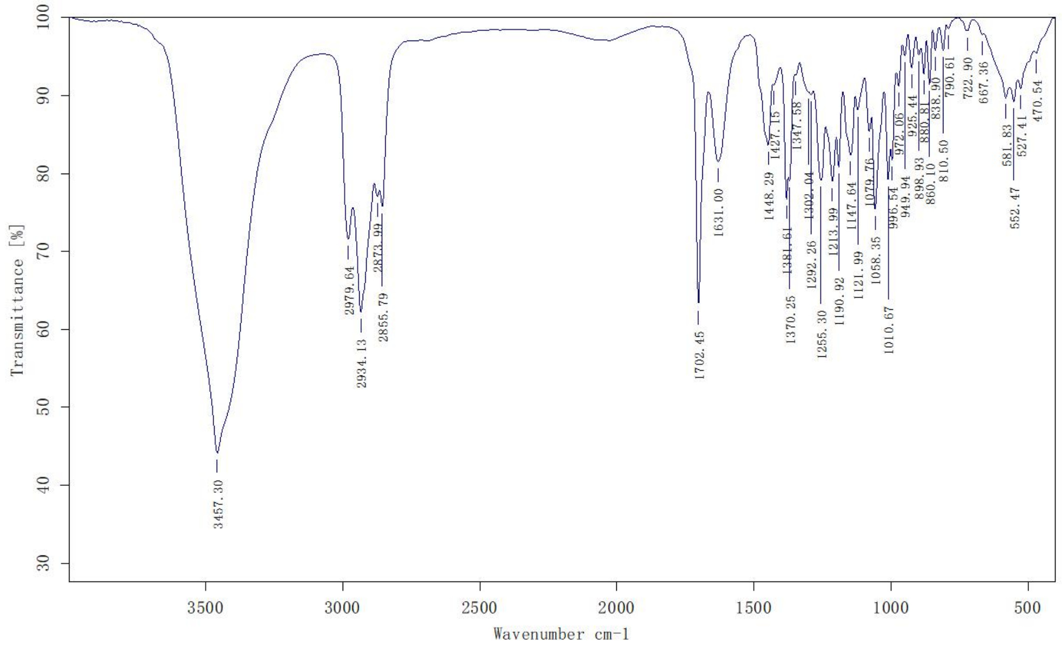

Figure SI14.[1] H NMR spectrum of compound **2** (CD_3OD)

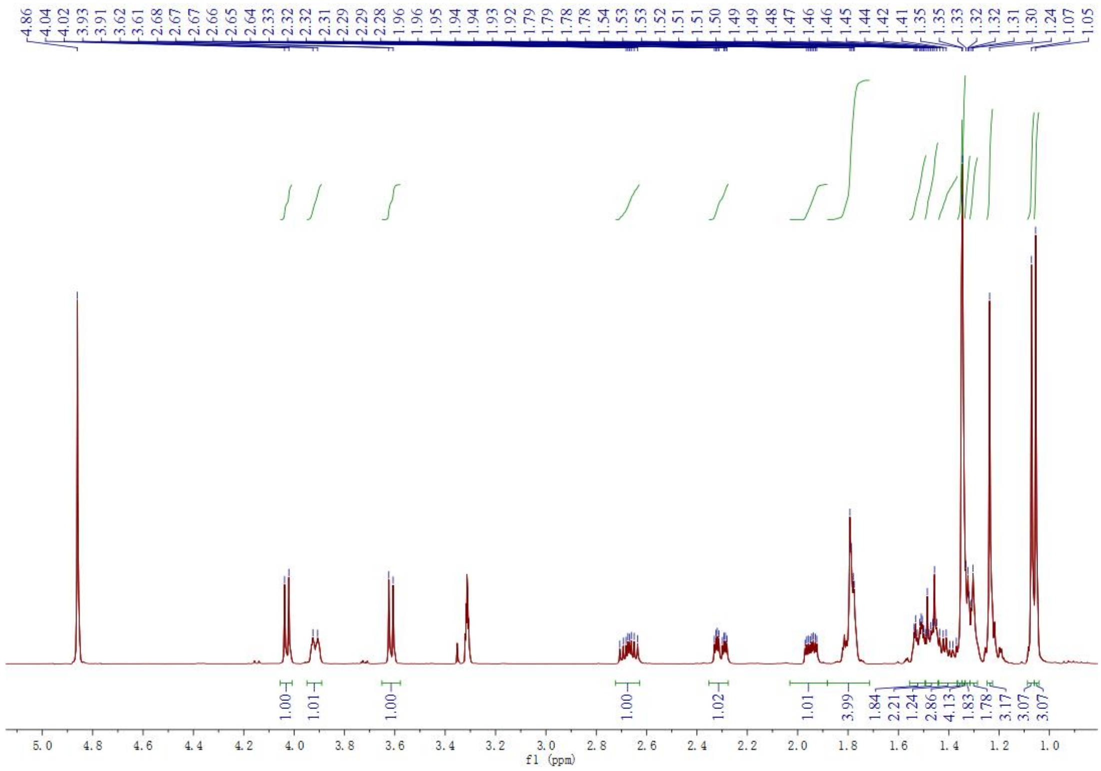

Figure SI15.^{13}C and DEPT spectrum of compound **2** (CD_3OD)

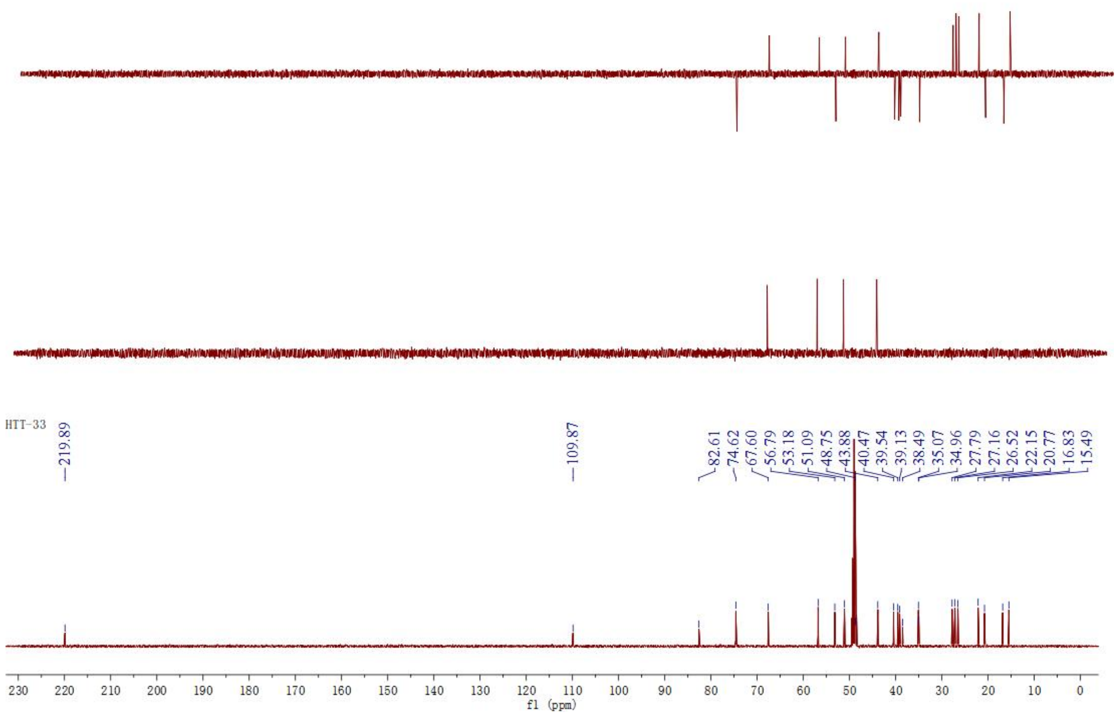

Figure SI16. HSQC spectrum of compound 2 (CD3OD)

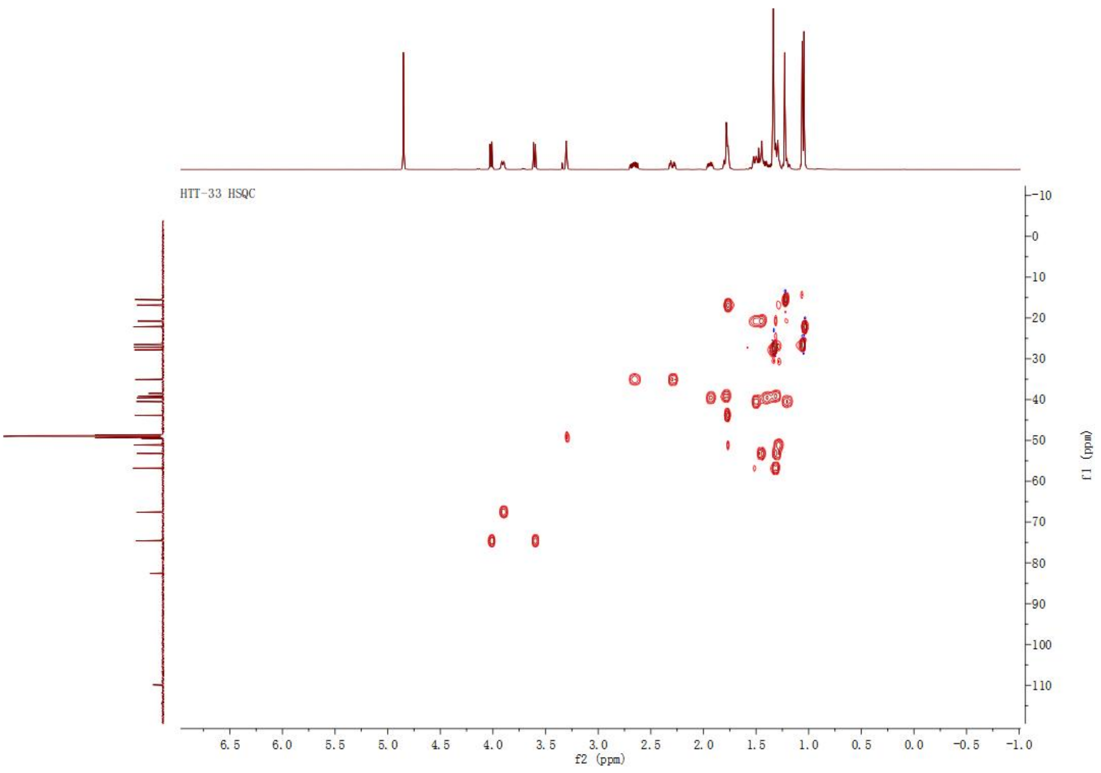

Figure SI17. HMBC spectrum of compound **2** (CD_3OD)

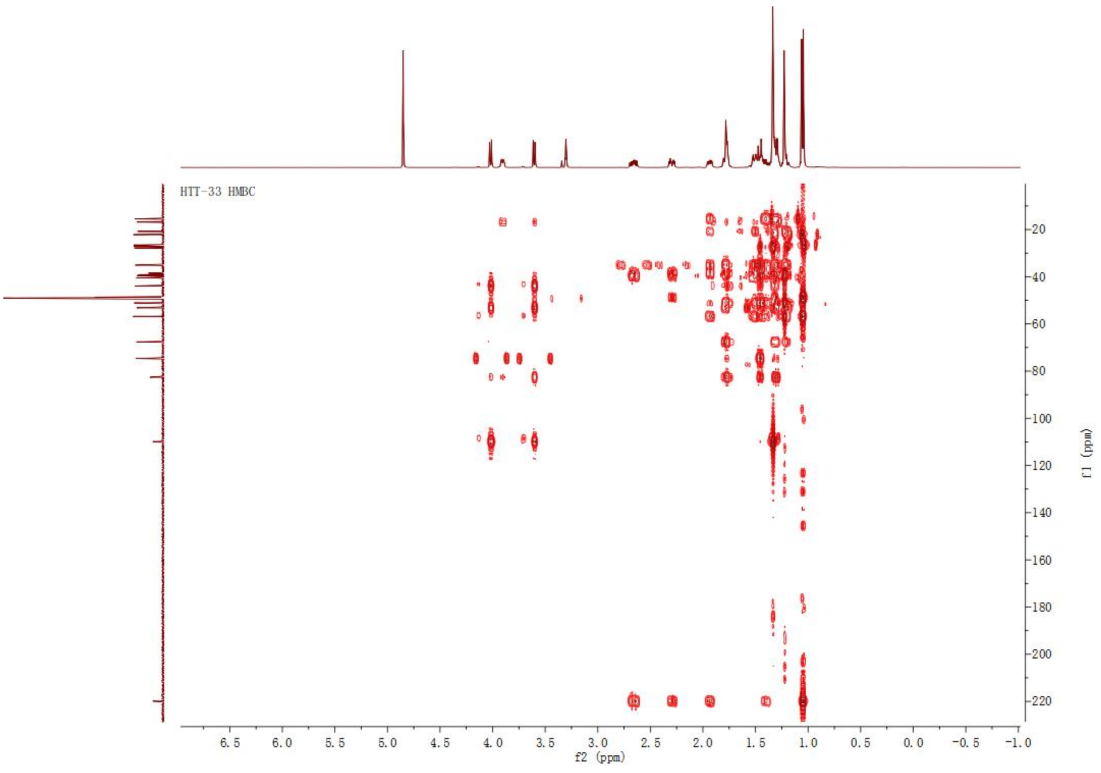

Figure SI18 and SI19 page

Figure SI18. ^{1}H-^{1}H COSY spectrum of compound **2**(CD_3OD)

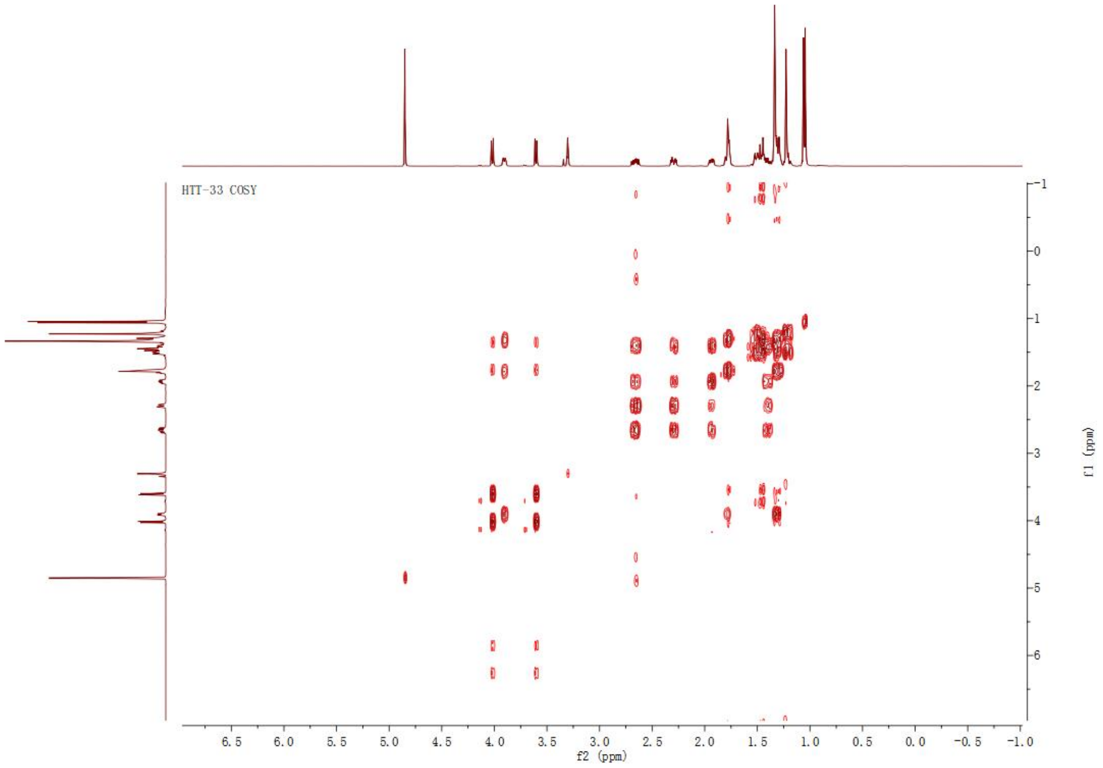

Figure SI19. ROESY spectrum of compound **2** (CD_3OD)

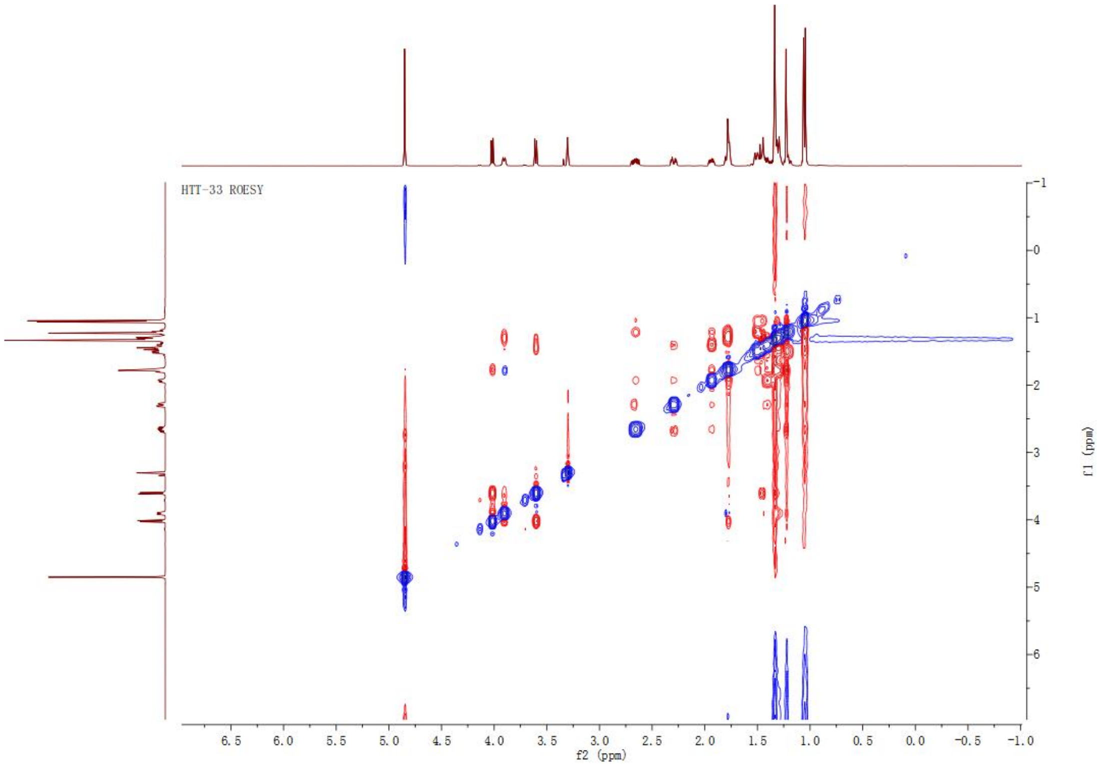

Figure SI20. HRESIMS spectrum of compound **3** (CD_3OD)

Qualitative Analysis Report

**Data Filename**	HRT-20.d	**Sample Name**	HRT-20
**Sample Type**	Sample	**Position**	P1-A5
**Instrument Name**	Instrument 1	**User Name**	
**Acq Method**	s.m	**Acquired Time**	3/15/2023 3:15:34 PM
**IRM Calibration Status**	Success	**DA Method**	PCDL.m
**Comment**			

Sample Group **Info.**

Acquisition SW 6200 series TOF/6500 series

Version Q-TOF B.05.01 (B5125.2)

User Spectra

Fragmentor Voltage	Collision Energy	Ionization Mode
200	0	ESI

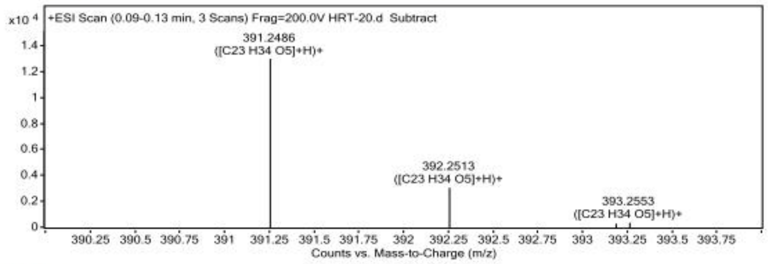

Peak List

m/z	z	Abund	Formula	Ion
297.1855	1	4978.75		
315.196	1	7797.38		
391.2486	1	13029.12	C23 H34 O5	(M+H)+
413.2303	1	5835.81		
781.4904	1	6085.12		
782.4887	1	3275.6		
803.4723	1	11733.2		
804.4754	1	5891.04		
1193.7123	1	3664.86		
1194.7148	1	3244.06		

Formula Calculator Element Limits

Element	Min	Max
C	3	60
H	0	120
O	0	20

Formula Calculator Results

Formula	CalculatedMass	CalculatedMz	Mz	Diff. (mDa)	Diff. (ppm)	DBE
C23 H34 O5	390.2406	391.2479	391.2486	-0.70	-1.79	7.0000

--- End Of Report ---

Agilent Technologies Page 1 of 1 Printed at: 3:18 PM on: 3/15/2023

Figure SI21. UV spectrum of compound **3** (CD_3OD)

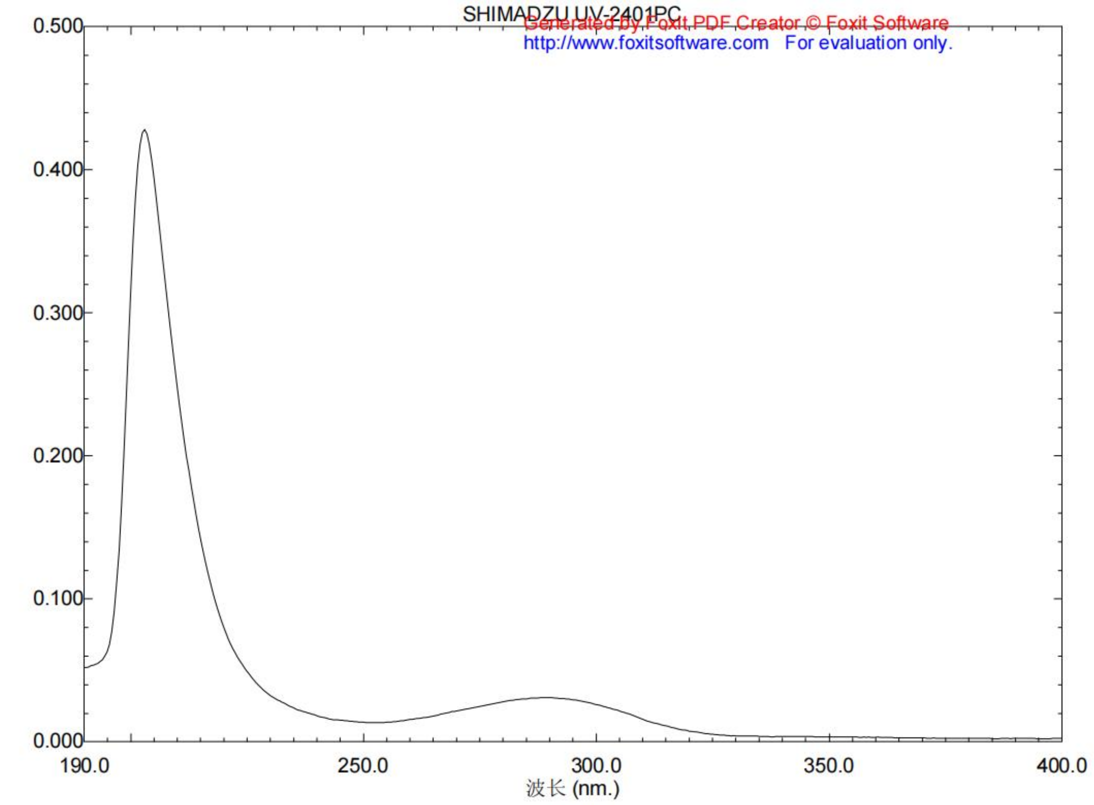

Figure SI22. IR spectrum of compound **3** (CD_3OD)

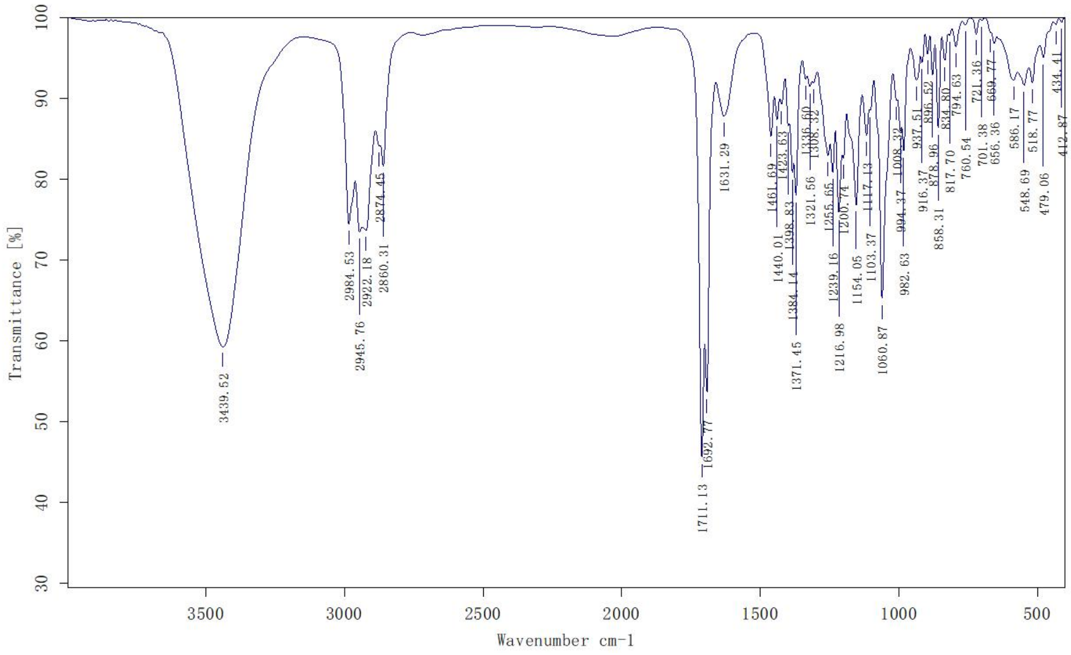

Figure SI23.[1] H NMR spectrum of compound **3** ($CDCl_3$)

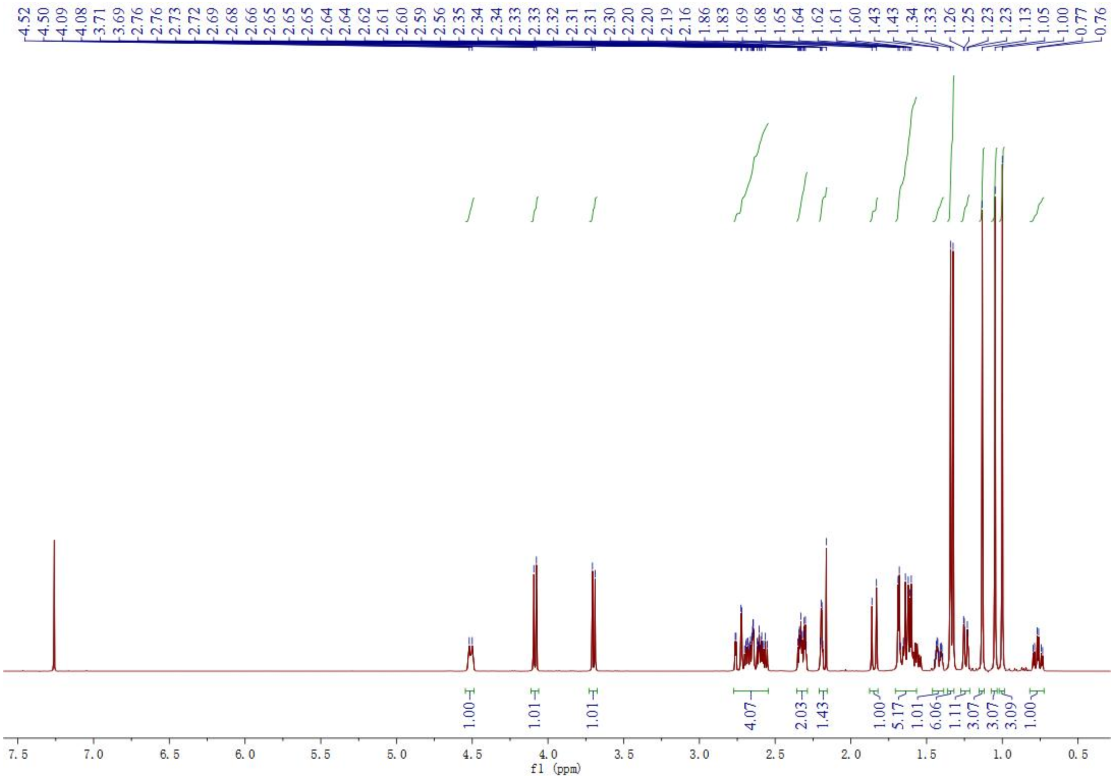

Figure SI24.^{13}C and DEPT spectrum of compound **3** ($CDCl_3$)

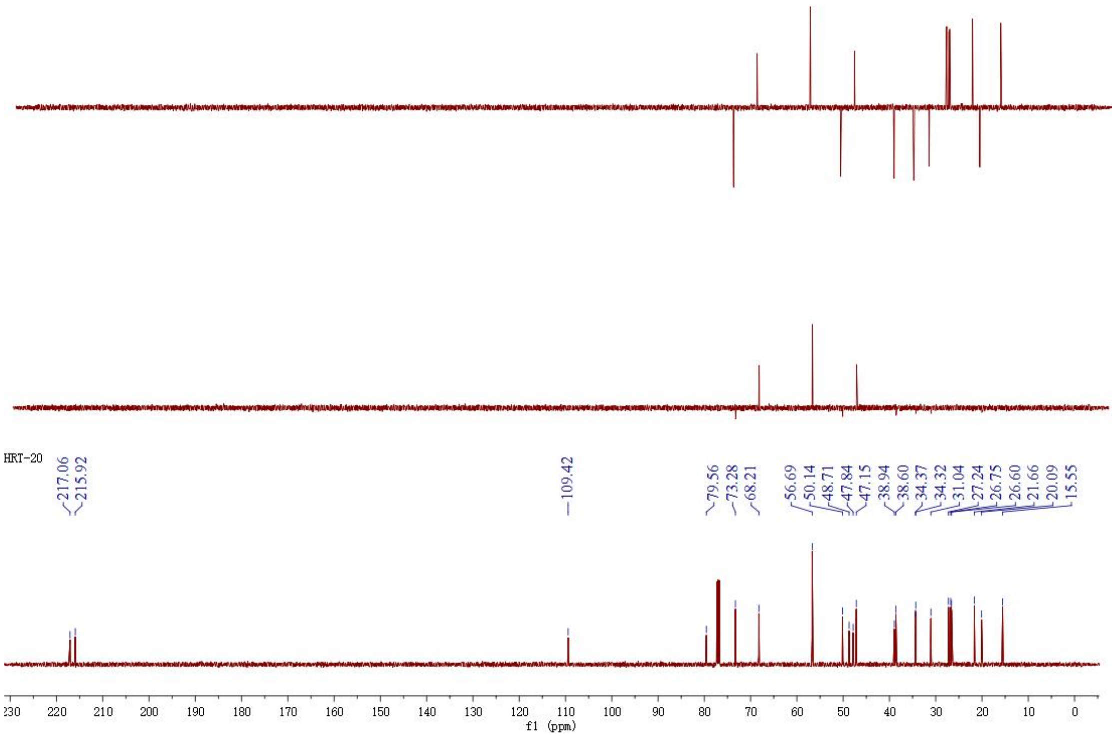

Figure SI25. HSQC spectrum of compound **3** ($CDCl_3$)

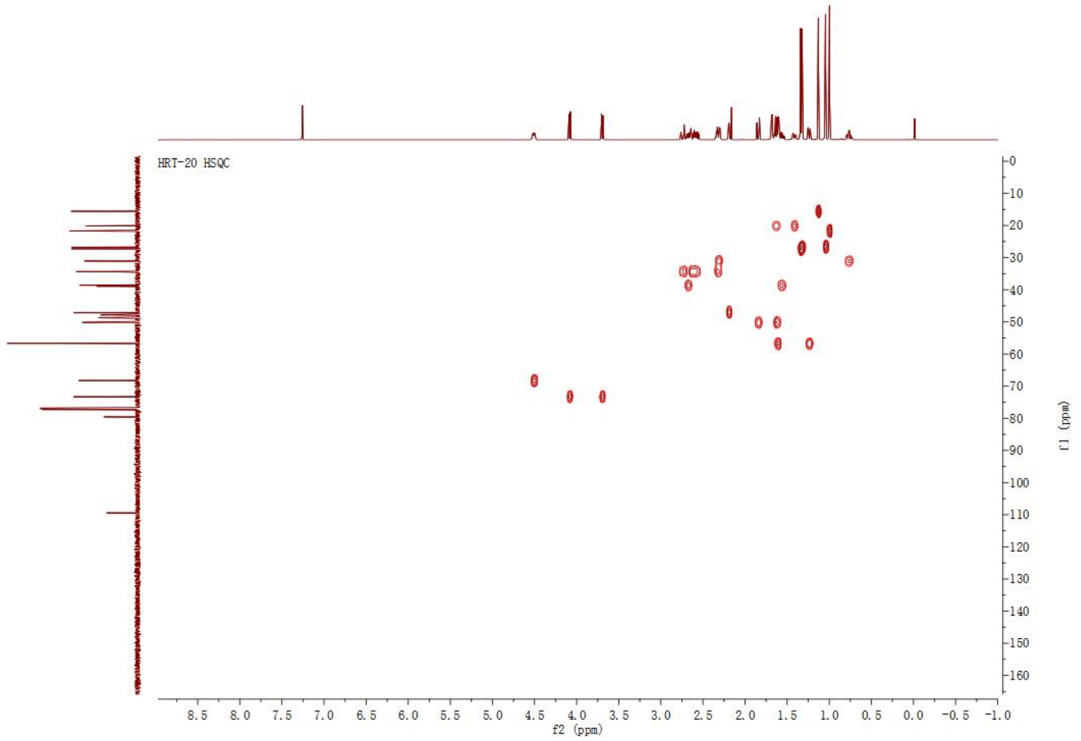

Figure SI26. HMBC spectrum of compound **3** ($CDCl_3$)

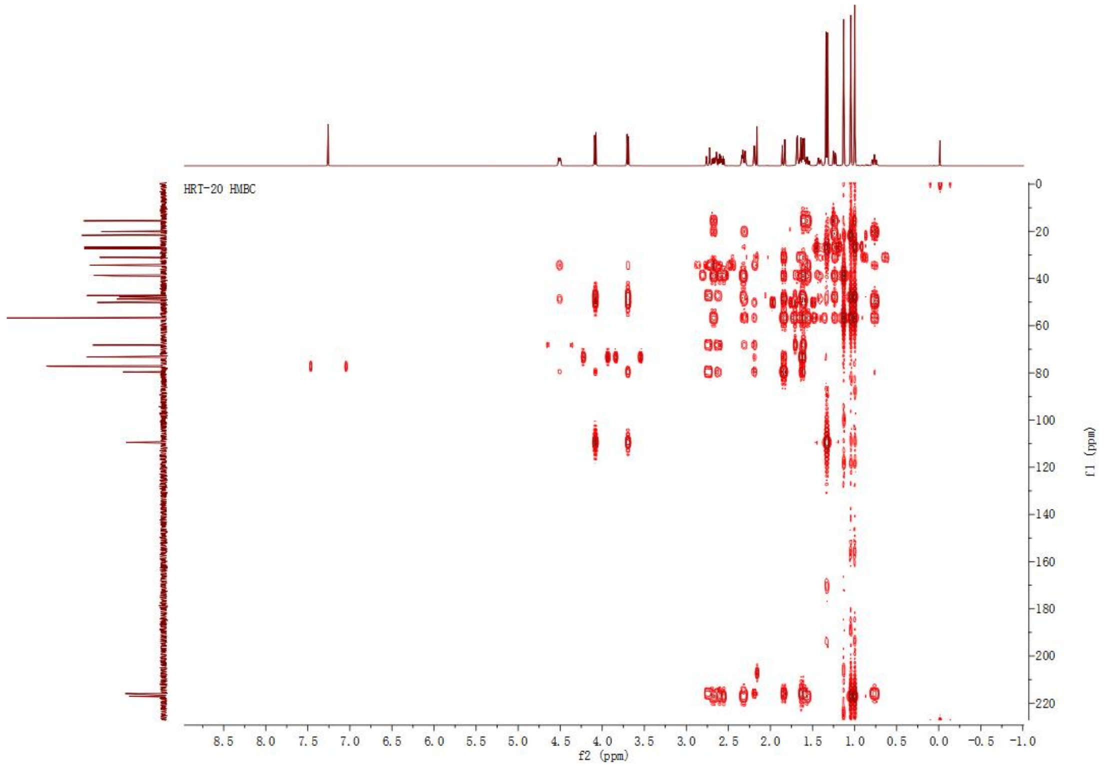

Figure SI27. ^{1}H-^{1}H COSY spectrum of compound **3** ($CDCl_3$)

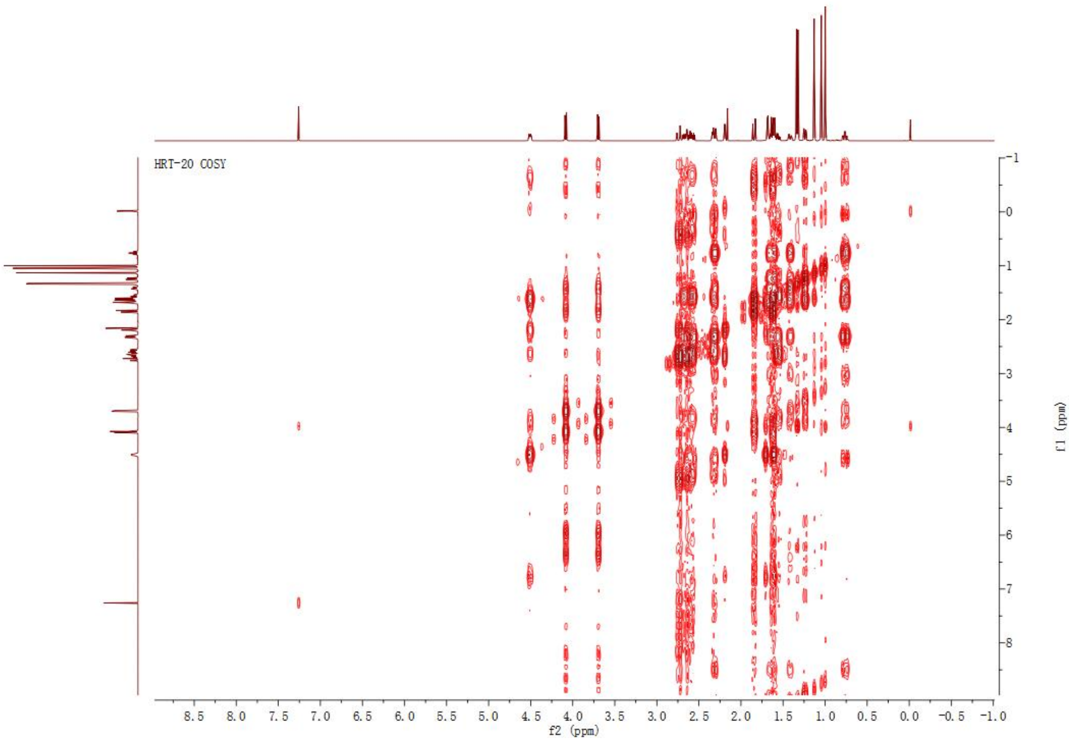

Figure SI28. ROESY spectrum of compound **3** ($CDCl_3$)

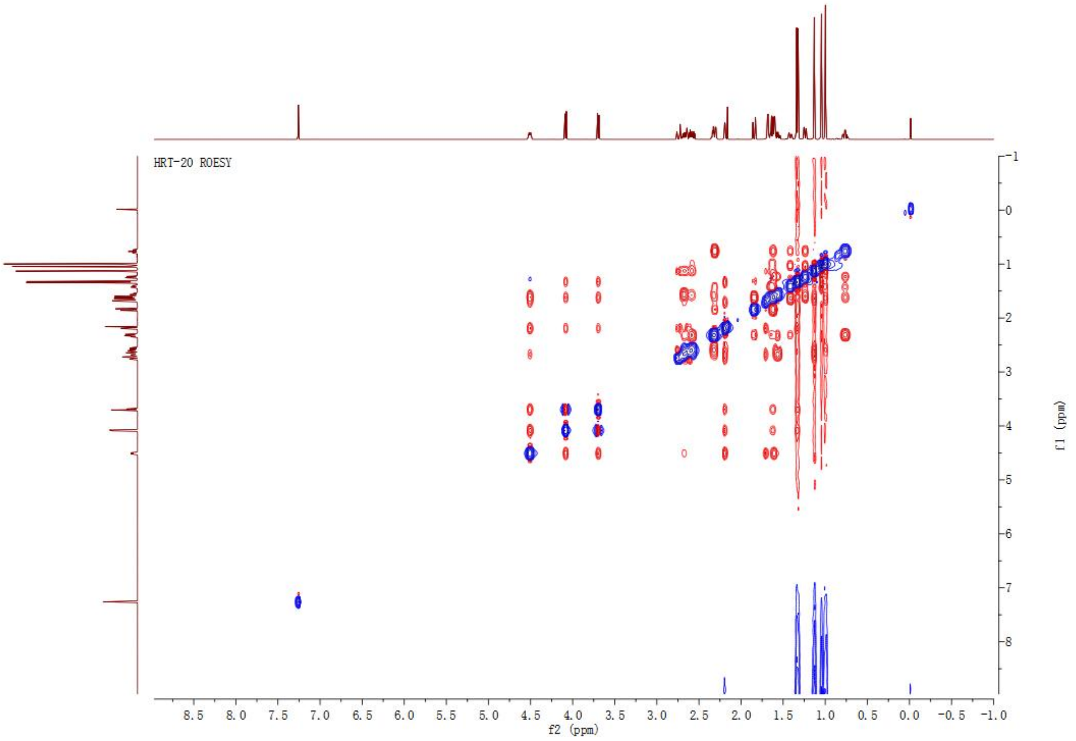

Figure SI29. HRESIMS spectrum of compound **4** (CD_3OD)

Qualitative Analysis Report

**Data Filename**	HTT-57.d	**Sample Name**	HTT-57
**Sample Type**	Sample	**Position**	P1-A1
**Instrument Name**	Instrument 1	**User Name**	
**Acq Method**	s.m	**Acquired Time**	12/19/2023 4:13:41 PM
**IRM Calibration Status**	Success	**DA Method**	PCDL.m
**Comment**			

Sample Group **Info.**
Acquisition SW 6200 series TOF/6500 series
Version Q-TOF B.05.01 (B5125.2)

User Spectra

Fragmentor Voltage	Collision Energy	Ionization Mode
165	30	ESI

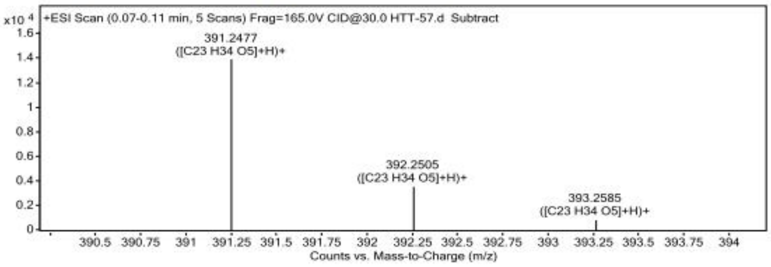

Peak List

m/z	z	Abund	Formula	Ion
287.2003	1	4136.35		
297.1845	1	8963.35		
315.1954	1	10997.95		
373.2368	1	3962.19		
391.2477	1	13953.58	C23 H34 O5	(M+H)+
392.2505	1	3611.78	C23 H34 O5	(M+H)+
413.2298	1	6689.59		
415.2454	1	4672.24		
803.47	1	7734.79		
804.474	1	3797.57		

Formula Calculator Element Limits

Element	Min	Max
C	3	200
H	0	300
O	0	40

Formula Calculator Results

Formula	CalculatedMass	CalculatedMz	Mz	Diff. (mDa)	Diff. (ppm)	DBE
C23 H34 O5	390.2406	391.2479	391.2477	0.20	0.51	7.0000

--- End Of Report ---

Agilent Technologies Page 1 of 1 Printed at: 4:33 PM on: 12/20/2023

Figure SI30. UV spectrum of compound **4** (CD_3OD)

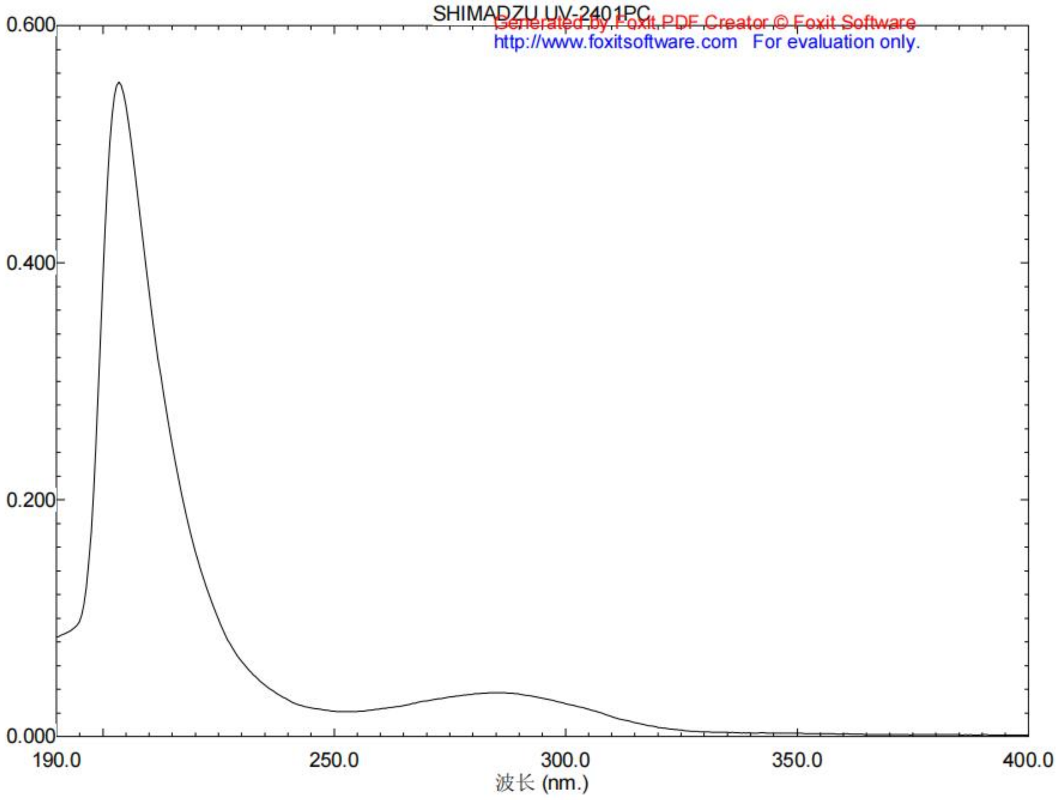

Figure SI31. IR spectrum of compound **4** (CD_3OD)

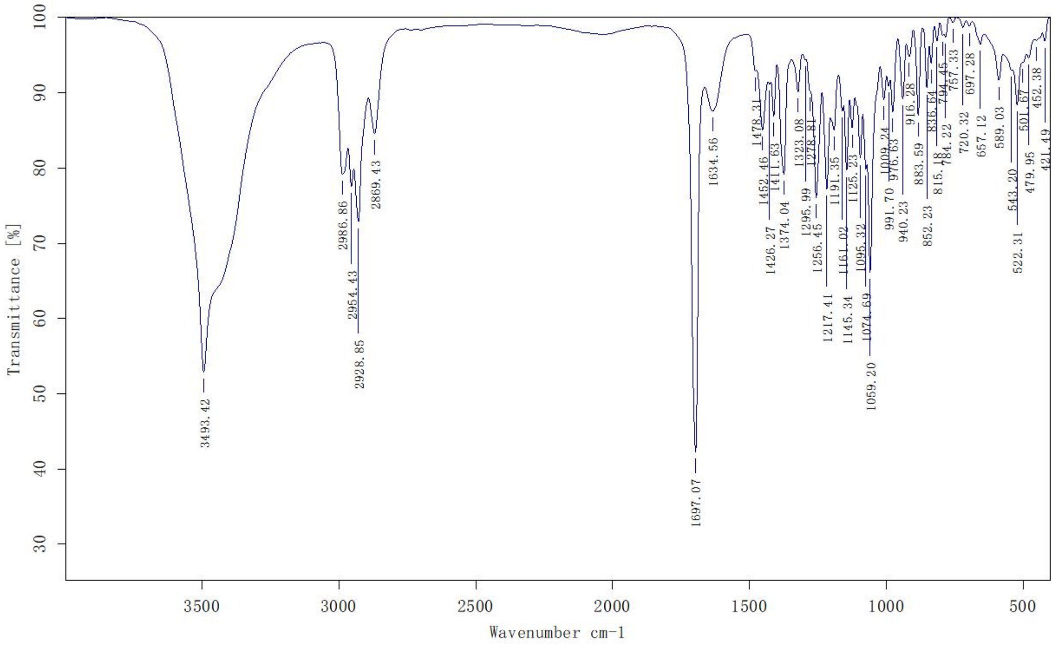

Figure SI32.[1] H NMR spectrum of compound **4** (CDCl3)

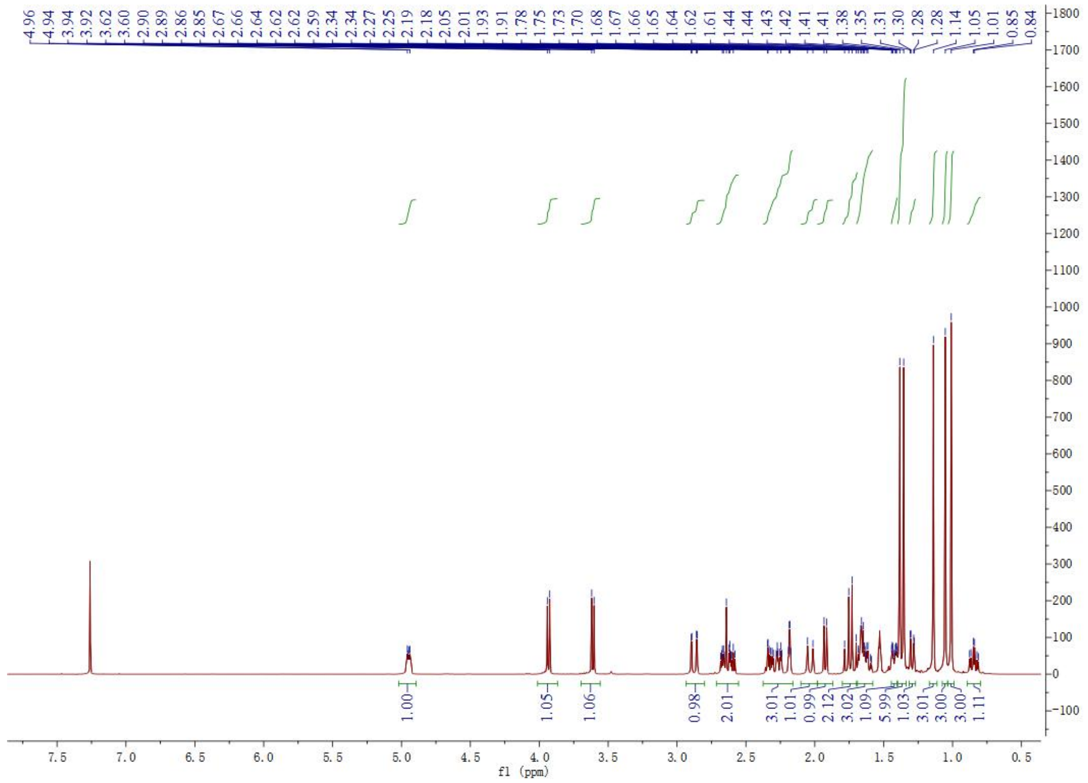

Figure SI33.^{13}C and DEPT spectrum of compound **4** (CDCl3)

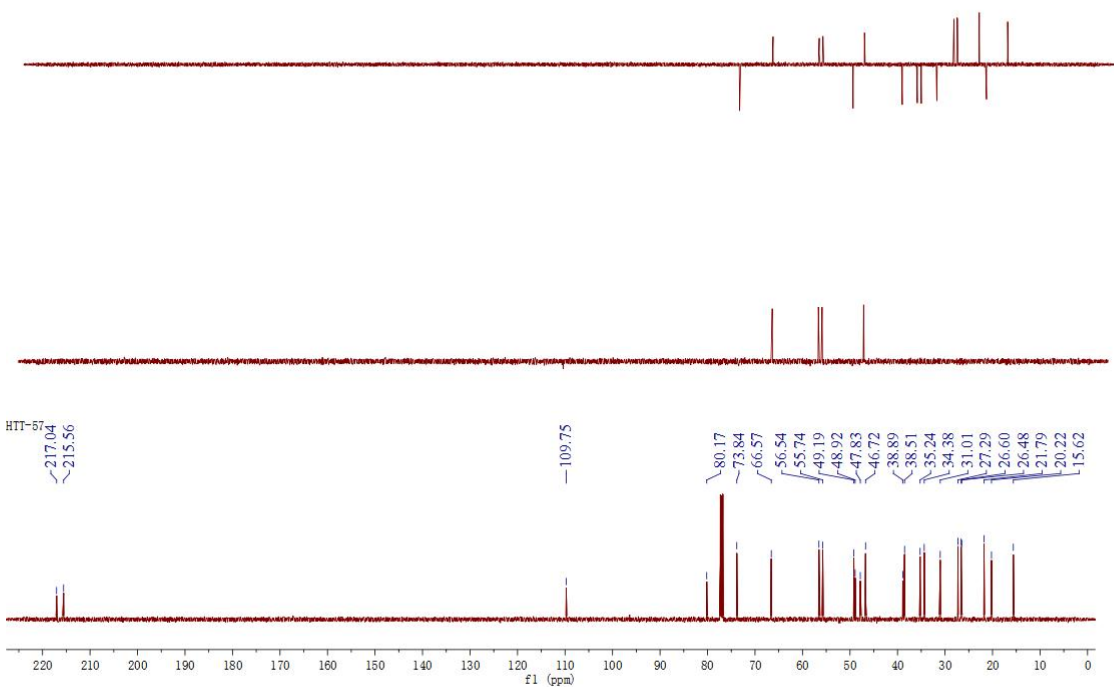

Figure SI34. HSQC spectrum of compound **4** ($CDCl_3$)

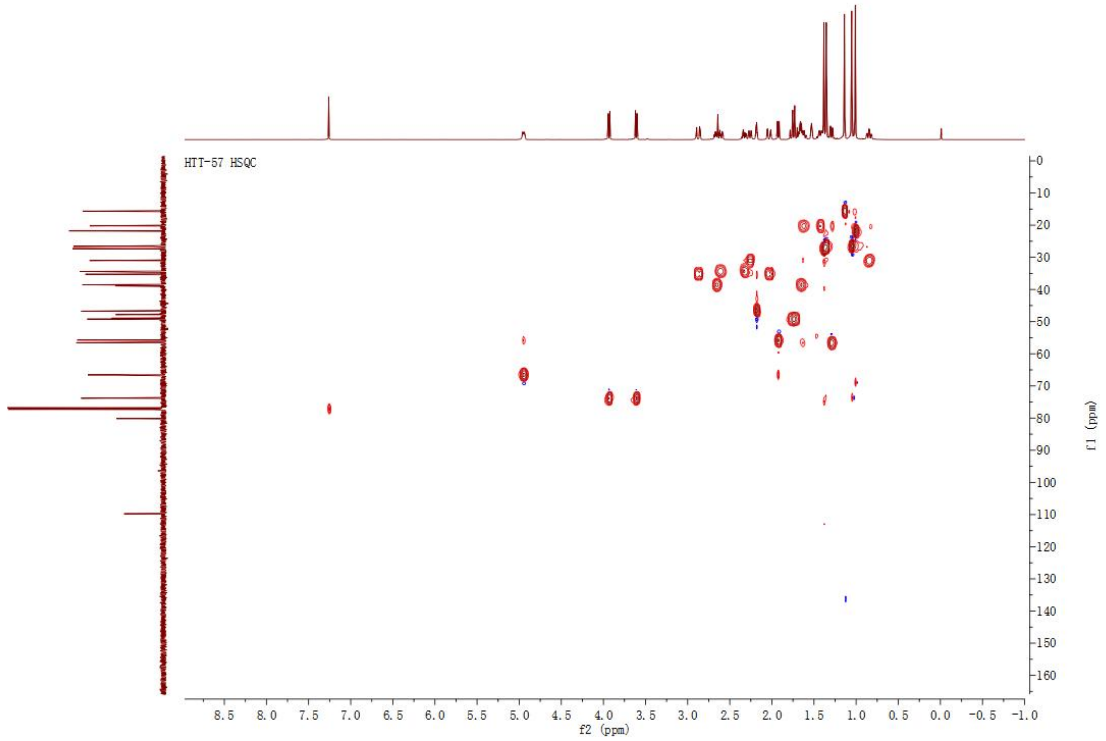

Figure SI35. HMBC spectrum of compound **4** ($CDCl_3$)

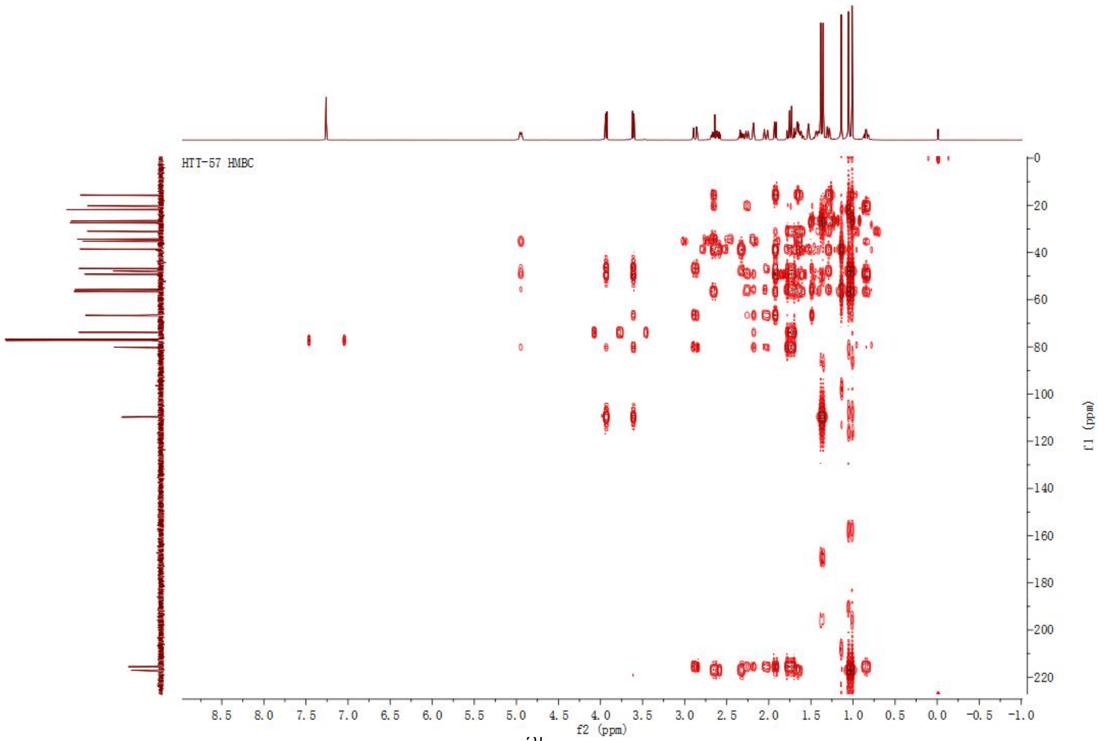

Figure SI36. ^{1}H-^{1}H COSY spectrum of compound **4** ($CDCl_3$)

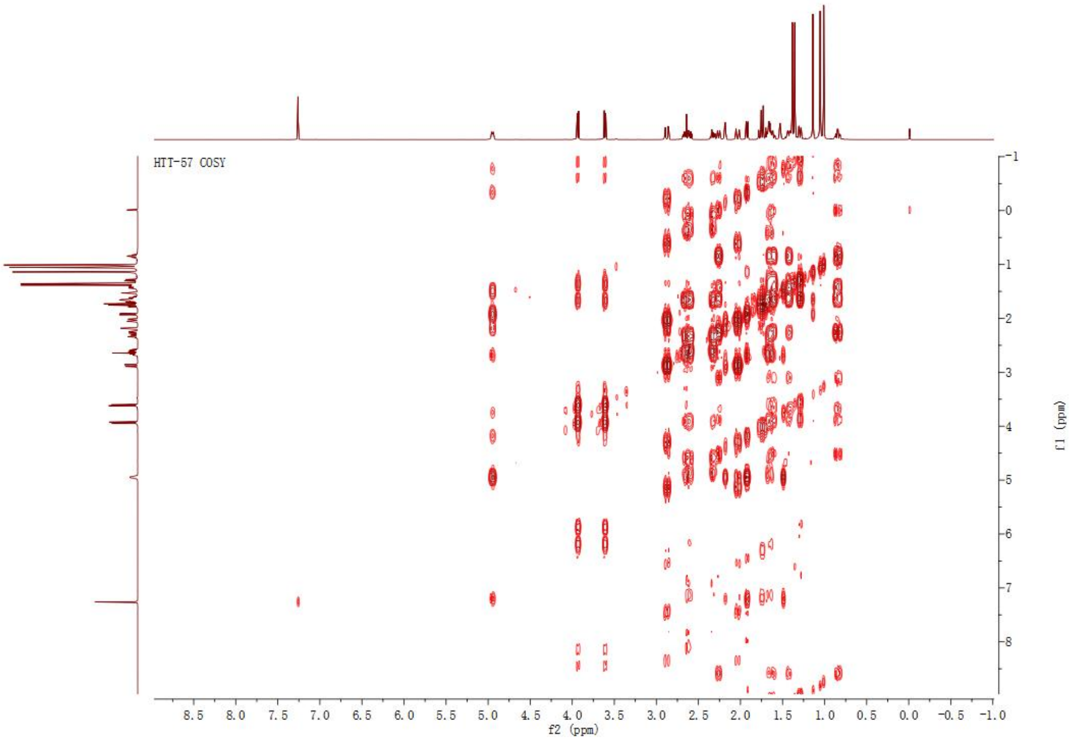

Figure SI37. ROESY spectrum of compound **4** ($CDCl_3$)

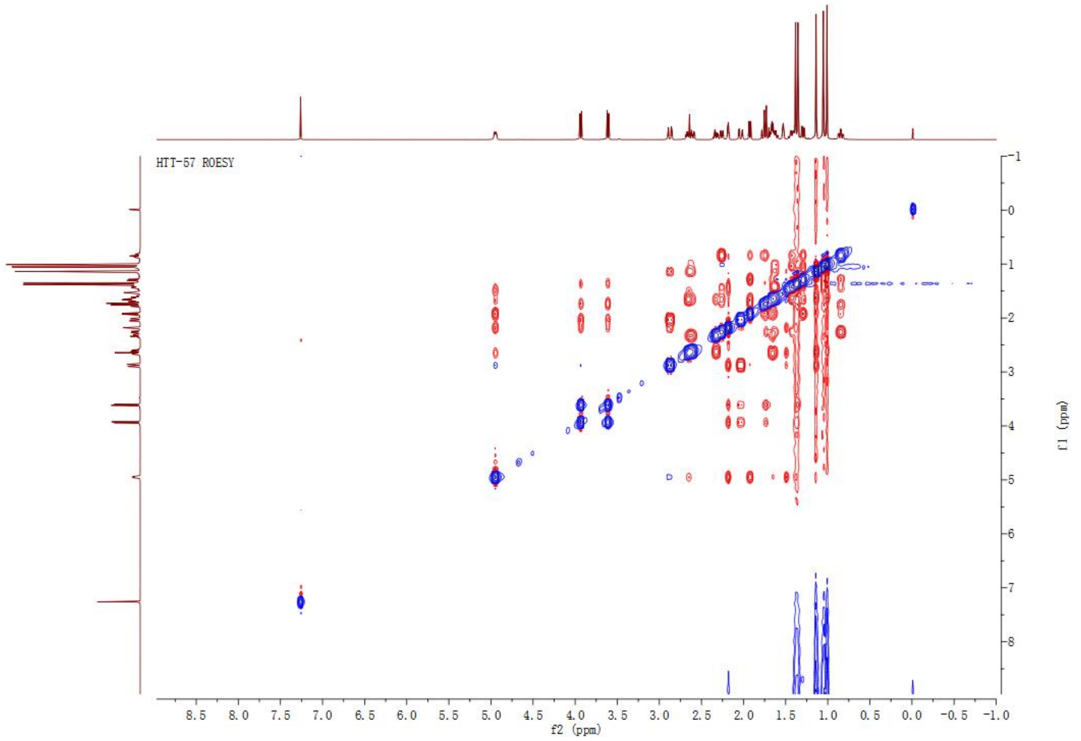

Figure SI38. HRESIMS spectrum of compound **5** (CD_3OD)

Qualitative Analysis Report

**Data Filename**	HTT-54-.d	**Sample Name**	HTT-54
**Sample Type**	Sample	**Position**	P1-B4
**Instrument Name**	Instrument 1	**User Name**	
**Acq Method**	s-.m	**Acquired Time**	7/7/2023 11:52:05 AM
**IRM Calibration Status**	Success	**DA Method**	PCDL.m
**Comment**			

Sample Group **Info.**
Acquisition SW 6200 series TOF/6500 series
Version Q-TOF B.05.01 (B5125.2)

User Spectra

Fragmentor Voltage	Collision Energy	Ionization Mode
135	0	ESI

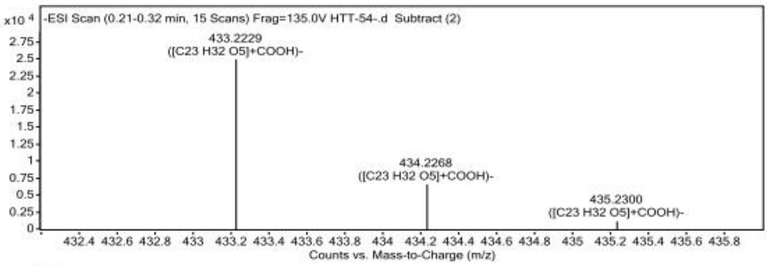

Peak List

m/z	z	Abund	Formula	Ion
61.9884	1	10683.76		
387.2174	1	25593.12		
388.2208	1	6599.78		
423.193	1	9920.97		
424.1977	1	2756.37		
425.1918	1	3404.43		
433.2229	1	25005.76	C23 H32 O5	(M+COOH)-
434.2268	1	6671.07	C23 H32 O5	(M+COOH)-
450.2129	1	8112.49		
1019.9501	1	4837.32		

Formula Calculator Element Limits

Element	Min	Max
C	3	60
H	0	200
O	0	20

Formula Calculator Results

Formula	CalculatedMass	CalculatedMz	Mz	Diff. (mDa)	Diff. (ppm)	DBE
C23 H32 O5	388.2250	433.2232	433.2229	0.30	0.69	8.0000

--- End Of Report ---

Figure SI39. UV spectrum of compound **5** (CD_3OD)

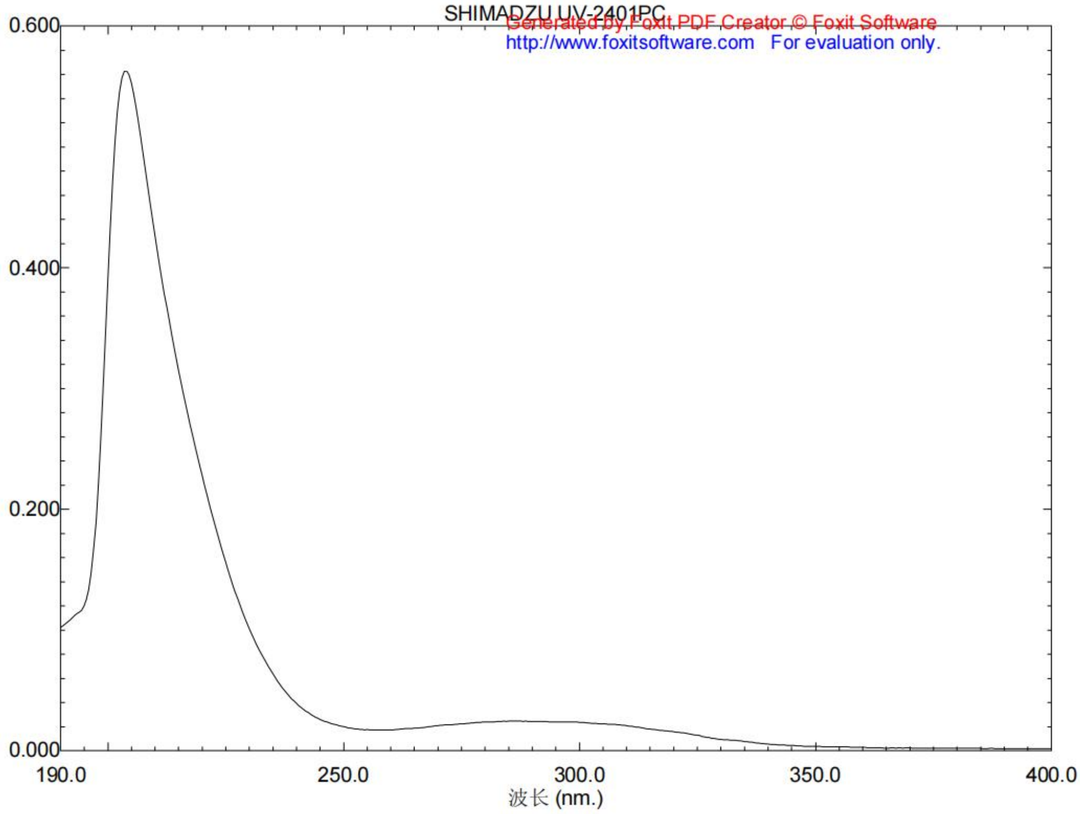

Figure SI40. IR spectrum of compound **5** (CD_3OD)

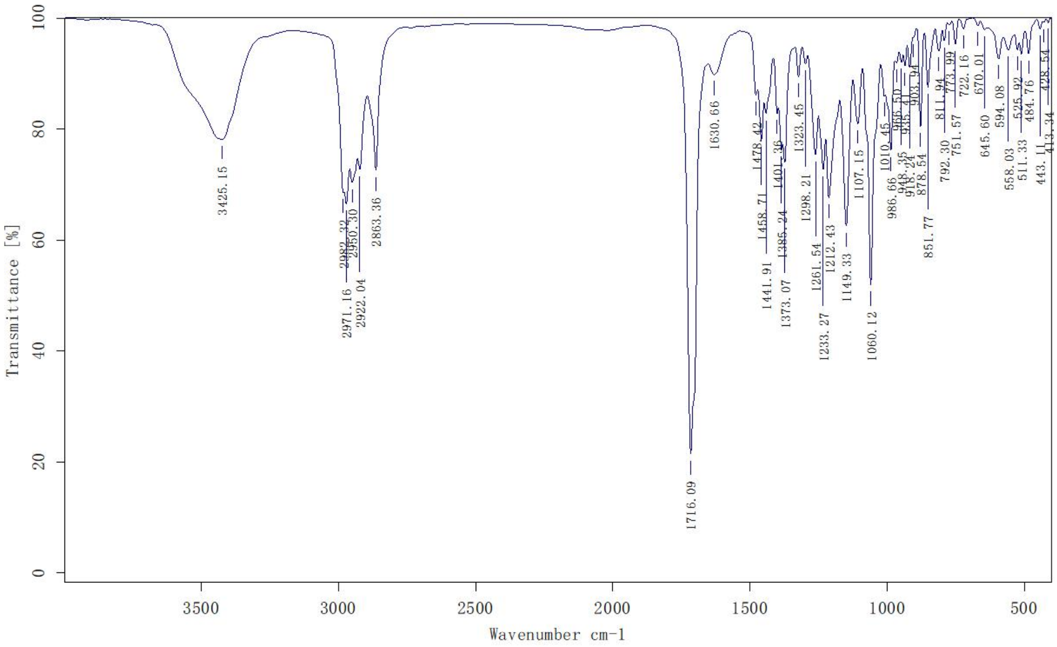

Figure SI41.[1] H NMR spectrum of compound **5** ($CDCl_3$)

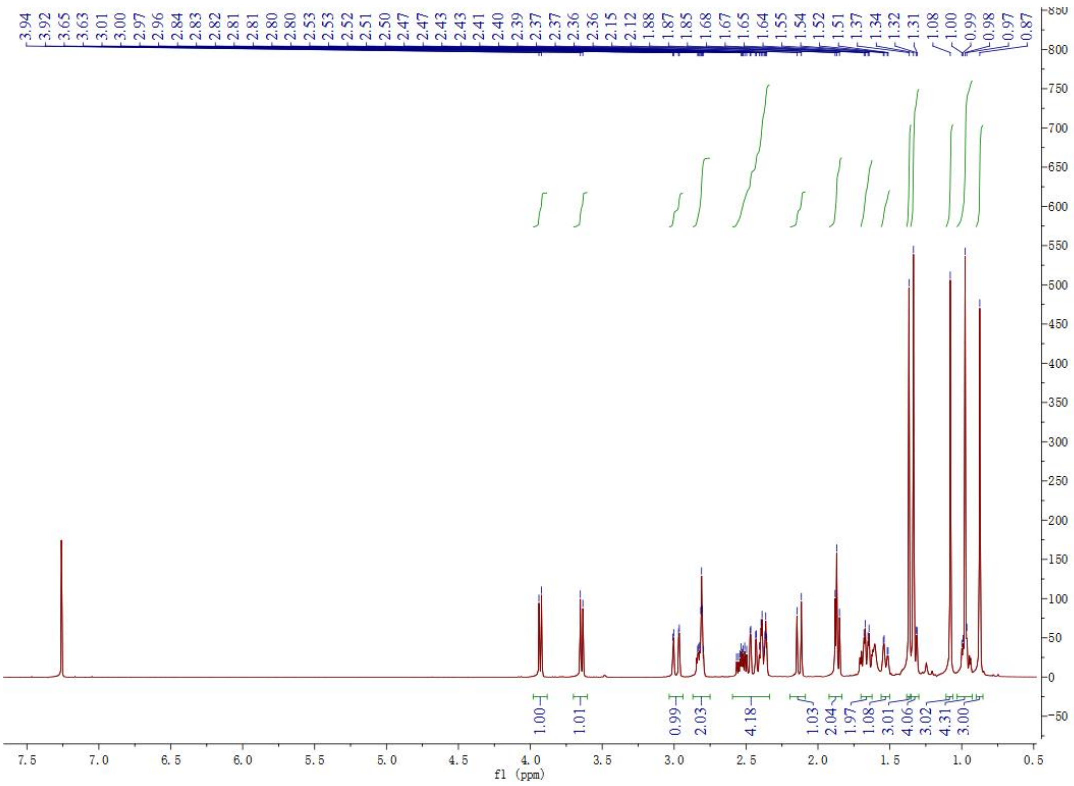

Figure SI42.^{13}C and DEPT spectrum of compound **5** ($CDCl_3$)

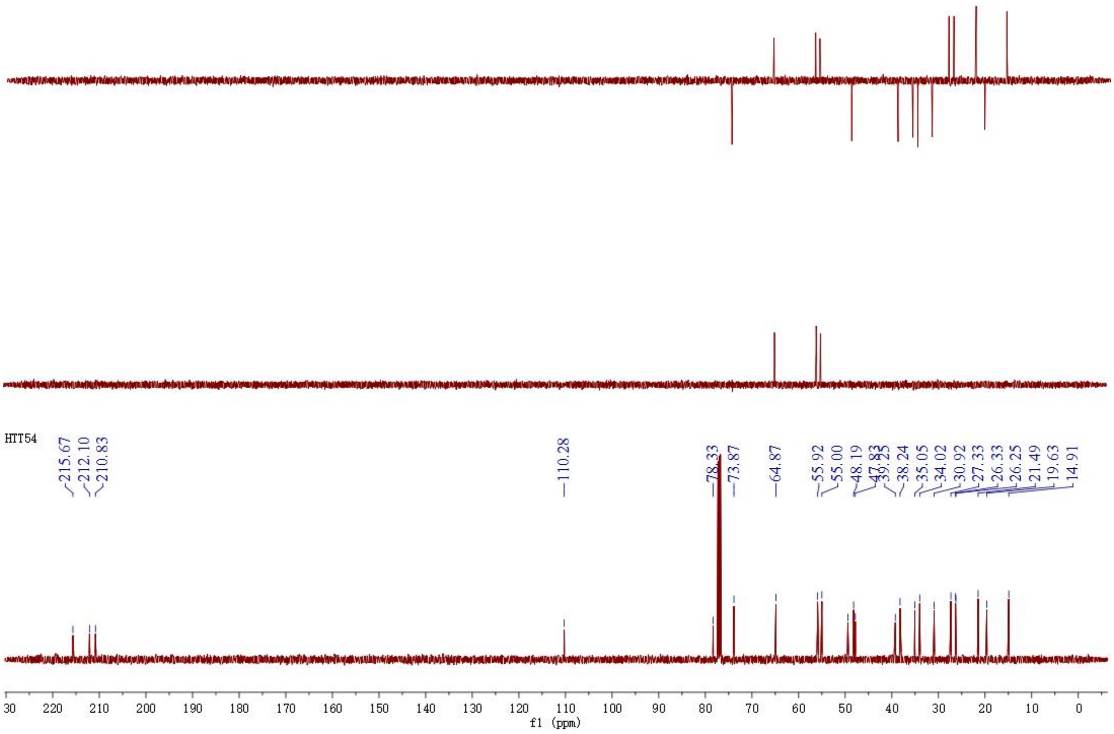

Figure SI43. HSQC spectrum of compound **5** (CDCl3)

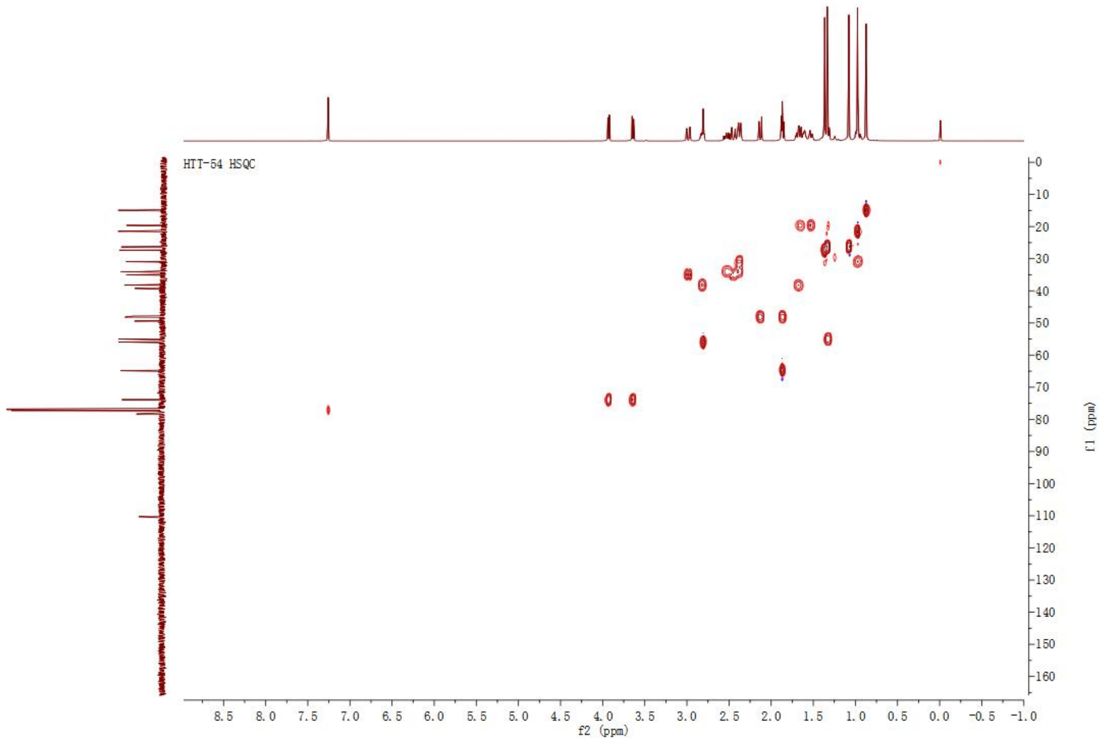

Figure SI44. HMBC spectrum of compound **5**(CDCl3)

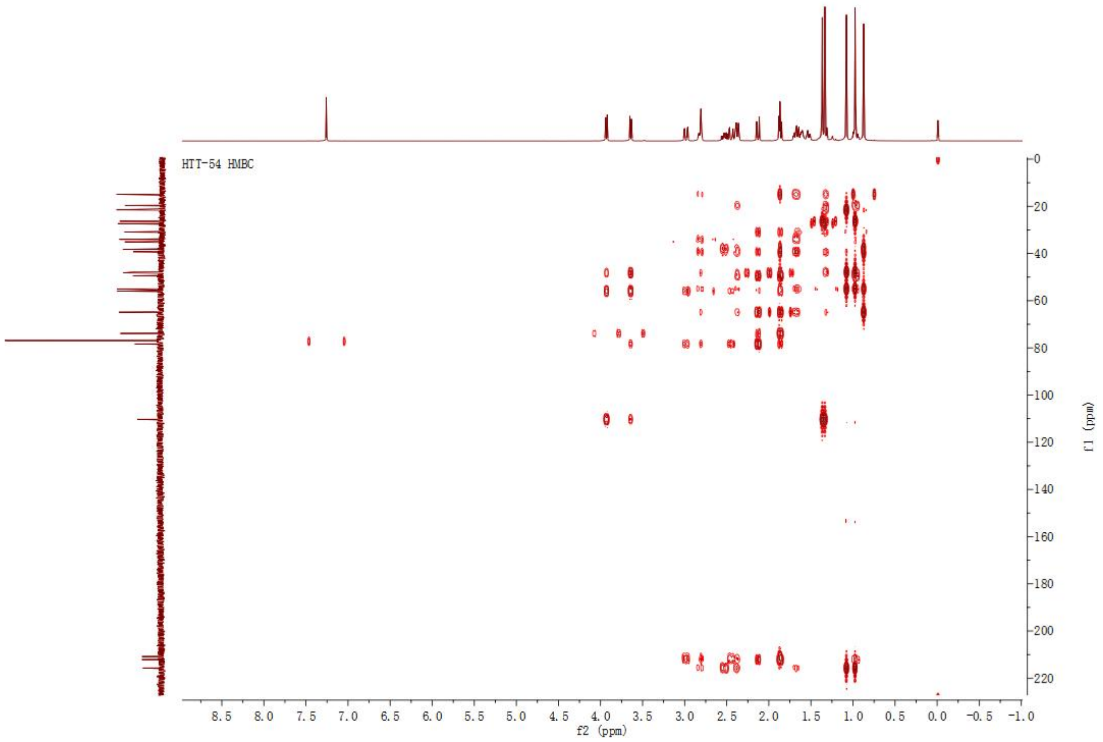

Figure SI45. ^1H-^1H COSY spectrum of compound **5** ($CDCl_3$)

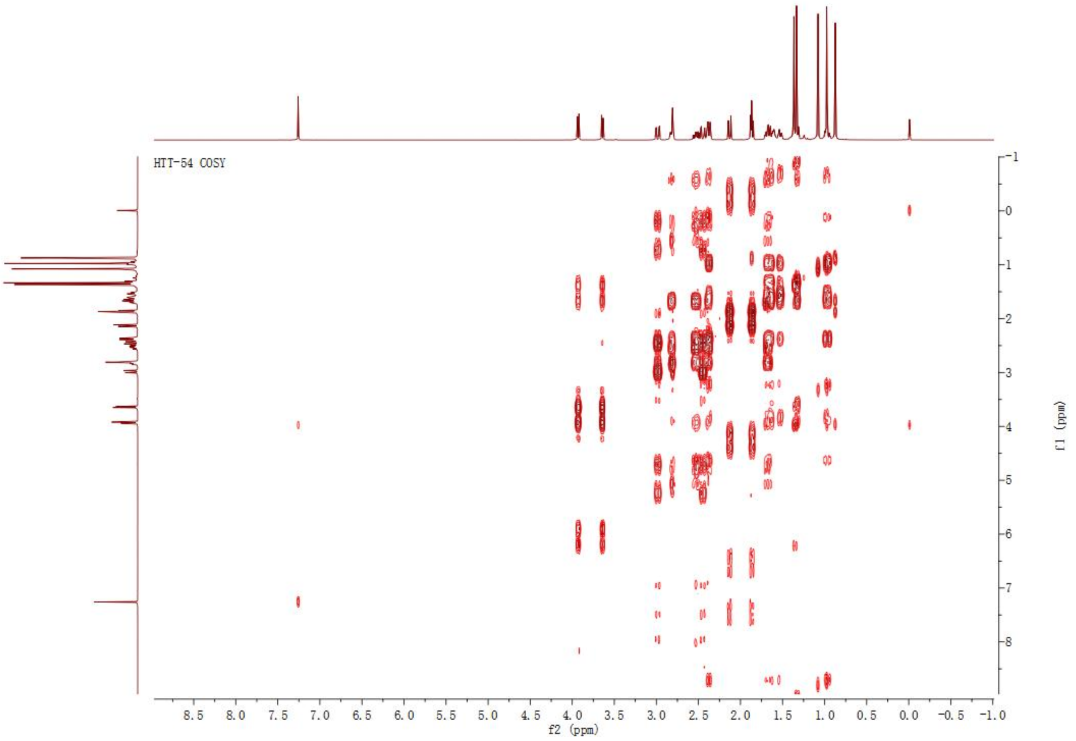

Figure SI46. ROESY spectrum of compound **5** ($CDCl_3$)

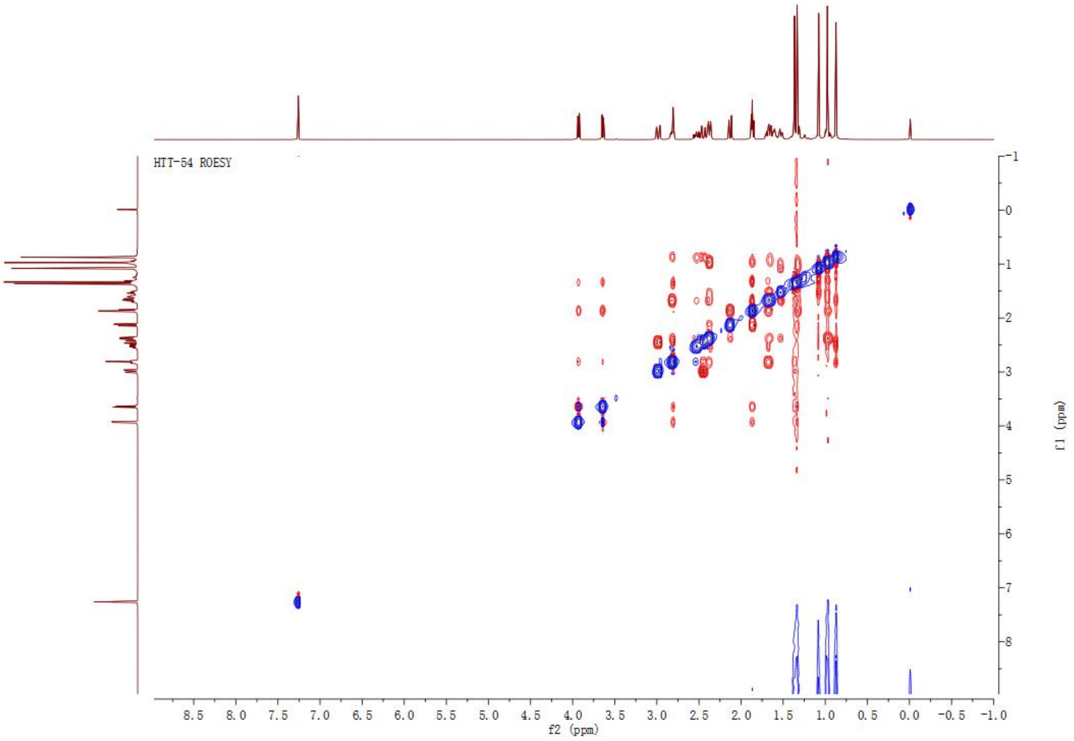

Figure SI47. HRESIMS spectrum of compound **6** (CD_3OD)

Qualitative Analysis Report

**Data Filename**	HTT-39a-.d	**Sample Name**	HTT-39a
**Sample Type**	Sample	**Position**	P1-A2
**Instrument Name**	Instrument 1	**User Name**	
**Acq Method**	s-.m	**Acquired Time**	5/8/2023 4:32:24 PM
**IRM Calibration Status**	Success	**DA Method**	PCDL.m
**Comment**			

**Sample Group**		**Info.**
**Acquisition SW Version**	6200 series TOF/6500 series Q-TOF B.05.01 (B5125.2)	

User Spectra

Fragmentor Voltage	Collision Energy	Ionization Mode
135	0	ESI

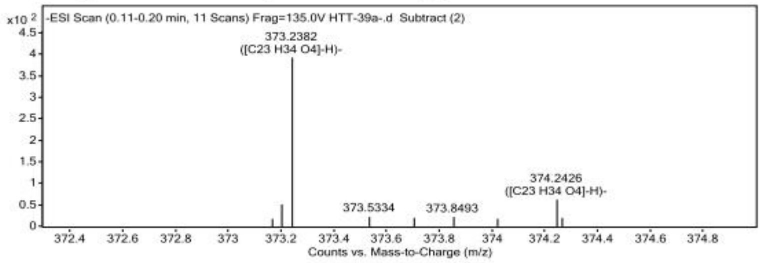

Peak List

m/z	z	Abund
255.2329	1	3411.23
301.0716	1	5976.93
313.108	1	6118.77
315.0879	1	1892.65
375.2753	1	5212.18
403.3066	1	8378.76
404.31	1	2074.51
419.2432	1	4753
459.2016	1	4193.27
1019.9548	1	2686.71

Formula Calculator Element Limits

Element	Min	Max
C	3	60
H	0	120
O	0	30
N	0	5

Formula Calculator Results

Formula	CalculatedMass	CalculatedMz	Mz	Diff. (mDa)	Diff. (ppm)	DBE
C23 H34 O4	374.2457	373.2384	373.2382	0.20	0.54	7.0000

--- End Of Report ---

Agilent Technologies Page 1 of 1 Printed at: 4:34 PM on: 5/8/2023

Figure SI48. UV spectrum of compound **6** (CD_3OD)

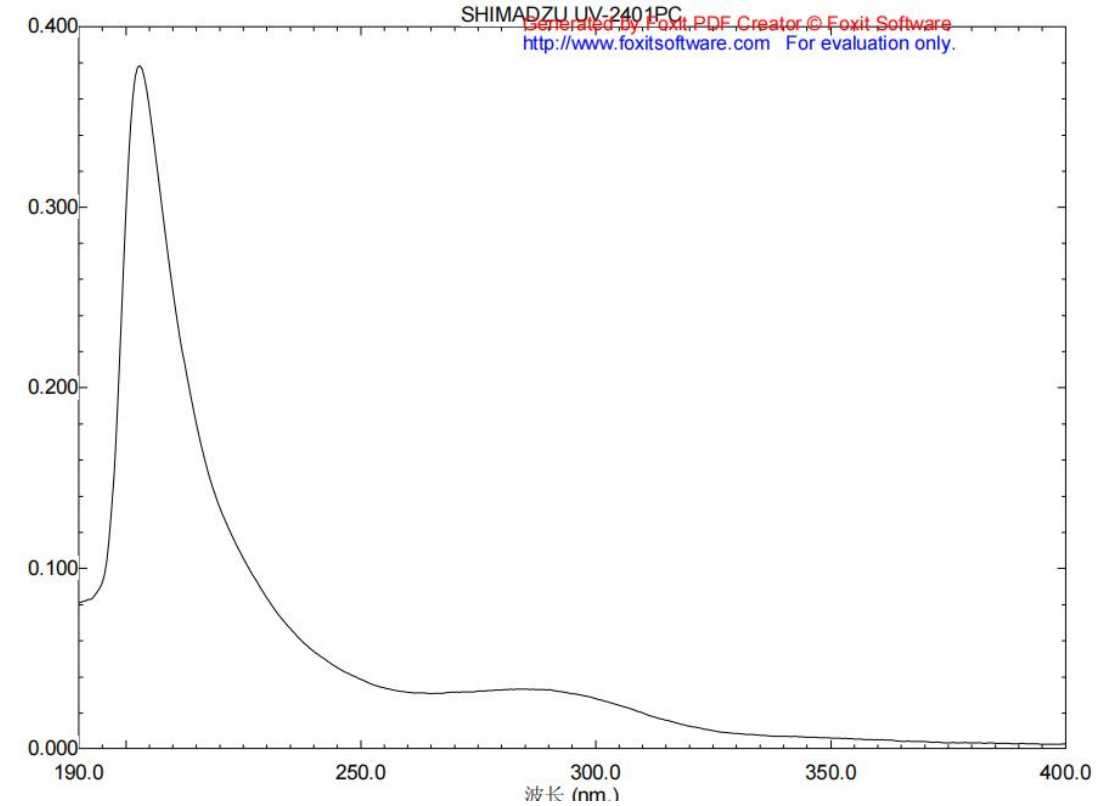

Generated by Foxit PDF Creator © Foxit Software
http://www.foxitsoftware.com For evaluation only.

Figure SI49. IR spectrum of compound **6** (CD_3OD)

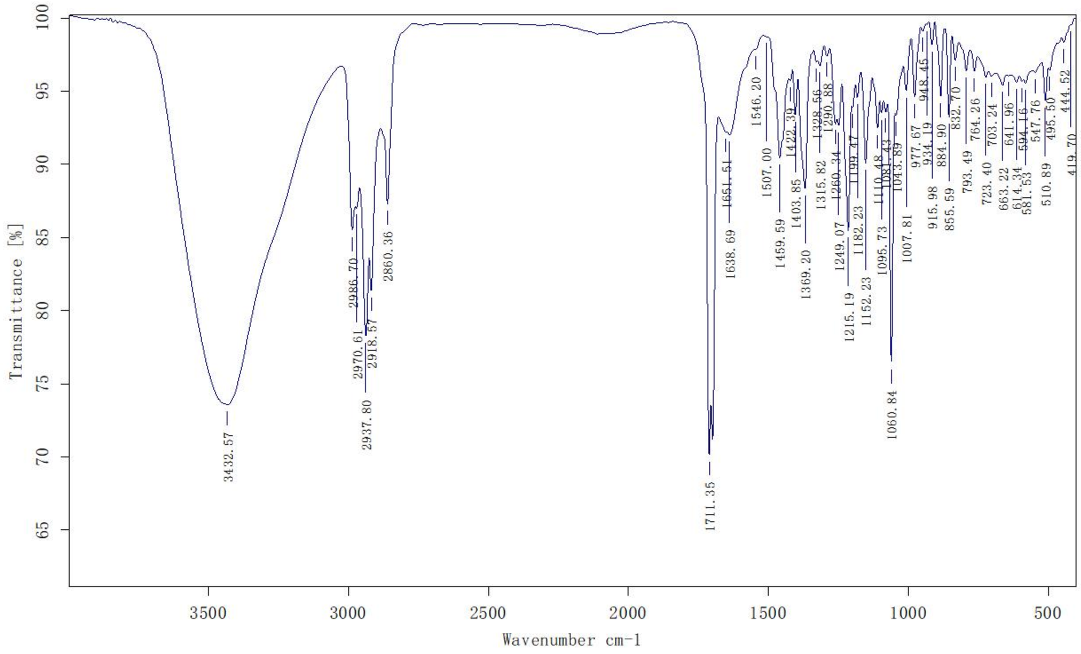

Figure SI50.[1] H NMR spectrum of compound **6** (CDCl3)

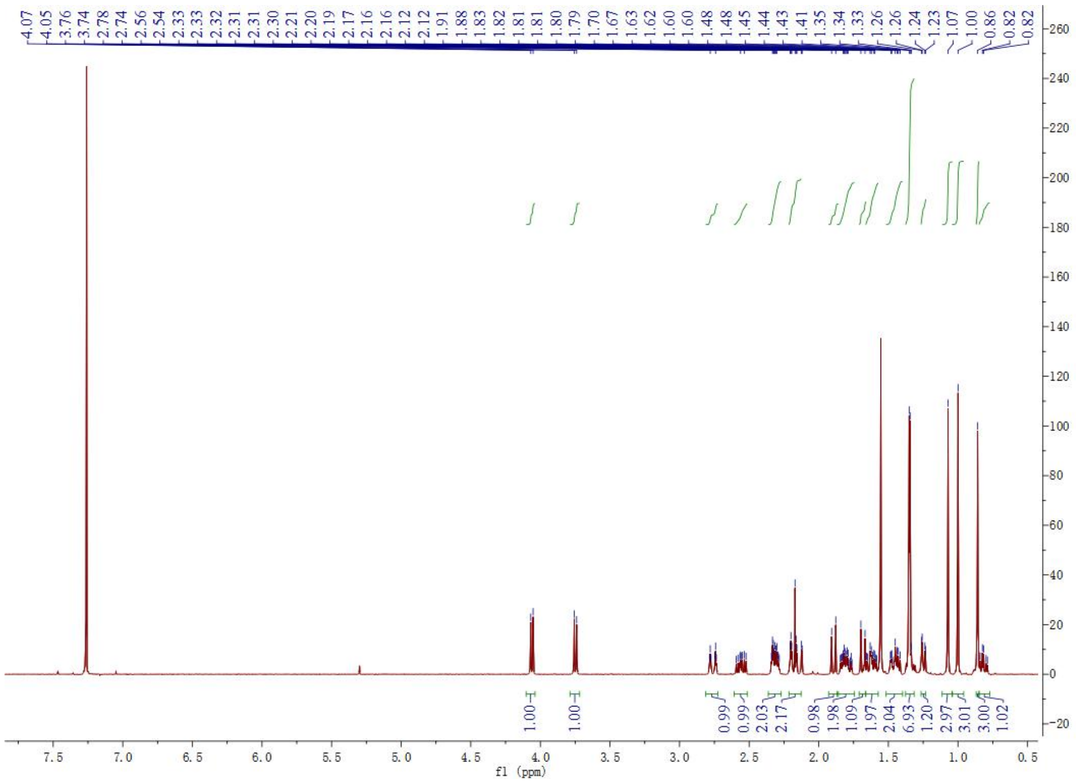

Figure SI51.^{13}C and DEPT spectrum of compound **6** (CDCl3)

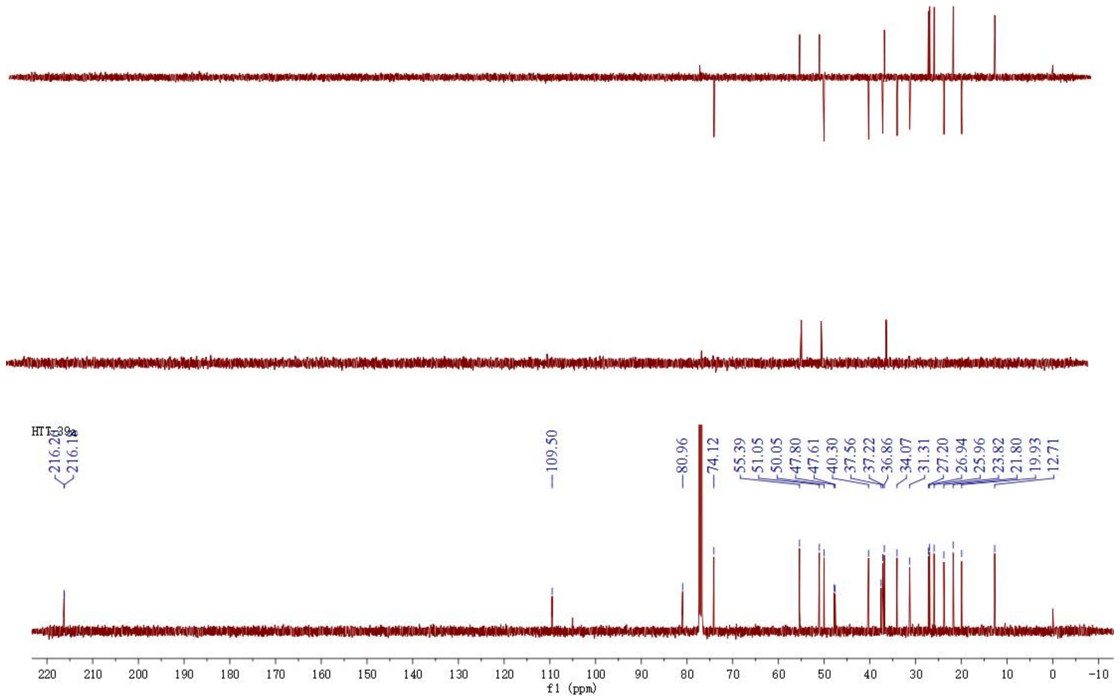

Figure SI52. HSQC spectrum of compound **6** (CDCl3)

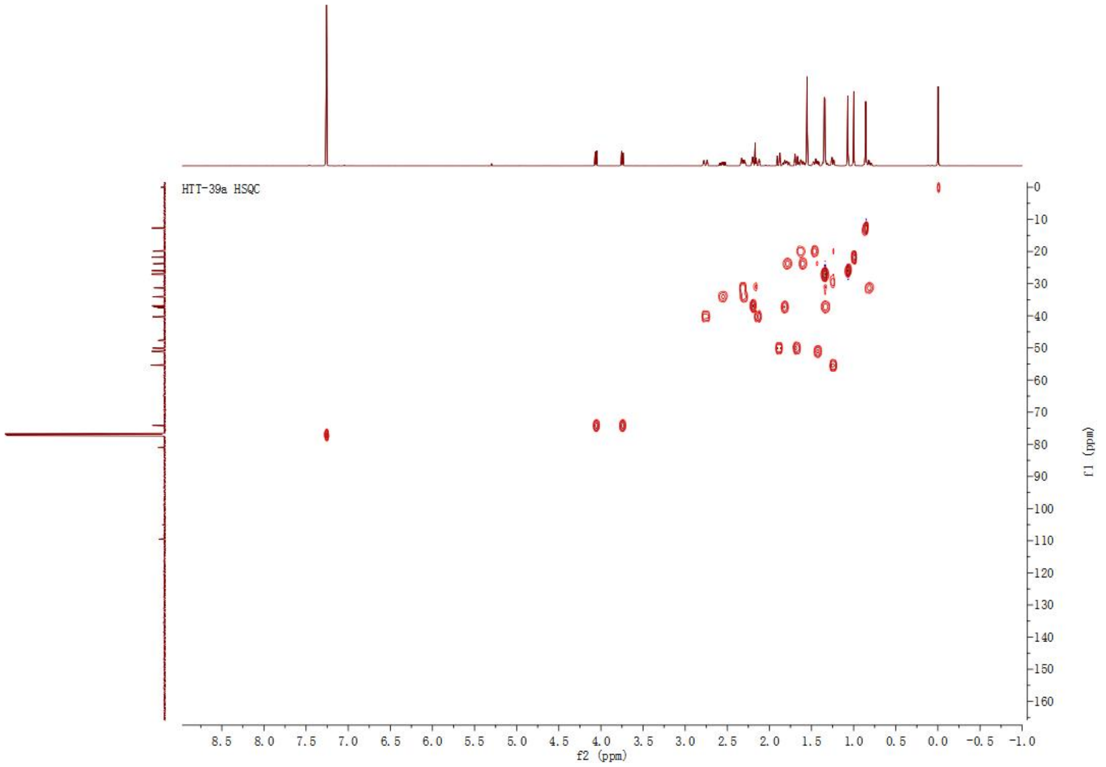

Figure SI53. HMBC spectrum of compound **6** (CDCl3)

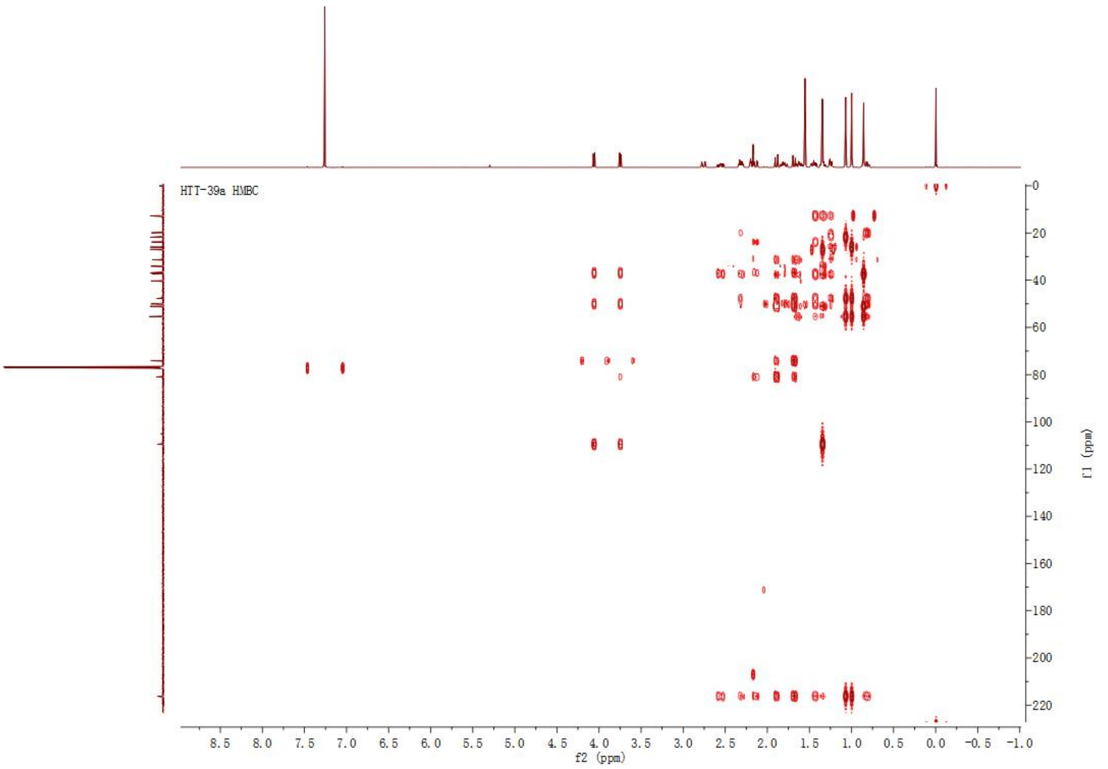

Figure SI54. ^{1}H-^{1}H COSY spectrum of compound **6** (CDCl3)

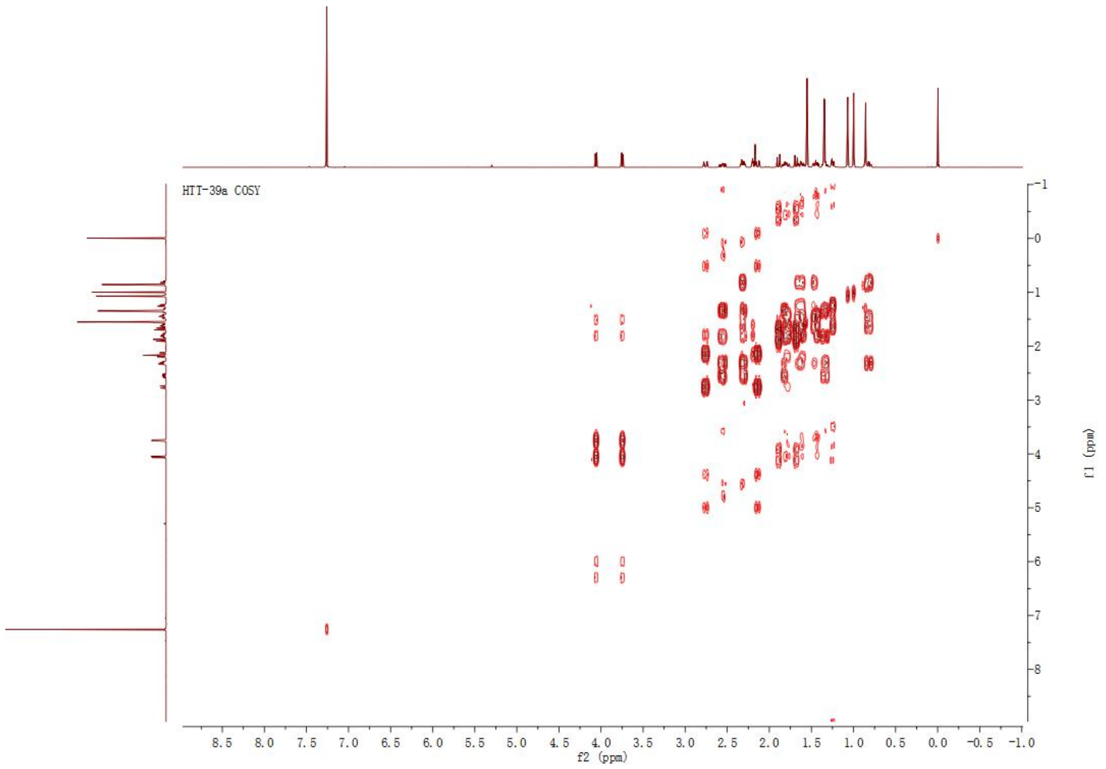

Figure SI55. ROESY spectrum of compound **6** (CDCl3)

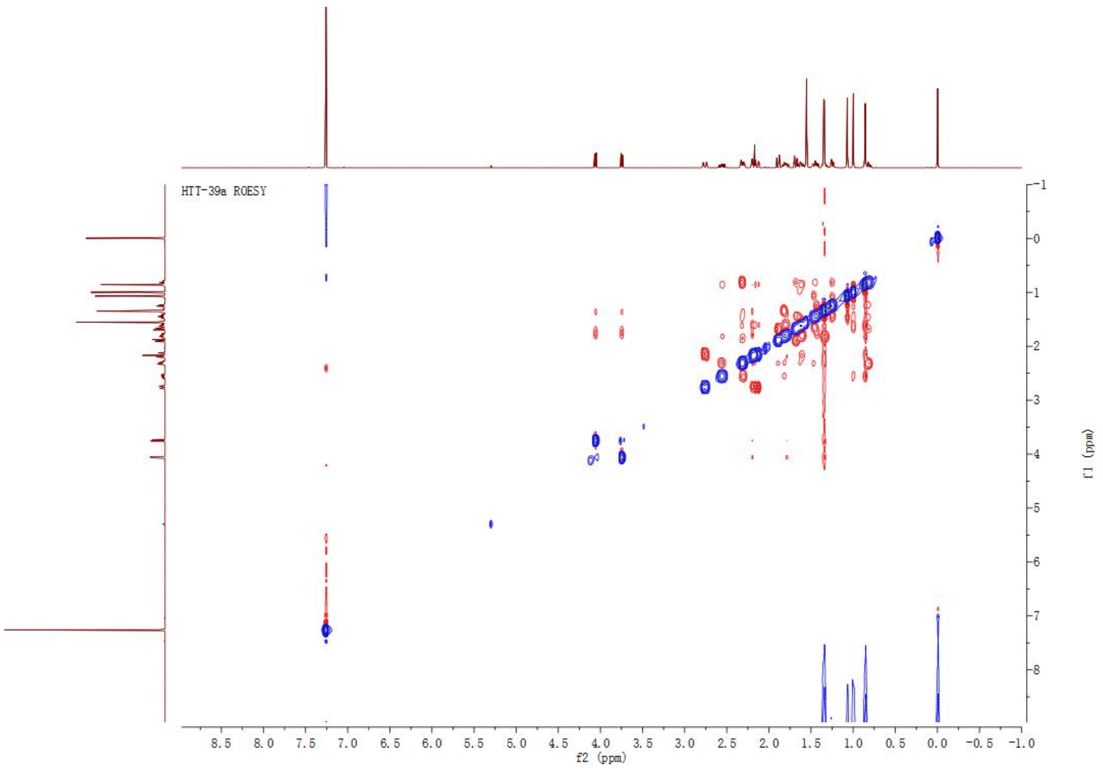

Figure SI56. HRESIMS spectrum of compound **7** (CD_3OD)

Qualitative Analysis Report

**Data Filename**	HTT-55-.d	**Sample Name**	HTT-55
**Sample Type**	Sample	**Position**	P1-B3
**Instrument Name**	Instrument 1	**User Name**	
**Acq Method**	s-.m	**Acquired Time**	7/7/2023 11:50:43 AM
**IRM Calibration Status**	Success	**DA Method**	PCDL.m
**Comment**			

**Sample Group**		**Info.**
**Acquisition SW**	6200 series TOF/6500 series	
**Version**	Q-TOF B.05.01 (B5125.2)	

User Spectra

Fragmentor Voltage	Collision Energy	Ionization Mode
135	0	ESI

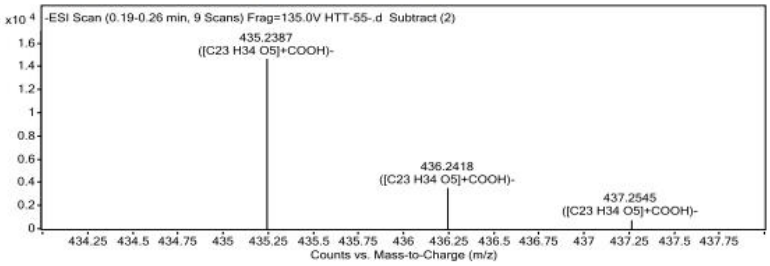

Peak List

m/z	z	Abund	Formula	Ion
44.9983	1	1757.84		
89.0242	1	785.1		
375.2755	1	889.54		
403.3062	1	1240.85		
423.2402	1	2533.81		
435.2387	1	14702.27	C23 H34 O5	(M+COOH)-
436.2418	1	3585.81	C23 H34 O5	(M+COOH)-
437.2545	1	881.39	C23 H34 O5	(M+COOH)-
1019.9503	1	1009.62		
1051.9459	1	1126.08		

Formula Calculator Element Limits

Element	Min	Max
C	3	60
H	0	200
O	0	20

Formula Calculator Results

Formula	CalculatedMass	CalculatedMz	Mz	Diff. (mDa)	Diff. (ppm)	DBE
C23 H34 O5	390.2406	435.2388	435.2387	0.10	0.23	7.0000

--- End Of Report ---

Figure SI57. UV spectrum of compound **7** (CD_3OD)

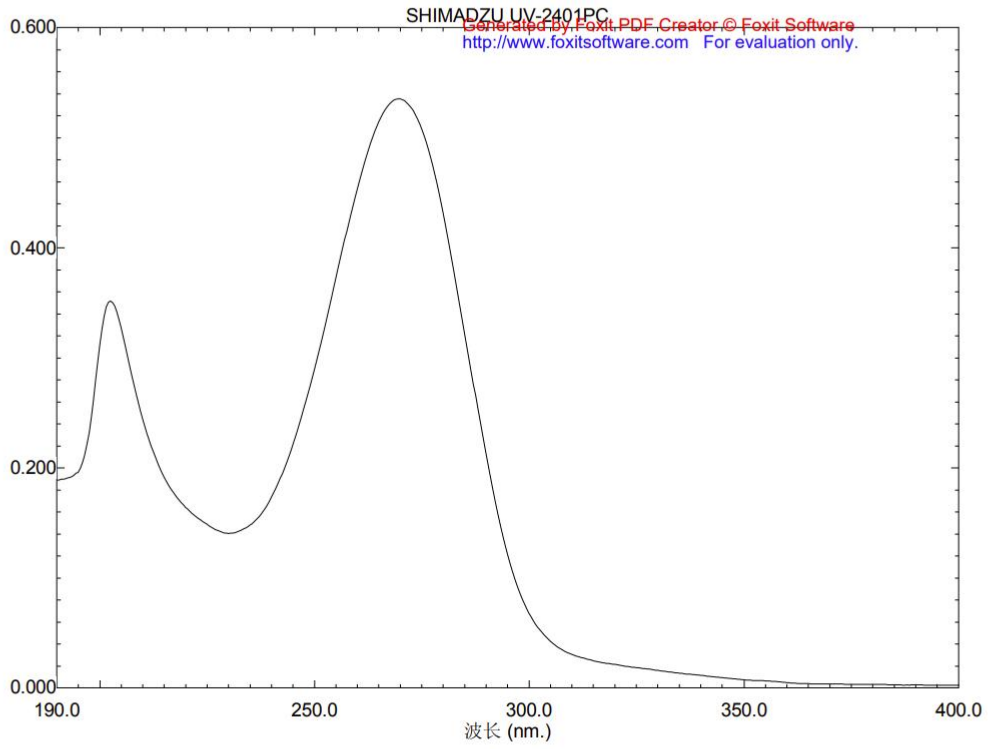

Figure SI58. IR spectrum of compound **7** (CD_3OD)

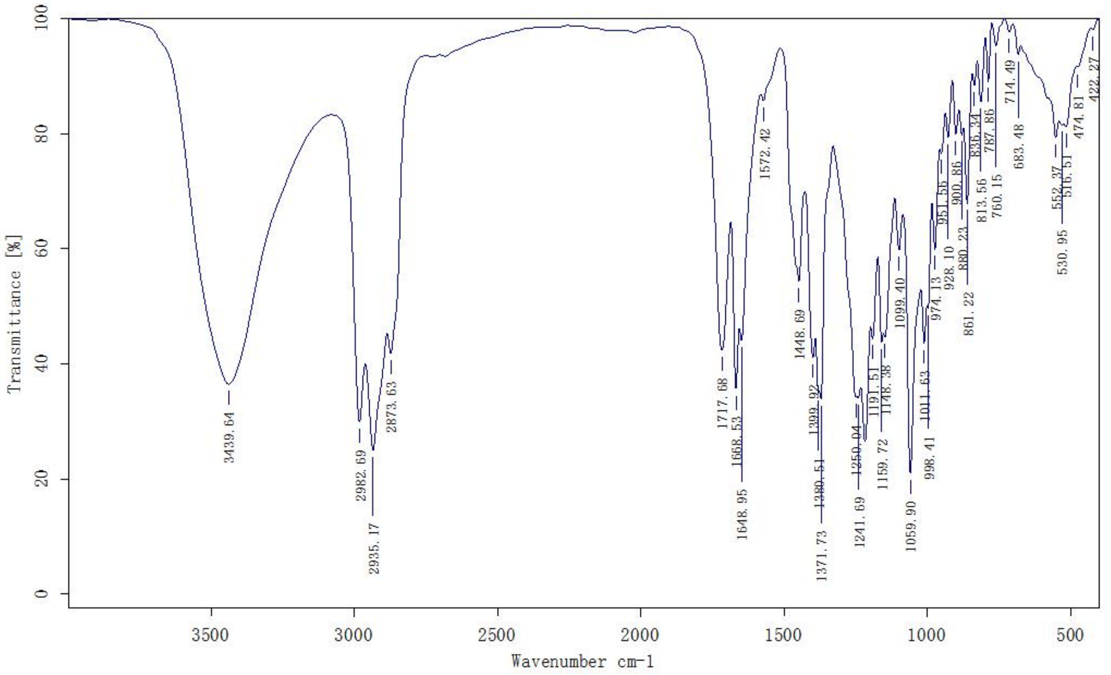

Figure SI59.[1] H NMR spectrum of compound **7** ($CDCl_3$)

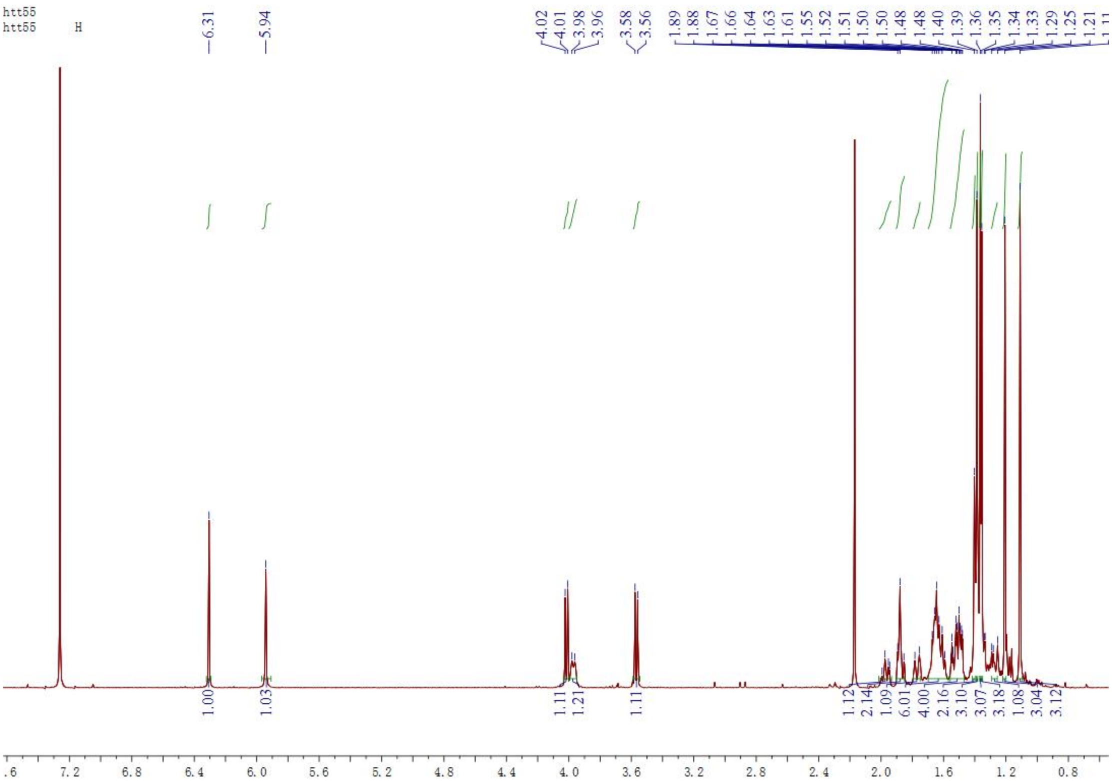

Figure SI60.^{13}C and DEPT spectrum of compound **7** ($CDCl_3$)

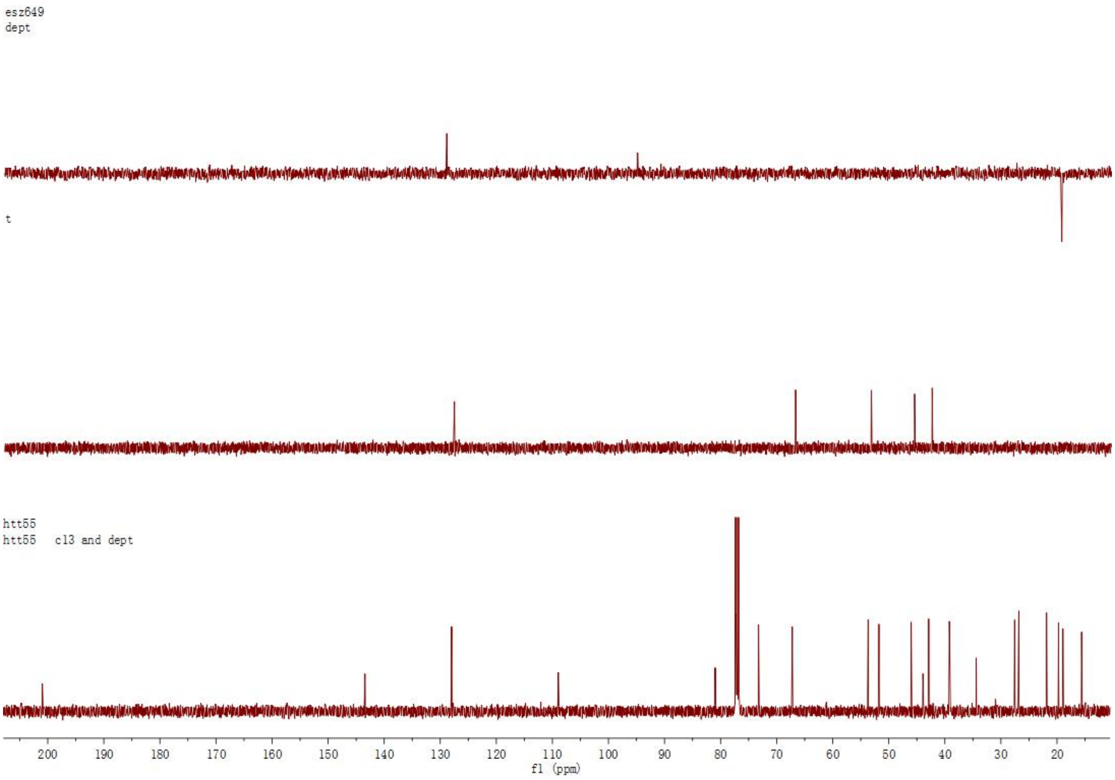

Figure SI61. HSQC spectrum of compound **7** ($CDCl_3$)

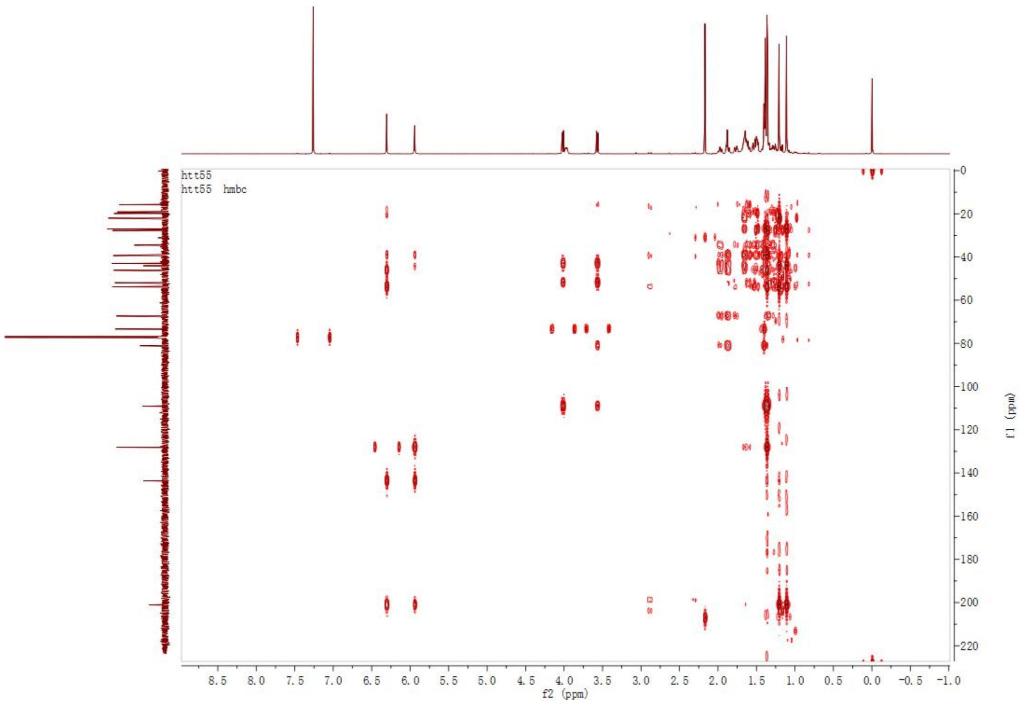

Figure SI62. HMBC spectrum of compound **7** ($CDCl_3$)

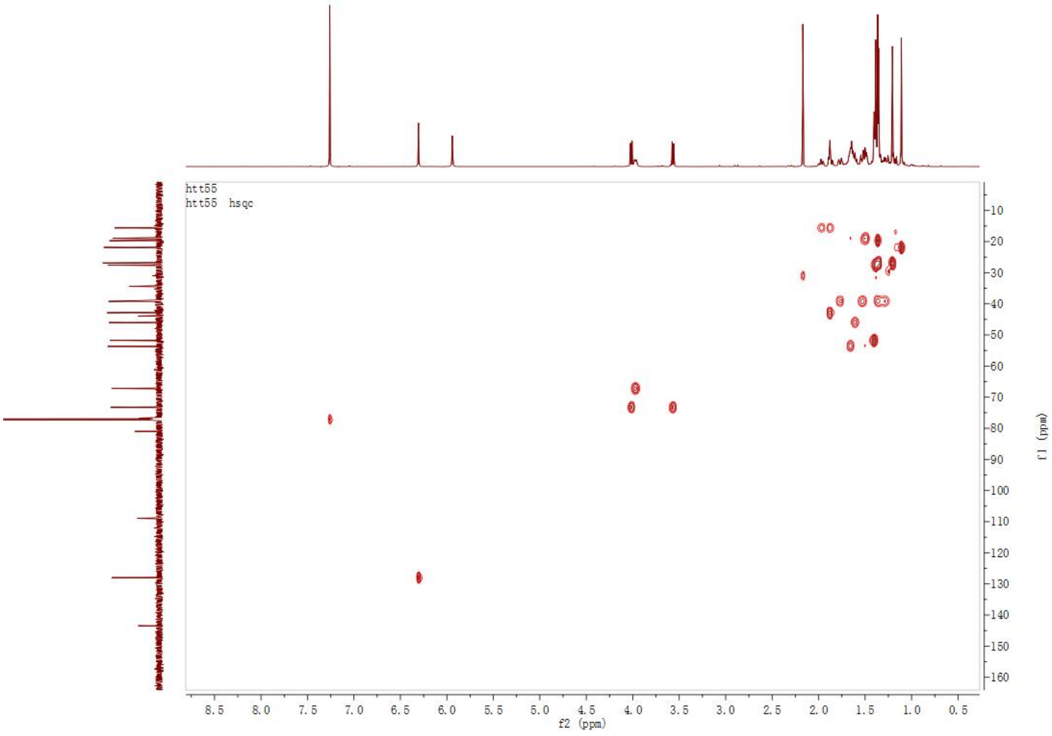

Figure SI63. ^{1}H-^{1}H COSY spectrum of compound **7** ($CDCl_3$)

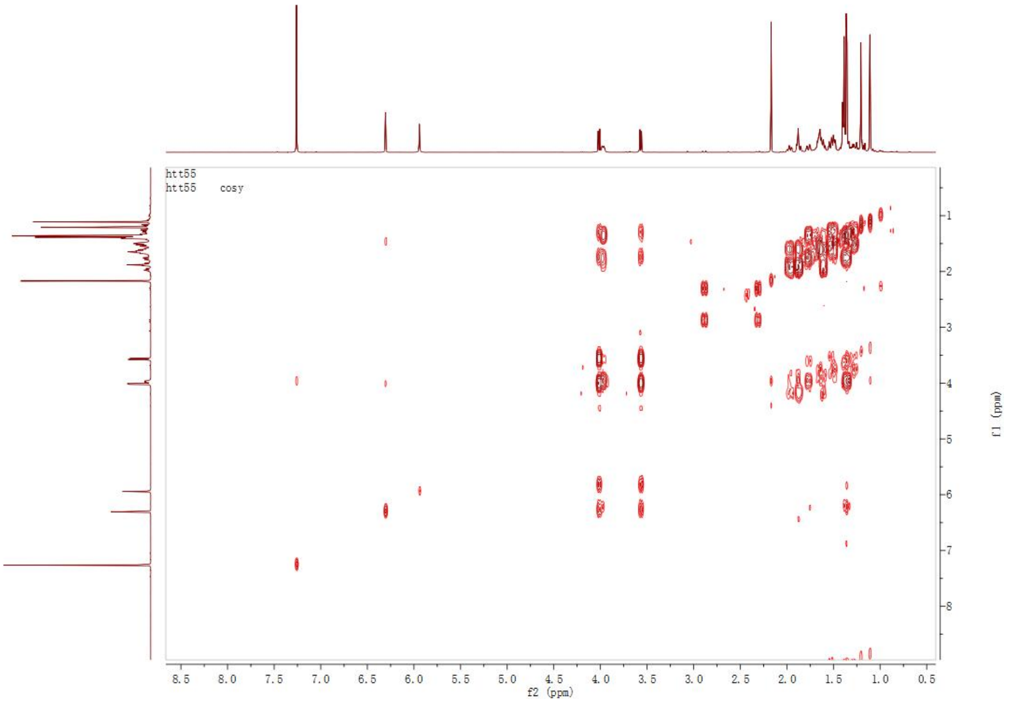

Figure SI64. ROESY spectrum of compound **7** ($CDCl_3$)

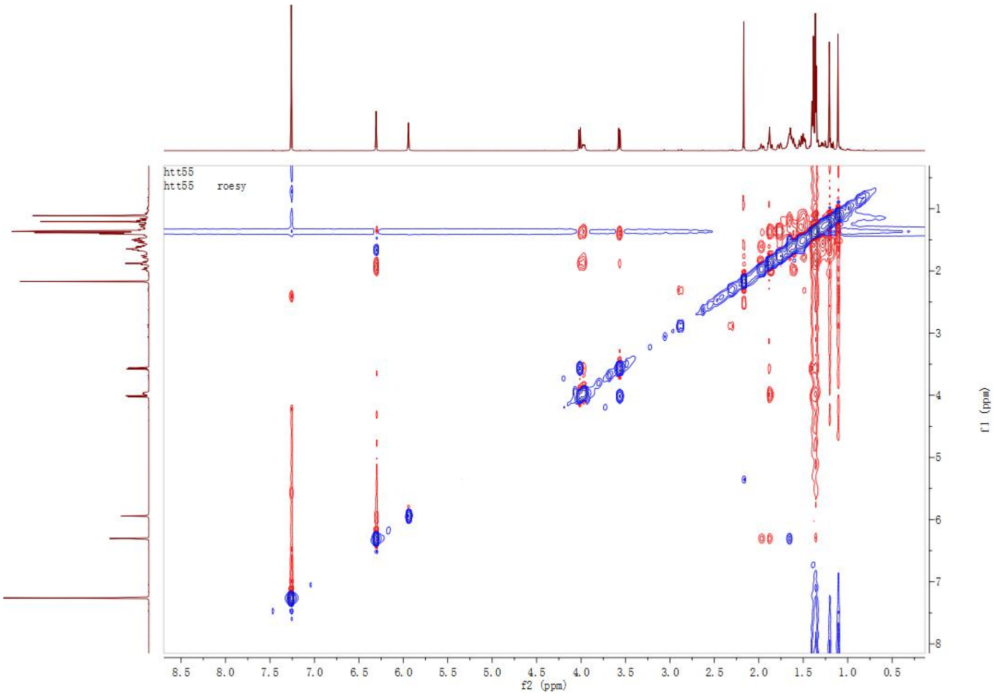

Figure SI56. HRESIMS spectrum of compound **8** (CD_3OD)

Qualitative Analysis Report

**Data Filename**	HTT-30.d	**Sample Name**	HTT-30
**Sample Type**	Sample	**Position**	P1-F1
**Instrument Name**	Instrument 1	**User Name**	
**Acq Method**	s.m	**Acquired Time**	4/14/2023 11:11:45 AM
**IRM Calibration Status**	Success	**DA Method**	PCDL.m
**Comment**			

Sample Group **Info.**
Acquisition SW 6200 series TOF/6500 series
Version Q-TOF B.05.01 (B5125.2)

User Spectra

Fragmentor Voltage	Collision Energy	Ionization Mode
135	0	ESI

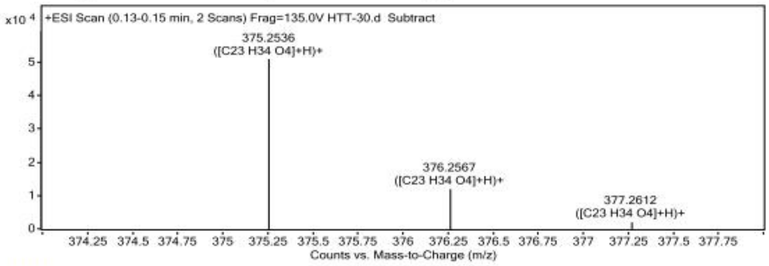

Peak List

m/z	z	Abund	Formula	Ion
317.2121	1	1447.47		
375.2536	1	51181.12	C23 H34 O4	(M+H)+
376.2567	1	12269.56	C23 H34 O4	(M+H)+
377.2612	1	2418.57	C23 H34 O4	(M+H)+
397.2353	1	14148.22		
398.2381	1	3531.67		
413.209	1	1373.52		
771.4802	1	11994.29		
772.4846	1	5698.16		
773.4836	1	1639.3		

Formula Calculator Element Limits

Element	Min	Max
C	3	60
H	0	120
O	0	30
N	0	5

Formula Calculator Results

Formula	CalculatedMass	CalculatedMz	Mz	Diff. (mDa)	Diff. (ppm)	DBE
C23 H34 O4	374.2457	375.2530	375.2536	-0.60	-1.60	7.0000

--- End Of Report ---

Agilent Technologies Page 1 of 1 Printed at: 11:22 AM on: 4/14/2023

Figure SI57. UV spectrum of compound **8** (CD_3OD)

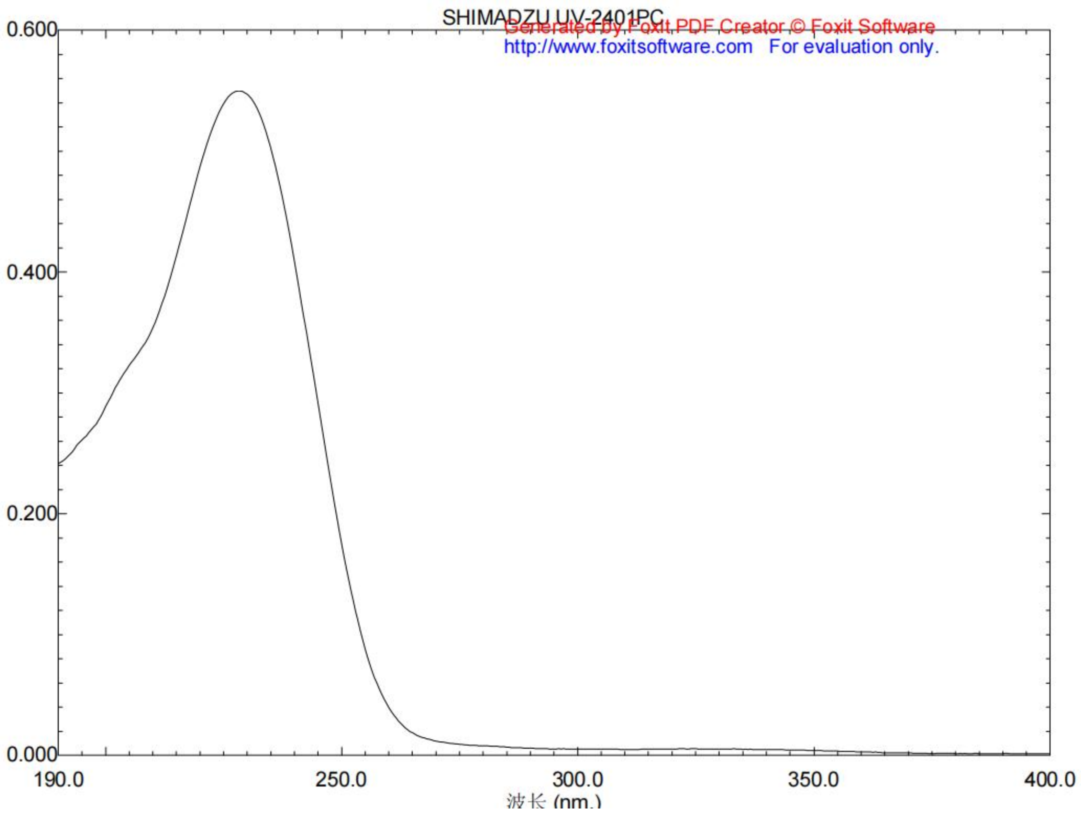

Figure SI58. IR spectrum of compound **8** (CD_3OD)

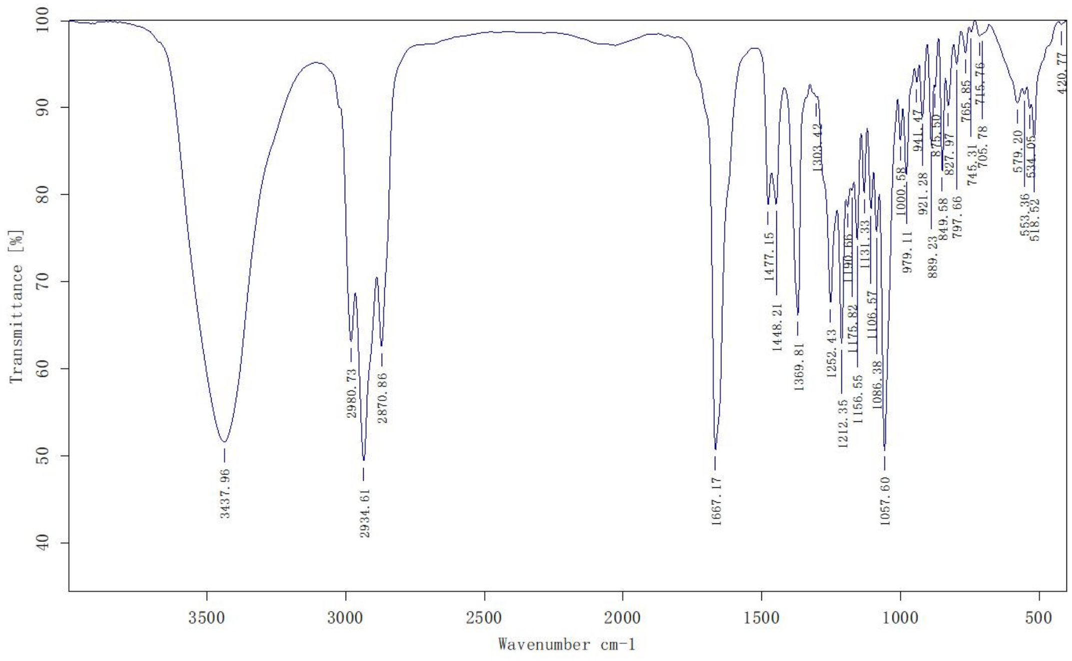

Figure SI59.[1] H NMR spectrum of compound **8** ($CDCl_3$)

Figure SI59.[1] H NMR spectrum of compound **8** (CDCl3)

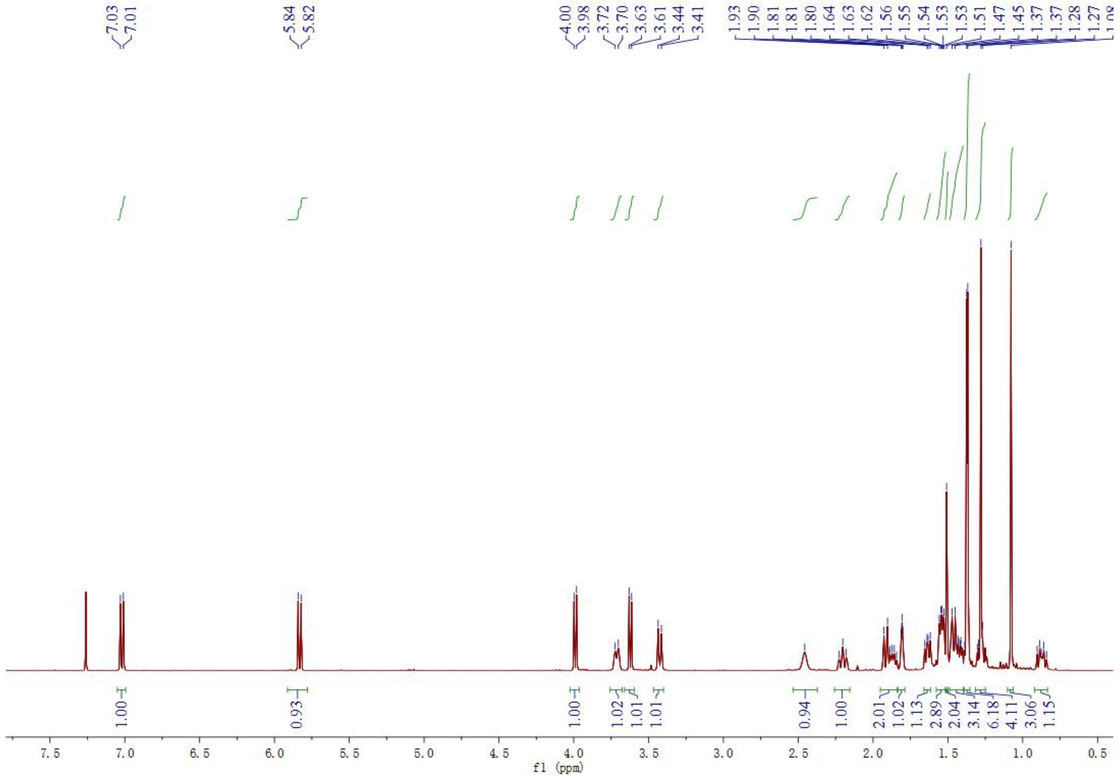

Figure SI60.^{13}C and DEPT spectrum of compound **8** (CDCl3)

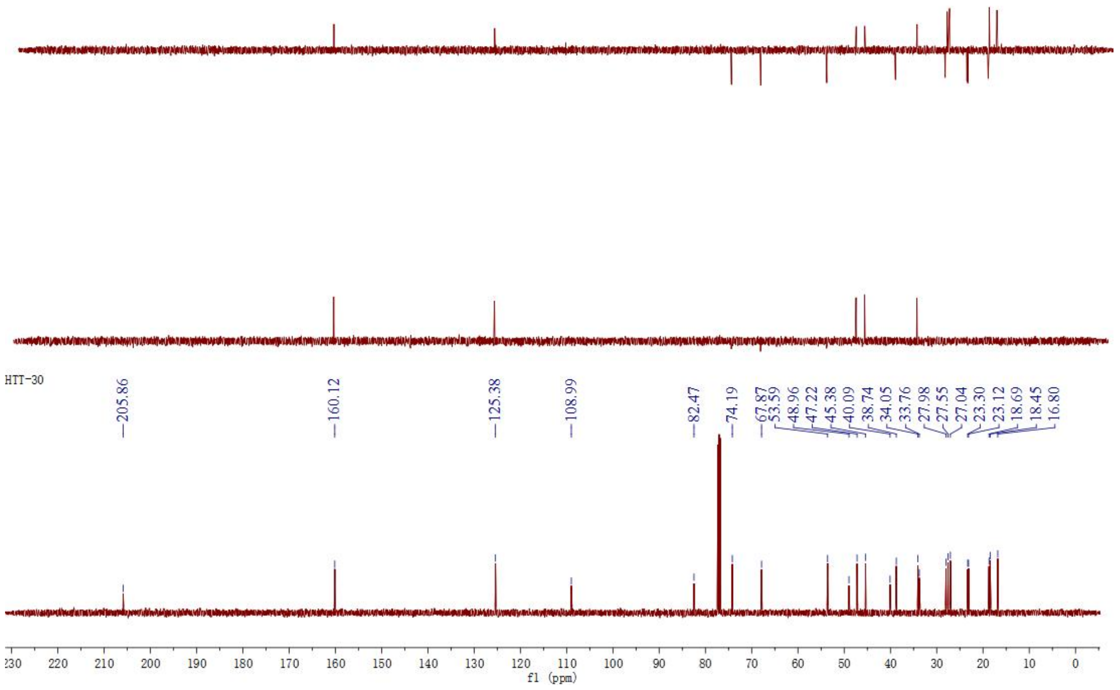

Figure SI61. HSQC spectrum of compound **8** ($CDCl_3$)

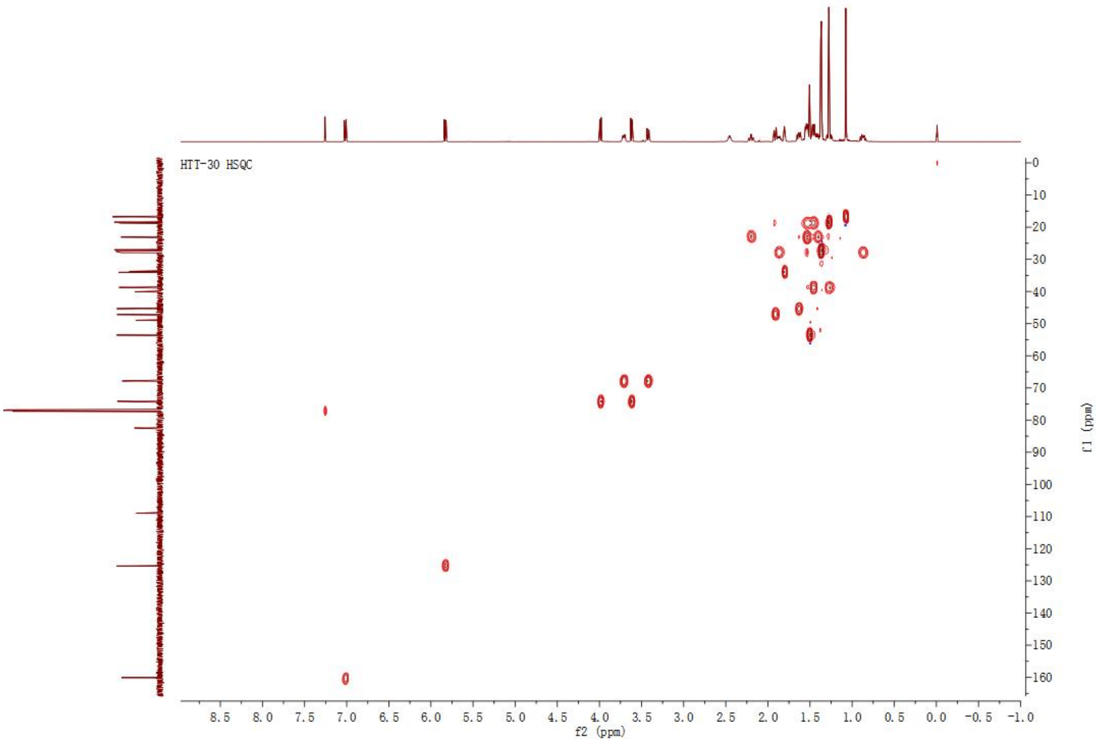

Figure SI62. HMBC spectrum of compound **8** ($CDCl_3$)

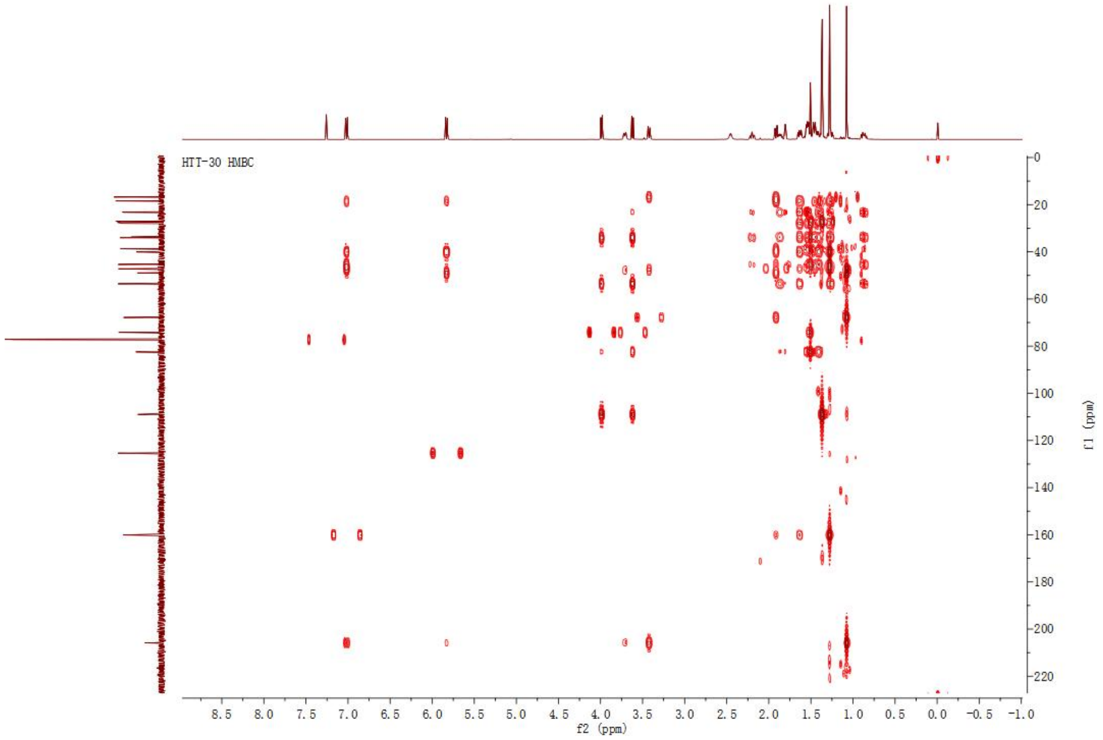

Figure SI63. ^1H-^1H COSY spectrum of compound **8** ($CDCl_3$)

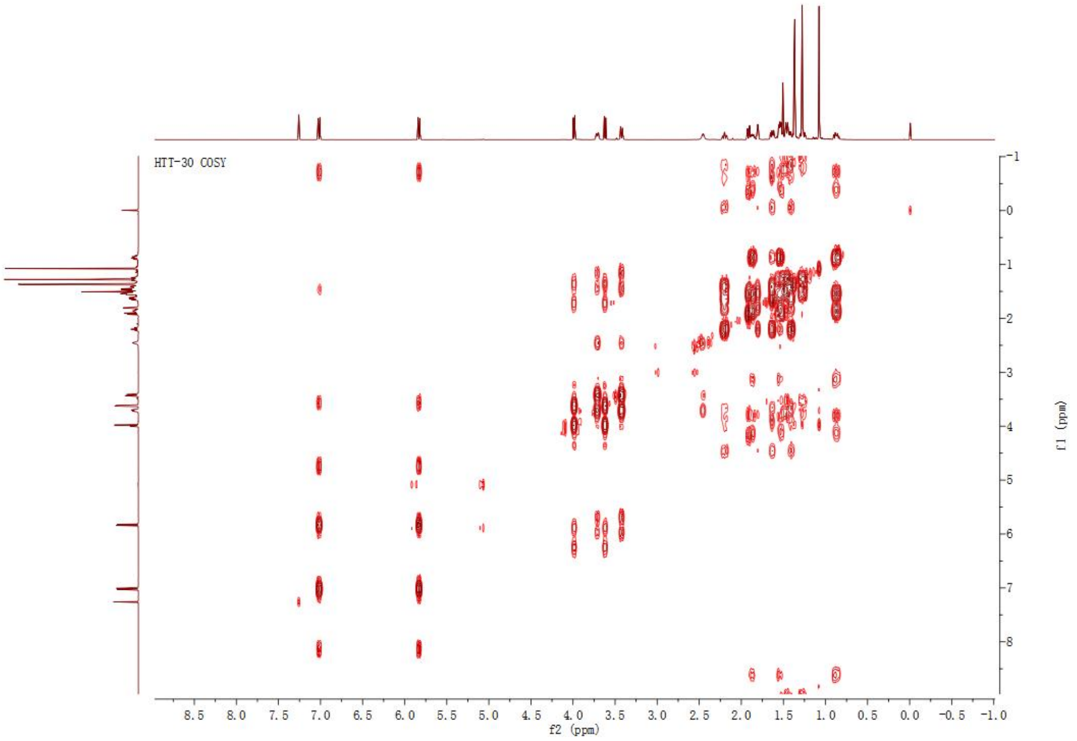

Figure SI64. ROESY spectrum of compound **8** ($CDCl_3$)

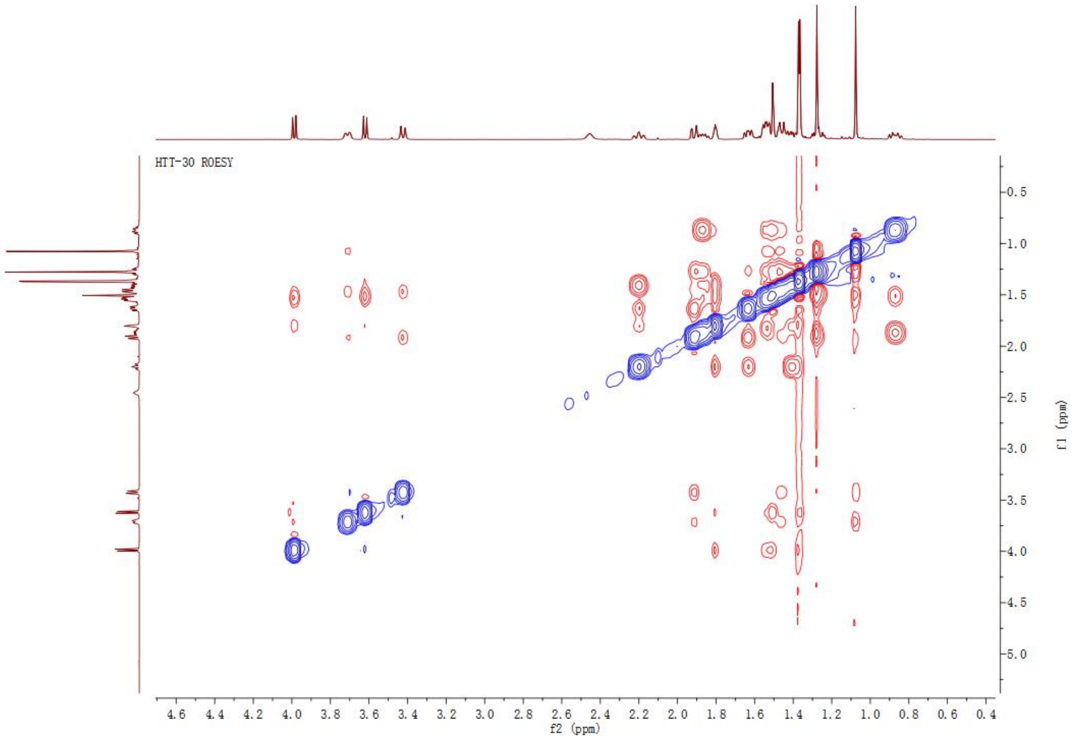

Figure SI74. HRESIMS spectrum of compound **9** (CD_3OD)

Qualitative Analysis Report

**Data Filename**	HTT-36.d	**Sample Name**	HTT-36
**Sample Type**	Sample	**Position**	P1-F1
**Instrument Name**	Instrument 1	**User Name**	
**Acq Method**	s.m	**Acquired Time**	1/30/2024 4:20:43 PM
**IRM Calibration Status**	Success	**DA Method**	PCDL.m
**Comment**			

Sample Group **Info.**
Acquisition SW Version 6200 series TOF/6500 series Q-TOF B.05.01 (B5125.2)

User Spectra

Fragmentor Voltage	Collision Energy	Ionization Mode
165	30	ESI

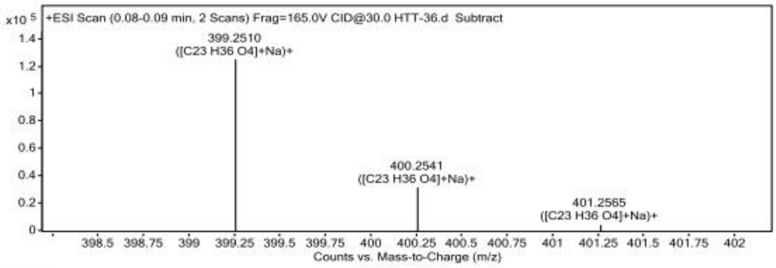

Peak List

m/z	z	Abund	Formula	Ion
399.251	1	125367.2	C23 H36 O4	(M+Na)+
400.2541	1	32058.54	C23 H36 O4	(M+Na)+
401.2565	1	5140.8	C23 H36 O4	(M+Na)+
429.2249	1	10517.25		
430.228	1	2713.86		
443.2406	1	54859.44		
444.2433	1	15461.61		
459.2717	1	4575.79		
467.238	1	3486.28		
484.2275	1	5166.33		

Formula Calculator Element Limits

Element	Min	Max
C	3	100
H	0	200
O	0	30

Formula Calculator Results

Formula	CalculatedMass	CalculatedMz	Mz	Diff. (mDa)	Diff. (ppm)	DBE
C23 H36 O4	376.2614	399.2506	399.2510	-0.40	-1.00	6.0000

--- End Of Report ---

Agilent Technologies Page 1 of 1 Printed at: 4:46 PM on: 1/30/2024

Figure SI75. UV spectrum of compound **9** (CD_3OD)

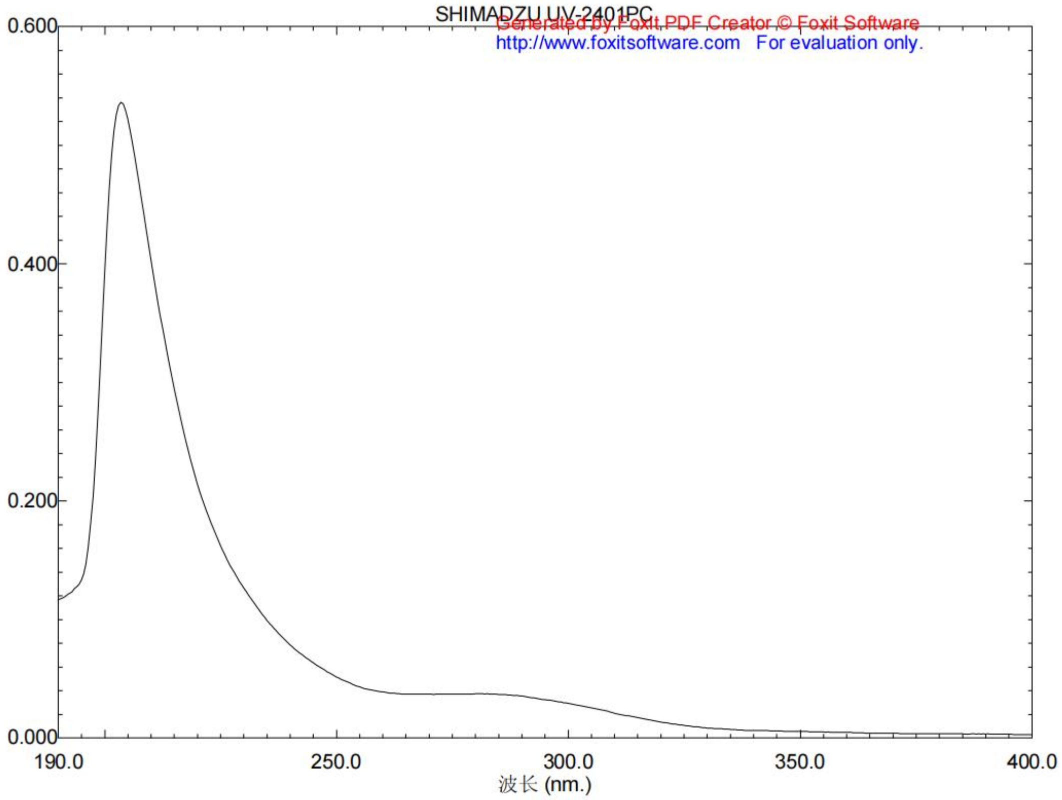

Figure SI76. IR spectrum of compound **9** (CD_3OD)

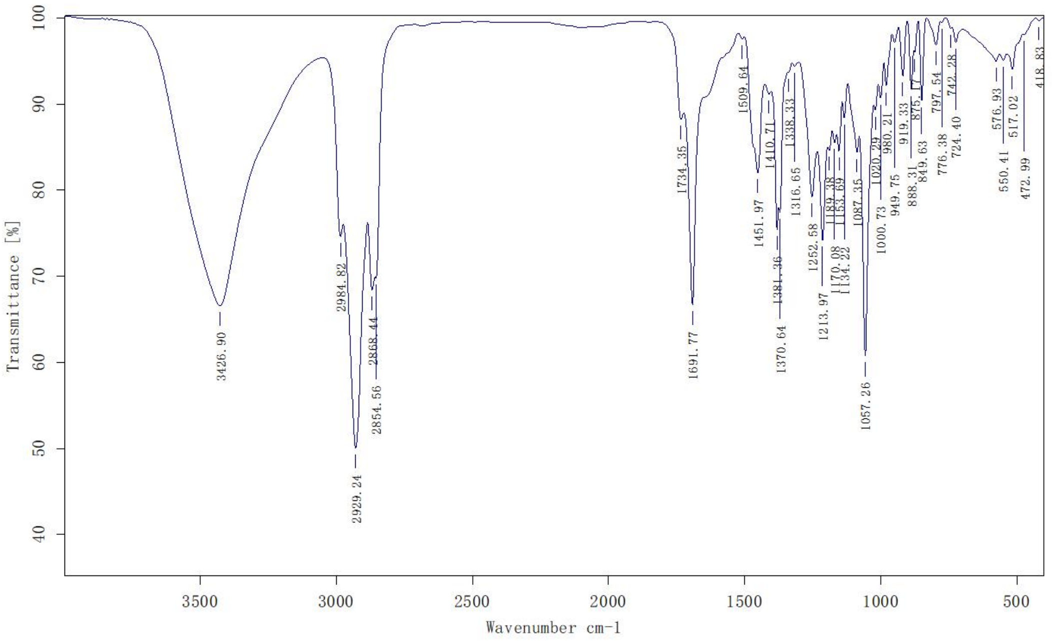

Figure SI77.[1] H NMR spectrum of compound **9** ($CDCl_3$)

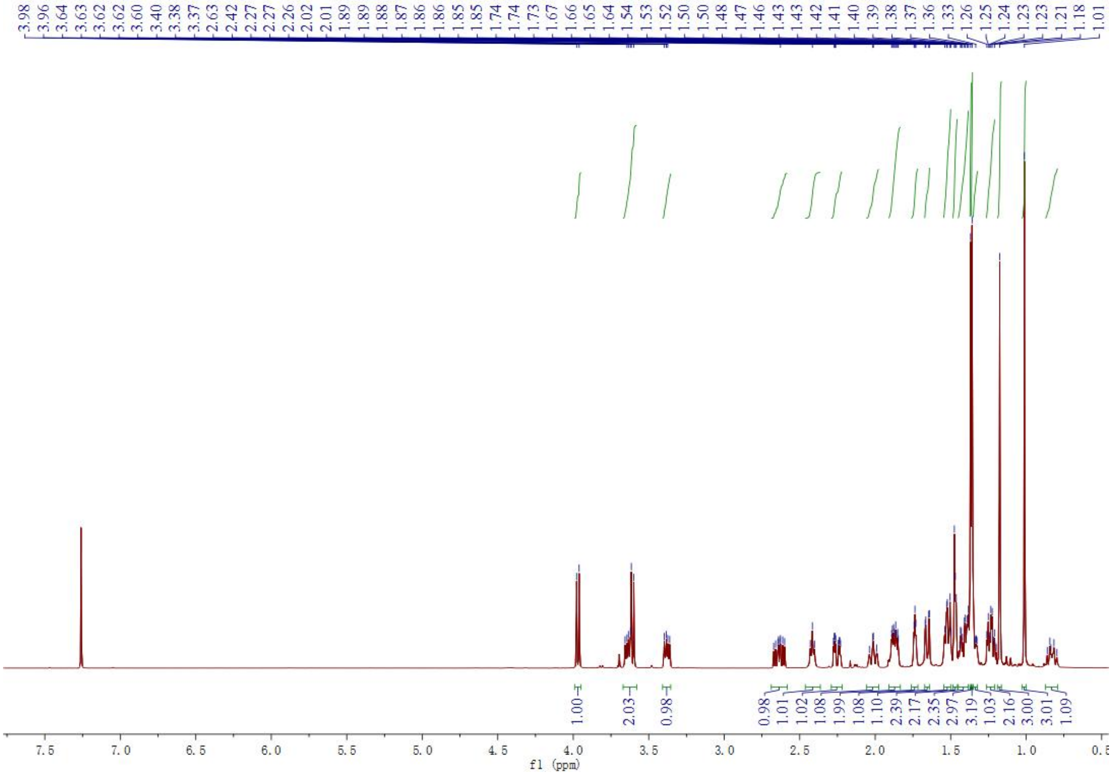

Figure SI78.^{13}C and DEPT spectrum of compound **9** ($CDCl_3$)

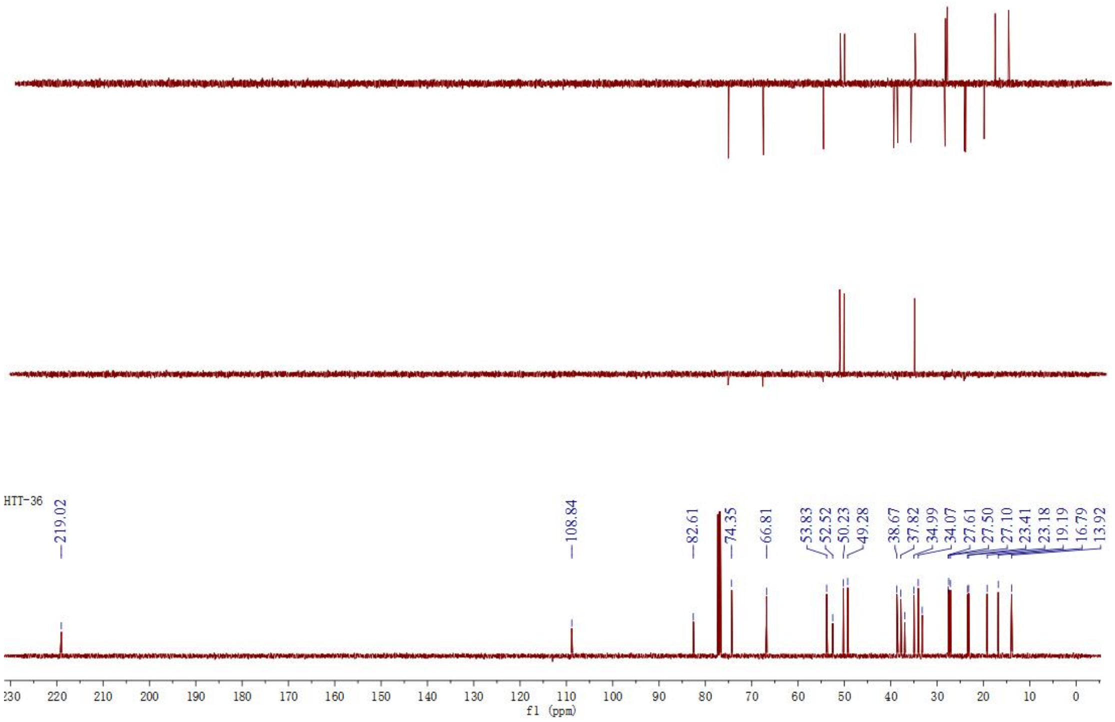

Figure SI79. HSQC spectrum of compound **9** ($CDCl_3$)

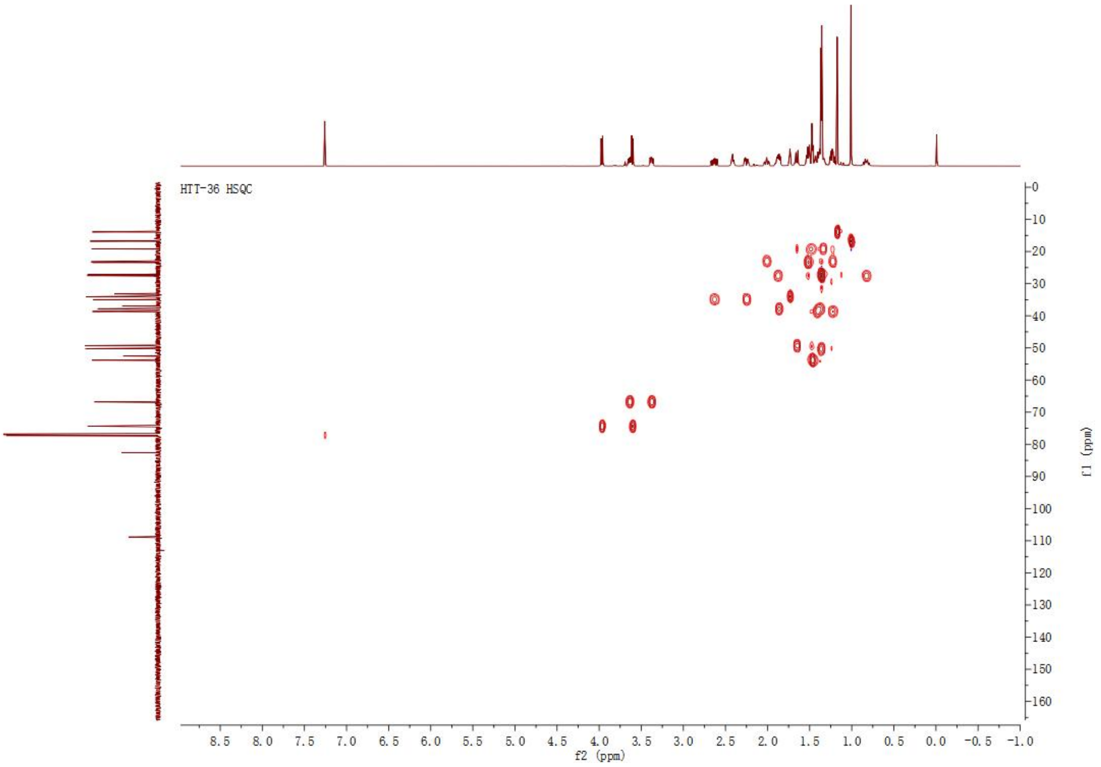

Figure SI80. HMBC spectrum of compound **9** ($CDCl_3$)

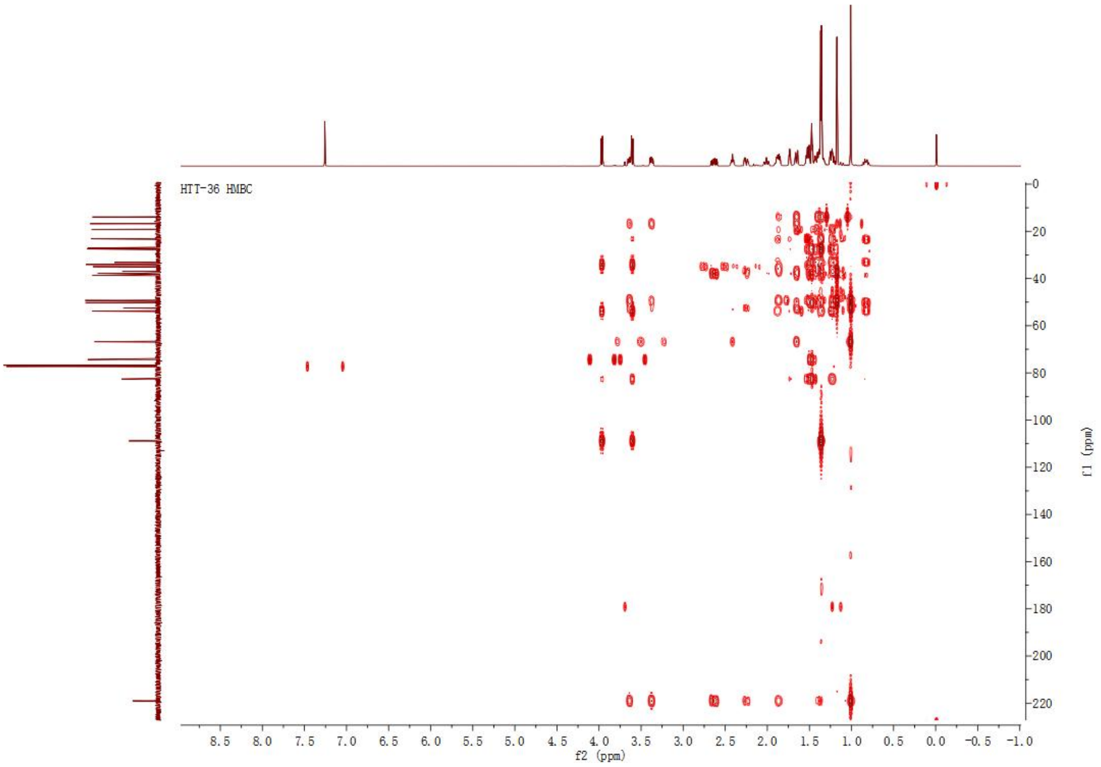

Figure SI81. ^{1}H-^{1}H COSY spectrum of compound **9** ($CDCl_3$)

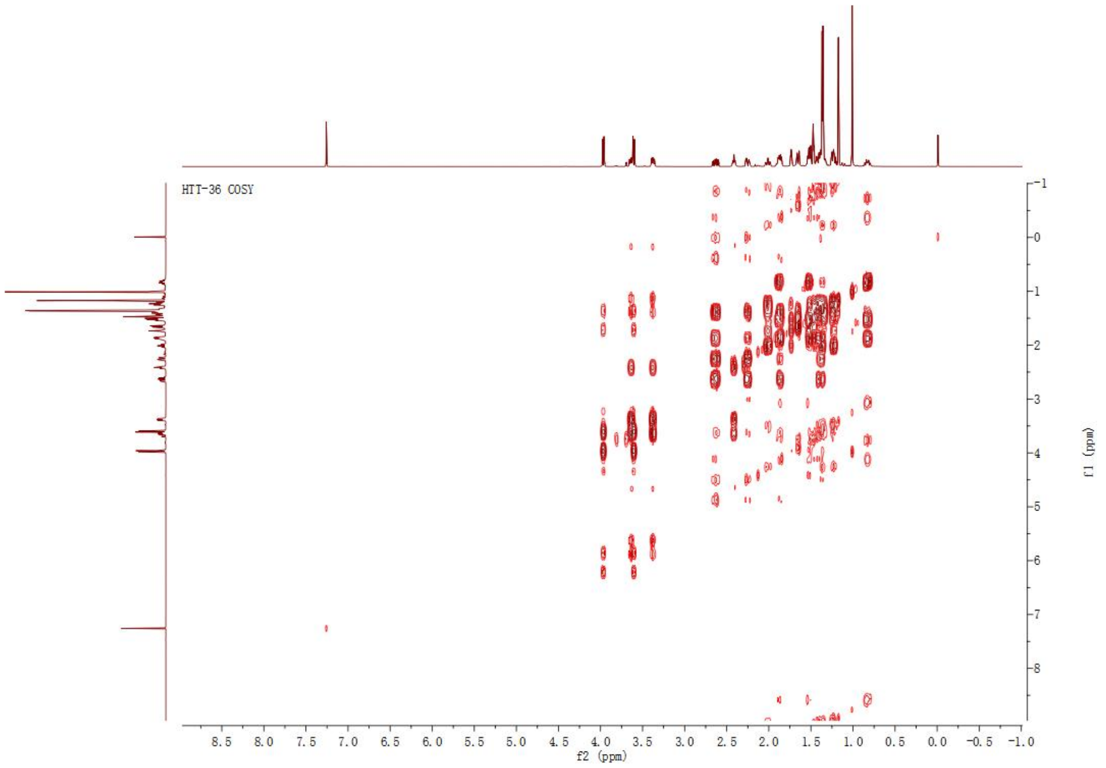

Figure SI82. ROESY spectrum of compound **9** ($CDCl_3$)

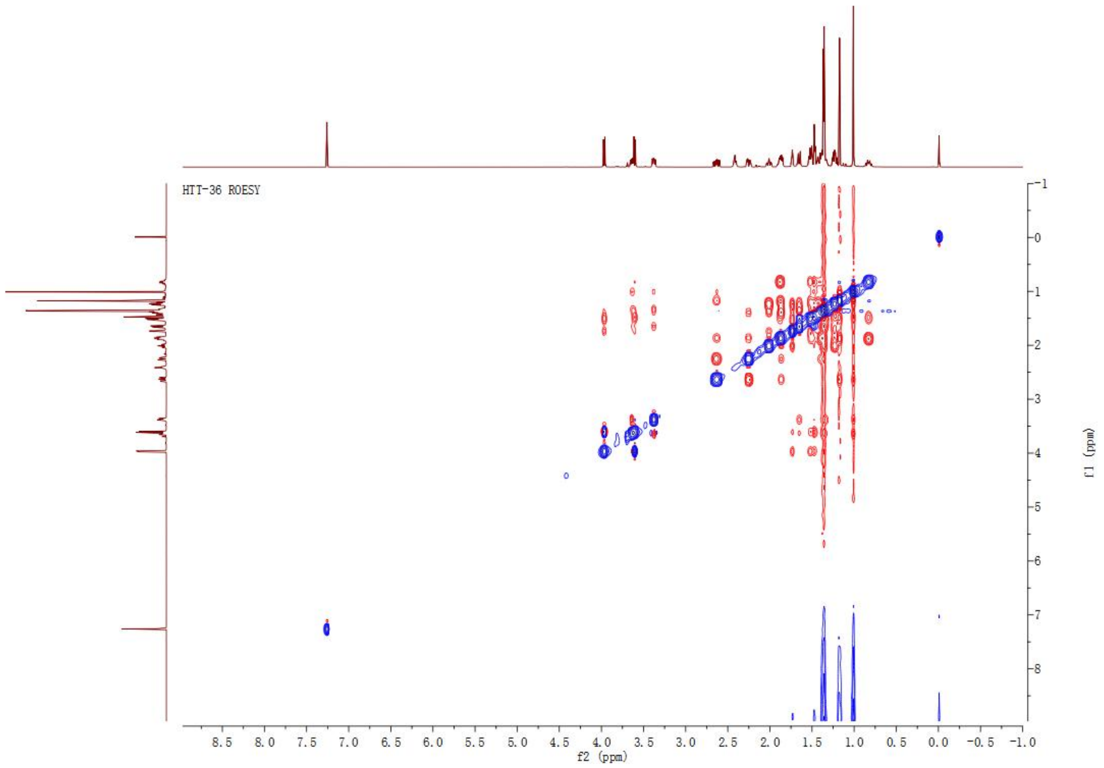

3. Experimental Section

Figure SI83. MST analysis report of compound **1**

Analysis Report

Analysis Name:	Analysis-Set #2
Type of Analysis:	MST
Evaluation strategy:	On Time
Cold Region Start:	-1 s
Cold Region End:	0 s
Hot Region Start:	4 s
Hot Region End:	5 s
Exported on:	Tue, 18 Jul 2023 14:07:56 GMT
Software Version:	MO.Affinity Analysis v3.0.5

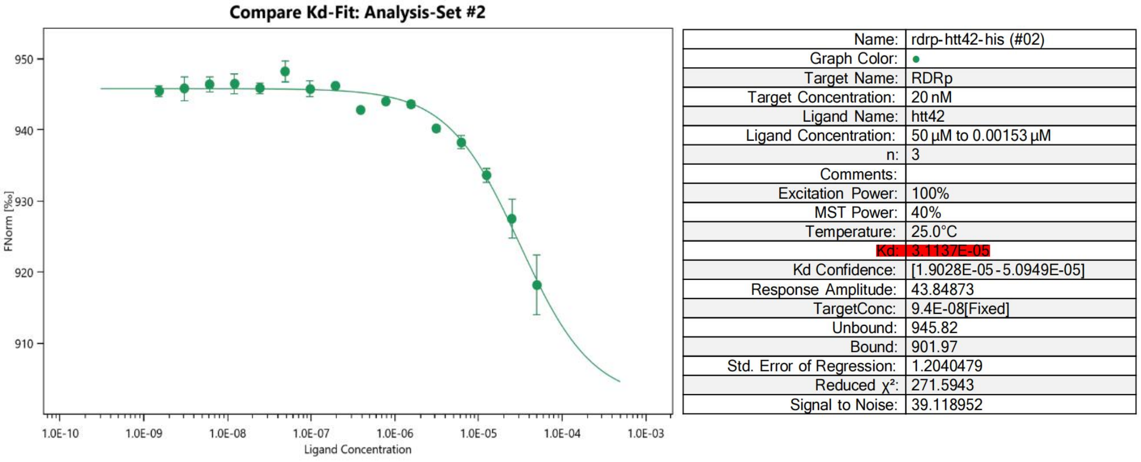

Name:	rdrp-htt42-his (#02)
Graph Color:	•
Target Name:	RDRp
Target Concentration:	20 nM
Ligand Name:	htt42
Ligand Concentration:	50 µM to 0.00153 µM
n:	3
Comments:	
Excitation Power:	100%
MST Power:	40%
Temperature:	25.0°C
Kd:	3.1137E-05
Kd Confidence:	[1.9028E-05 - 5.0949E-05]
Response Amplitude:	43.84873
TargetConc:	9.4E-08[Fixed]
Unbound:	945.82
Bound:	901.97
Std. Error of Regression:	1.2040479
Reduced χ²:	271.5943
Signal to Noise:	39.118952

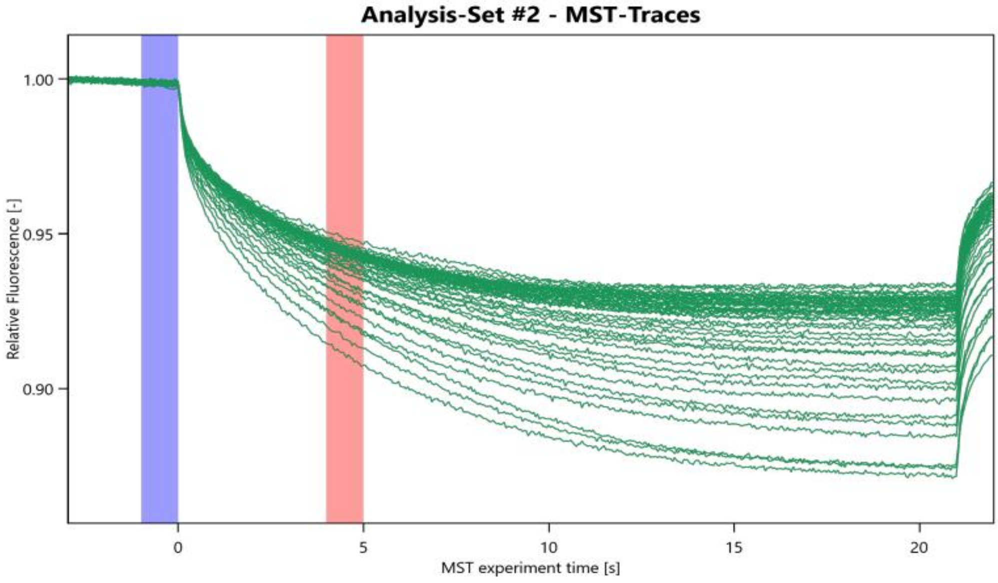

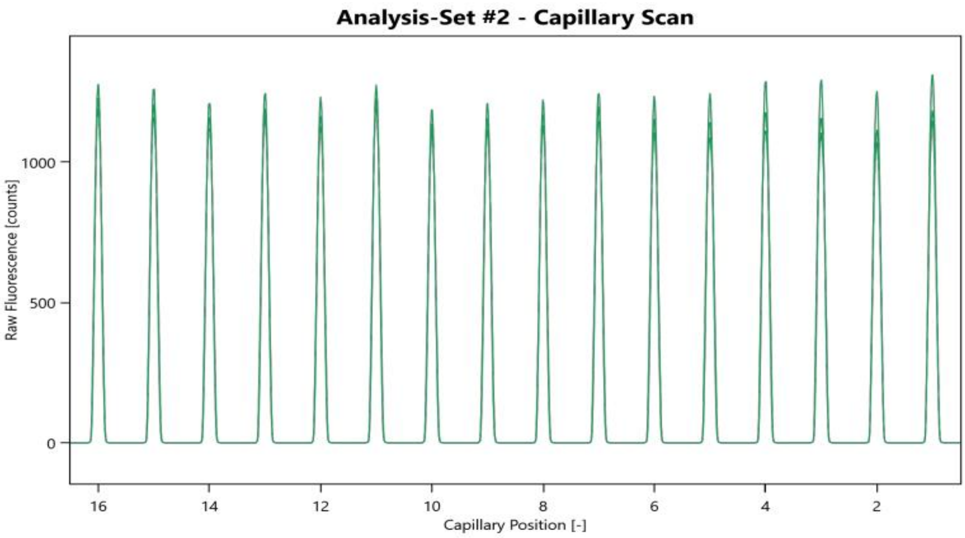

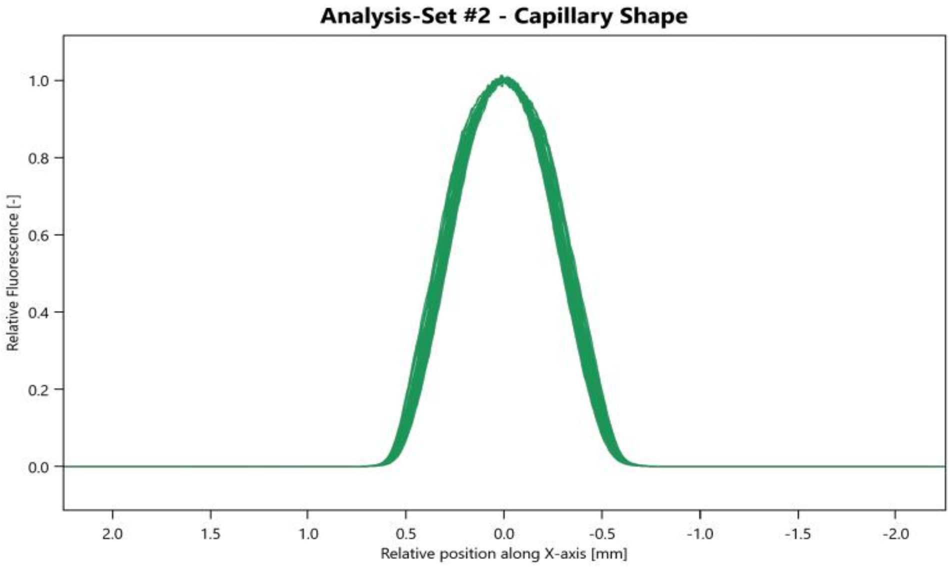

Raw data of merged Dose-Response #1

rdrp-htt42-his (#02)

Dose	Response (Average)	Std. Dev.	n
5E-05	918.17412	4.22815	3
2.5E-05	927.49845	2.70469	3
1.25E-05	933.6375	0.96419	3
6.25E-06	938.23904	0.90461	3
3.125E-06	940.21201	0.46106	3
1.5625E-06	943.61877	0.48571	3
7.8125E-07	944.0106	0.23463	3
3.90625E-07	942.81745	0.04164	3
1.953125E-07	946.2108	0.23015	3
9.765625E-08	945.74906	1.11765	3
4.8828125E-08	948.23162	1.52026	3
2.4414062E-08	945.89271	0.75119	3
1.2207031E-08	946.50979	1.41122	3
6.103516E-09	946.41558	1.11268	3
3.051758E-09	945.85039	1.67532	3
1.525879E-09	945.46582	0.74844	3
